# pH-dependent activation of the Na^+^/H^+^ antiporter NhaA and conformational dynamics of its N-terminus

**DOI:** 10.1038/s41467-026-73424-2

**Published:** 2026-06-12

**Authors:** Tsai-Hsuan Weng, Balázs Fábián, Elena Olkhova, Sonja Welsch, Sarah Luise Schmidt, Tsafi Danieli, Yael Keren, Abraham Rimon, Schara Safarian, Gerhard Hummer, Etana Padan, Hartmut Michel

**Affiliations:** 1 Emeritus Group Molecular Membrane Biology, Max Planck Institute of Biophysics, Frankfurt, Germany; 2 Department of Theoretical Biophysics, Max Planck Institute of Biophysics, Frankfurt, Germany; 3 Central Electron Microscopy Facility, Max Planck Institute of Biophysics, Frankfurt, Germany; 4 The Protein Production Facility, Alexander Silberman Institute of Life Sciences, the Hebrew University of Jerusalem, Jerusalem, Israel; 5 Department of Biological Chemistry, Alexander Silberman Institute of Life Sciences, the Hebrew University of Jerusalem, Jerusalem, Israel; 6 Fraunhofer Institute for Translational Medicine and Pharmacology ITMP Frankfurt, Frankfurt, Germany; 7 Fraunhofer Cluster of Excellence for Immune Mediated Diseases CIMD, Frankfurt, Germany; 8 Institute of Clinical Pharmacology, Goethe University Frankfurt, Frankfurt, Germany; 9 Institute of Biophysics, Goethe University Frankfurt, Frankfurt, Germany; 10 Present Address: Center for Life Sciences, Paul Scherrer Institute, Villigen PSI, Würenlingen, Switzerland

**Keywords:** Cryoelectron microscopy, Molecular conformation, Membrane proteins

## Abstract

Na⁺/H⁺ antiporters are vital for regulating intracellular pH and sodium ion levels across all domains of life. In *Escherichia coli*, NhaA is the principal Na⁺/H⁺ antiporter, exhibiting strong pH sensitivity and rapid turnover, yet the structural transitions underlying its activation and substrate recognition have remained obscure. Here, we use single-particle cryo-electron microscopy to determine the conformational ensemble of NhaA across a physiological pH range and in the presence of Na⁺, complemented by constant-pH molecular dynamics simulations. High-resolution structures of apo and Na⁺-bound NhaA reconstituted in lipid nanodiscs reveal progressive opening of the cytoplasmic funnel with increasing pH. We also visualize the previously unresolved N-terminal tail, which forms a dynamic plug at the cytoplasmic entrance under low-pH conditions and disengages at alkaline pH, coinciding with activation. The Na⁺-bound structure captures Na⁺ coordination at the ion-binding site, and simulations suggest potential roles for the conserved charged residues. Together, these findings illuminate how pH sensing, N-terminal gating, and substrate binding are structurally coordinated in NhaA, providing a framework for understanding Na⁺/H⁺ antiporter activation and regulation, and the basis for targeting clinical important antiporters.

## Abstract (web summary)

Na⁺/H⁺ antiporters regulate cellular pH and sodium balance. Here, the authors determine cryo-EM structures of NhaA and combine them with simulations to reveal pH-dependent activation, Na⁺ binding, and a regulatory N-terminus controlling transport.

## Introduction

Na^+^/H^+^ antiporters are found in membranes of nearly all prokaryotic and eukaryotic cells^1–3^. They are essential for the homeostasis of pH and sodium ion concentration in the cytoplasm and intracellular organelles^4^, and consequently support volume regulation and cellular bioenergetics^1,5^. Antiporters are an ancient form of transport proteins, having emerged early in the history of life, before the split between bacteria and archaea^3^. Malfunctions in several antiporters cause disease and have made them long-standing drug targets. The human NHE1, for instance, is implicated in heart failure^6,7^. Furthermore, Na^+^/H^+^ antiporters are critical for salt resistance in plants, an important trait given the spread of arid soils and droughts^8^.

NhaA is the main Na^+^/H^+^ antiporter of *Escherichia coli* and has homologues in enterobacteria, including many pathogens and other bacteria^1,9–12^. Certain human homologues of NhaA are potential drug targets because they are involved in essential hypertension, diabetes, and cancer^13–15^. NhaA exchanges two protons for one Na^+^ or Li^+^ ion at a very high rate^16,17^, and is highly sensitive to cytoplasmic pH, a property it shares with other prokaryotic and eukaryotic Na^+^/H^+^ antiporters^1^. Below pH 6.5, NhaA is inactive, and its activity increases above pH 6.5, reaching the maximal activity at pH 8.5^18^.

**Fig. 1:**
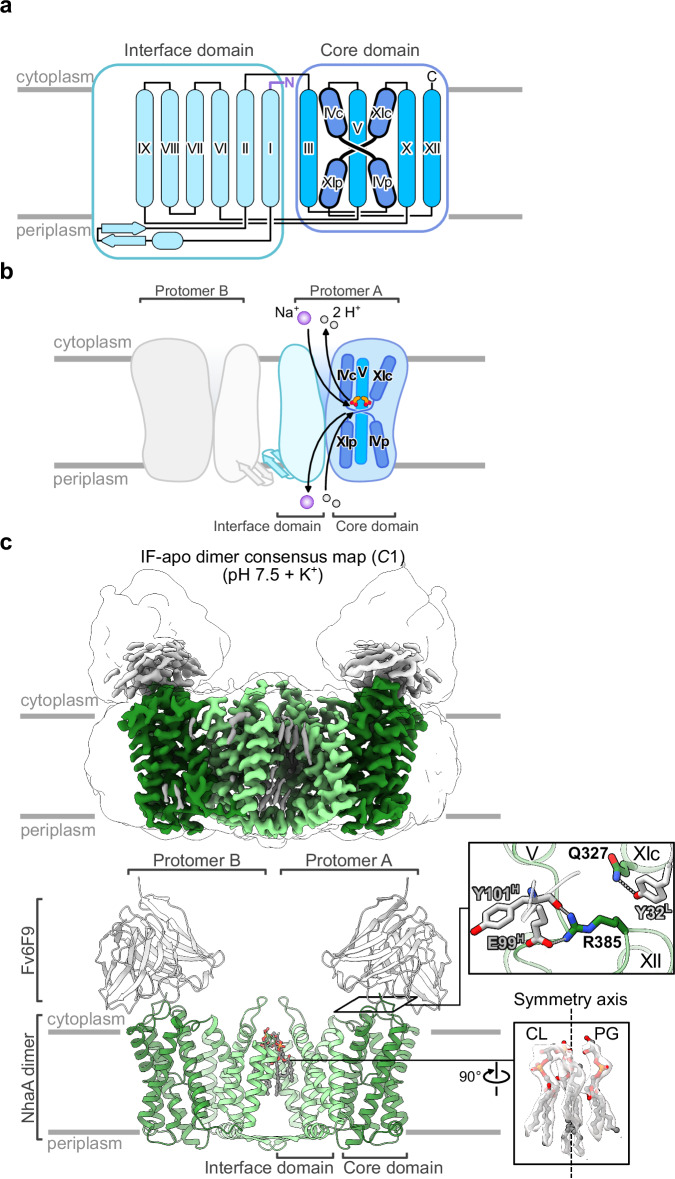
Architecture of bacterial Na^+^/H^+^ antiporter NhaA. **a** Schematic diagram of NhaA showing the topology of the secondary structure. The two crossing unwound TMs IV and XI of the NhaA fold are depicted in dark blue. **b** Schematic representation of NhaA homodimer. TMs IV, V and XI are shown for protomer A, with the two ion-binding residues Asp163 and Asp164 on TM V in orange. **c** Cryo-EM consensus map (top) and atomic model (bottom) of IF-apo NhaA dimer in nanodisc at pH 7.5 in the presence of K^+^. Densities for NhaA are colored in different shades of green, Fv6F9 in light gray, and lipid/non-protein densities in dark gray. A transparent cryo-EM density low-pass filtered at 6 Å is shown to visualize Fv6F9 and the nanodisc surrounding the transmembrane region. Residues participating in NhaA-Fv6F9 interaction are shown as sticks (top-right). The phospholipids at the dimer interface are in ball-and-stick representation and their cryo-EM densities are shown (bottom-right). H, Fv6F9 heavy chain; L, Fv6F9 light chain; CL, cardiolipin; PG, phosphatidylglycerol. Amino acid residues are labeled using one-letter codes in all figures.

Although the first *E. coli* NhaA crystal structure was obtained at pH 4.0, where the protein is downregulated, it has provided numerous insights into the structure, mechanism of action, and pH-regulation of Na^+^/H^+^ antiporters^19^. NhaA forms a dimer^20^, but its functional unit is the monomer^21^. The crystal structure of the monomer shows that amino acid residues 9–384 of NhaA are organized into 12 transmembrane (TM) helices, arranged in two domains: an interface domain, that mediates dimer interaction of two NhaA protomers (TMs I, II, VI–IX), and a core domain, which is involved in ion transport (TMs III–V and X–XII)^19^ (Fig. 1a, b). At the dimer interface, a β-hairpin between TMs I and II from each protomer forms an interfacial β-sheet at the periplasmic side, which has been suggested to stabilize the dimeric assembly^21,22^ (Fig. 1a, b). The two domains encompass a large cytoplasmic funnel (lined by TMs II, IVc, V, and IX) and a shallow periplasmic funnel (lined by TMs II, VIII, and XIp) with a hydrophobic barrier separating the two funnels (Fig. 1b). Despite very low sequence homology between TMs III–V and TMS X–XII, they are arranged as topologically inverted repeats. Furthermore, TMs IV and XI are unwound to extended chains that cross each other in the middle of the membrane, each leaving two short helices oriented either toward the cytoplasm (c) or toward the periplasm (p) (Fig. 1a, b). The partial positive charges of the N-termini and the partial negative charges of the C-termini of the short helices facing toward the middle of the membrane were suggested to be electrically compensated by Lys300 (TM X) and Asp133 (TM IV), respectively^19^. This distinct TM IV/XI assembly creates a delicately balanced electrostatic environment in the middle of the membrane critical for activity^23^. The fold of NhaA is now recognized as one of the five major folds found among prokaryotic secondary active transporters^24–31^. Despite marked differences in protein sequence, the NhaA fold has also been identified in many eukaryotic membrane transporters^32–34^.

In addition to the first crystal structure of the NhaA monomer, the crystal structure of the NhaA dimer was also obtained at pH 3.5, exhibiting the same inactive conformation^22^. Recently, two crystal structures of *E. coli* and *Salmonella enterica* NhaA were determined as a monomer at pH 6.5 (10% activity) at 2.2 Å resolution and as a dimer at pH 8.5 at 2.0 Å resolution, respectively^35,36^. Furthermore, a cryo-EM structure of the *E. coli* NhaA dimer was lately determined at pH 7.5 to 3.4 Å resolution^37^. However, despite being obtained in the presence of NaCl, these structures showed no bound Na^+^ and adopted conformations similar to the inactive NhaA in crystals at pH 3.5.

NhaA is classified as a secondary active transporter that operates via the canonical alternating access mechanism^38^. In this model, the ion-binding site is alternatively exposed to the cytoplasmic or periplasmic sides of the membrane, binding two H^+^ at one side and exchanging them with either one Na^+^ or one Li^+^ on the other. NhaA is electrogenic due to its unequal ion stoichiometry (2 H^+^/1 Na^+^), whereby the influx of two H^+^ is coupled to the export of one Na^+^, thus consuming the electrochemical proton gradient^16,17^.

Despite the insights gained from the previous NhaA structures, high-resolution snapshots capturing the conformational landscape during the transport cycle are required to elucidate the structure-function relationship of NhaA at the atomic level. In particular, Na^+^-bound structures are essential for understanding substrate recognition and its role in triggering ion exchange. Similarly, characterizing the conformational transitions that occur at alkaline pH is crucial for understanding how pH affects NhaA activity within the alternating access framework.

In this work, using single-particle cryogenic-electron microscopy (cryo-EM), we systematically determine the structure of Na^+^-bound NhaA at alkaline pH along with the apo conformations across the pH range where the inactive and active states intersect. Combined with constant-pH molecular dynamics (cpH-MD) simulations^39^, our structural data provide insight into the molecular mechanism of pH-dependent activation and substrate recognition in NhaA. We observe that the cytoplasmic funnel opens progressively with increasing pH. Furthermore, we unravel the structure of the N-terminal tail (NT), which had not been resolved before.

## Results

### Cardiolipin interactions at the dimer interface of NhaA

To study mechanistically important structural changes and the dimer architecture of NhaA, wildtype (WT) NhaA with a C-terminal six-histidine fusion was produced in *E. coli*, affinity-purified, and reconstituted into *E. coli* polar lipid nanodiscs using the membrane scaffold protein MSP1D1 (Supplementary Fig. 1). The variable fragment of a non-inhibitory monoclonal antibody 6F9 (Fv6F9), which binds to the dispensable C-terminal tail of native NhaA^40,41^, was used to form a complex with NhaA (Supplementary Fig. 1). The Fv served as a fiducial marker for better alignment and reconstruction in single-particle cryo-EM of the small membrane transporter.

We first focused on analyzing the dimer architecture of NhaA and the role of native lipids at the dimer interface. For this, we determined its cryo-EM structure at pH 7.5 in the presence of K^+^, a cation not bound by NhaA^42^, used here to maintain ionic strength (Fig. 1c, Supplementary Figs. 2 and 3, Supplementary Tables 1 and 2). The initial reconstruction, determined at 2.6 Å resolution with *C*2-symmetry applied, shows a symmetric NhaA dimer in the inward-facing (IF) conformation, with each protomer bound by one Fv6F9 at the C-terminus on the cytoplasmic side of the core domain (Fig. 1c, Supplementary Fig. 2a).

We next examined the role of native lipids at the dimer interface of NhaA. Cardiolipin (CL), a tetra-acyl lipid, has been shown to be essential for NhaA dimerization^43,44^. The previous cryo-EM structure of *E. coli* NhaA and the crystal structure of *S. enterica* NhaA, both in the dimeric form, have been reported with modeled CLs at the dimer interface^36,37^ (Supplementary Fig. 4a, b). However, the placement of this phospholipid differs between these structures, possibly due to low local resolution or high B-factors in the interface region. In our *C*2-symmetry-applied map, a non-protein density was observed at the dimer interface, exhibiting six extended tails resembling lipid acyl chains, positioned between Arg204 and Trp258 of the two protomers (Supplementary Fig. 4c). The density could not be fitted with either one or two CL molecules, suggesting an asymmetric lipid arrangement at the dimer interface. To separate possible asymmetric classes, a focused sorting with a mask covering the dimer interface was performed in *C*1 symmetry (Supplementary Fig. 2a). In all three resulting classes, asymmetric lipid occupancy was observed, featuring a CL-like density on one side and a diacyl phospholipid on the other (Supplementary Fig. 2a). Aligning the particles based on the CL density and refining without symmetry yielded a consensus NhaA dimer map at 2.7 Å resolution showing the asymmetrical dimer interface (Fig. 1c and Supplementary Figs. 2a and 4d). In this structure, one phosphatidic acid moiety of the CL sits close to the symmetric center of the NhaA dimer, while the other phosphatidic acid moiety and an additional diacyl phospholipid extend along the dimer interface (Fig. 1c, Supplementary Fig. 4d). Into the other phospholipid density, we could model a phosphatidylglycerol (PG), which has also been shown to reconstitute NhaA monomers into dimers^44^ (Fig. 1c and Supplementary Fig. 4d). The phosphate groups of the two phospholipids interact with the positively charged sidechains of Arg204 (loop VI–VII), Arg245 (loop VIII–IX) and Arg250 (TM IX), and their acyl chains make contacts with Trp258 (TM IX) on both NhaA protomers (Supplementary Fig. 4d). The placement of both CL and PG at the dimer interface in our high-resolution cryo-EM structure aligns closely to the *S. enterica* NhaA crystal structure (Supplementary Fig. 4b, d). Both protomers adopt nearly identical conformations with a C_α_ root mean square deviation (C_α_ RMSD) of 0.08 Å (Supplementary Fig. 5a). The only detectable difference is a slight shift of the Arg250 sidechain at the dimer interface, likely caused by asymmetric positioning of the CL phosphate headgroups (Supplementary Fig. 5b). As a result, both phospholipids together expand the contact area contributed by one protomer at the dimer interface from 661 Å^2^ (protein only) to at least 1,288 Å^2^ (including phospholipids), as calculated using the PISA server^45^ (see illustration in Supplementary Fig. 5c).

### Cryo-EM structures at different pH values uncover the previously unresolved N-terminal tail

**Fig. 2:**
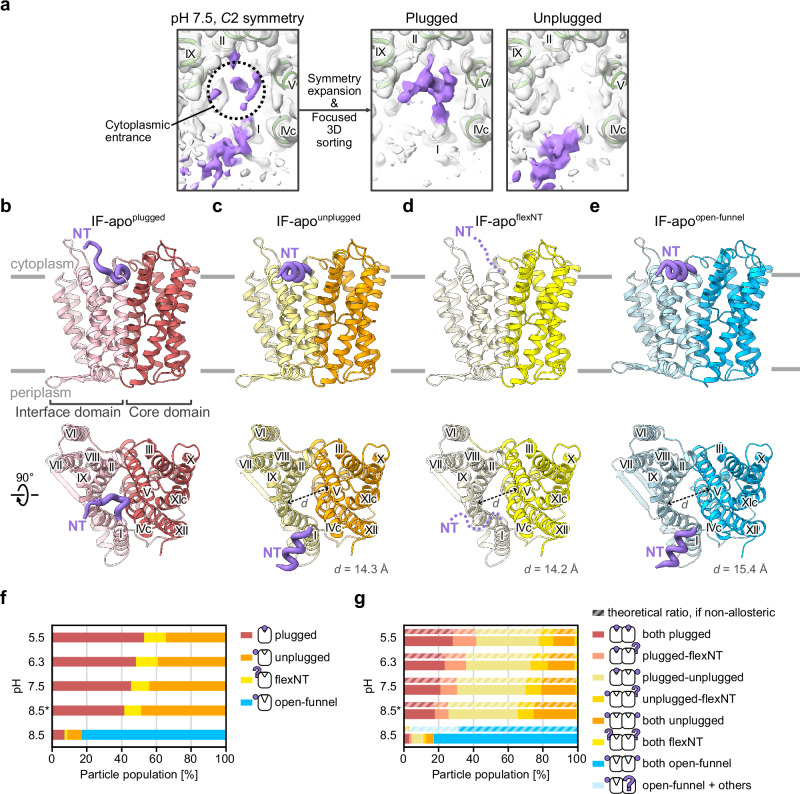
Structural heterogeneity and conformational ensemble of NhaA across a wide range of pH in the presence of K^+^. **a** Cryo-EM density, viewed from the cytoplasmic side, of the map with *C*2 symmetry applied (left), and the plugged and unplugged states (right) obtained after symmetry expansion and focused sorting using Relion. The density corresponding to the N-terminus (NT) is colored in purple. Atomic models of the NhaA protomer in the presence of K^+^, determined in the IF-apo^plugged^ (**b**), IF-apo^unplugged^ (**c**) and IF-apo^flexNT^ (**d**) states at pH 7.5, as well as the IF-apo^open-funnel^ state (**e**) at pH 8.5. The model of Fv6F9 is omitted here for clarity. The NT is shown in worm representation and colored in purple. The width of the cytoplasmic entrance (*d*) is measured as the distance between the C_α_ atoms of Met157 (TM V) and His256 (TM IX). **f** Particle population of NhaA protomers in different states at various pH values after the first round of 3D classification and symmetry expansion in single-particle cryo-EM analysis (see Supplementary Fig. 2). **g** Population of NhaA dimer in different states at various pH values. The theoretical ratios based on (**f**), assuming no allosteric effect between the two protomers, are put above the actual ratios for comparison as striped bars. *Particles of IF-apo^open-funnel^ state at pH 8.5 were excluded. Source data for (**f**, **g**) are provided as a Source Data file.

To dissect the molecular mechanism of NhaA transitioning from the inactive state at acidic pH, to the active state at alkaline pH, we determined the cryo-EM structures of NhaA-Fv6F9 in nanodisc across a wide range of pH values (5.5, 6.3, 7.5 and 8.5) in the presence of K^+^ (Supplementary Figs. 2a–d and 3, Supplementary Tables 1 and 2). Building on our initial processing of the pH 7.5 dataset, we applied *C*2-symmetry expansion, which allowed us to examine individual protomers within the dimer. This analysis revealed not only a strong density extended from TM I into the lipid-solvent interface, but also a pronounced density at the cytoplasmic entrance, weakly connected to TM I (Fig. 2a). To discern the identity of this unknown density, we classified the symmetry-expanded particles with a small mask encompassing the unknown density (Supplementary Fig. 2a–d). After intensive focused sorting, we obtained two high resolution classes: one with the density corresponding to the NhaA N-terminal tail (NT) forming a helix outside the cytoplasmic entrance (IF-apo^unplugged^), and the other one with a strong density in the cytoplasmic funnel connected to TM I, into which we were able to build the N-terminal residues 1–9 (IF-apo^plugged^) (Fig. 2a–c and Supplementary Fig. 6). The overall structures of both states are highly similar, with the exception of the NT (Fig. 2b, c). The same density in the cytoplasmic entrance was also observed in cryo-EM samples prepared at pH 5.5, 6.3 and 8.5. To better probe the distinct states in other conditions, particles classified similar to IF-apo^plugged/unplugged^ from all datasets were pooled together, followed by 3D sorting focusing on the NT, and the sorted particles from the respective pH were refined separately (Supplementary Fig. 2a–d). Both IF-apo^plugged^ and IF-apo^unplugged^ states were determined from samples prepared at all pH values in the presence of K^+^ (Fig. 2b, c and Supplementary Figs. 2a–d, 3 and 7). In addition, a minor class (9.6–12.7%) with no NT density was observed at all pH values, indicating a flexible NT (IF-apo^flexNT^) (Fig. 2d, Supplementary Figs. 2a–d, 3, 7a and 8). This minor class likely represents a transition state-like intermediate state between IF-apo^plugged^ and IF-apo^unplugged^. In the pH 8.5-K^+^ sample, besides the IF-apo^plugged^, IF-apo^unplugged^ and IF-apo^flexNT^ states, we identified another inward-facing apo structure featuring a more open cytoplasmic entrance with the NT forming an unplugged helix (IF-apo^open-funnel^), resolved at 2.9 Å resolution (Fig. 2e). Structural comparison with previously determined NhaA structures at pH 6.5 and alkaline pH reveals that their cytoplasmic entrances are comparable to, or even narrower than, that of the IF-apo^unplugged^ state at pH 8.5 (Supplementary Fig. 9). Among all structures, the IF-apo^open-funnel^ state exhibits the most open cytoplasmic entrance (Supplementary Fig. 9). Notably, IF-apo^open-funnel^ is the predominant state in the pH 8.5-K^+^ dataset and is exclusively observed in dimeric particles where both protomers adopt the same conformation. (Fig. 2f, g and Supplementary Fig. 2d). The single-particle cryo-EM analysis indicates that the particle population gradually shifts from IF-apo^plugged^ toward IF-apo^unplugged^ as pH increases from 5.5 to 7.5, and is then dominated by IF-apo^open-funnel^ at pH 8.5 (Fig. 2f). Furthermore, the N-terminal residues 1–6 could not be resolved in the IF-apo^plugged^ NhaA structure at pH 8.5, suggesting a less stable interaction that may favor NT unplugging at alkaline pH (Supplementary Fig. 7b).

To assess whether the conformation of one protomer allosterically affects the other one within a NhaA dimer, the symmetry-expanded sub-particles were mapped back to the original particles, and the population of distinct dimer states was quantified (Fig. 2g). The population of various NhaA dimer states indicates that the position of the NT in one protomer has no allosteric effect on the NT positioning in the adjacent protomer (Fig. 2g). However, at pH 8.5, a protomer in the IF-apo^open-funnel^ state allosterically affects the adjacent protomer, as only IF-apo^open-funnel^ dimer, but not mixed conformations of IF-apo^open-funnel^ and the other states, could be obtained from the pH 8.5-K^+^ sample (Fig. 2g and Supplementary Fig. 2d). Although the monomeric NhaA variants with β-hairpin disruption exhibit comparable antiport activity to the WT dimer, cells expressing the WT dimer show greater tolerance to extreme salinity at alkaline pH than those expressing monomeric mutants^21,46^. This enhanced tolerance may result from the allosteric effect conveyed through the β-hairpin, which stabilizes the IF-apo^open-funnel^ state.

### Structural basis of Na⁺ recognition by NhaA revealed by cryo-EM

**Fig. 3:**
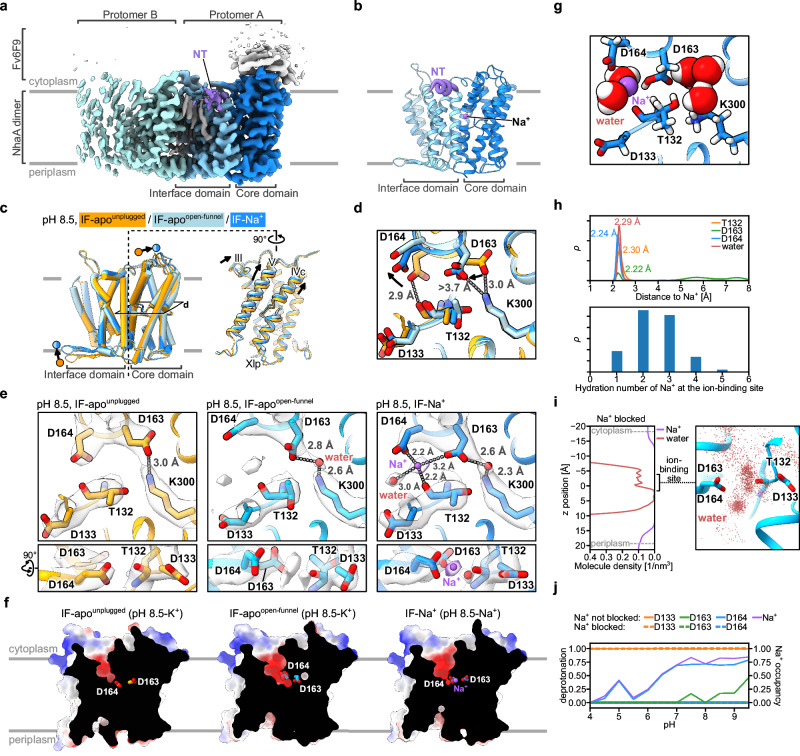
Na^+^ binds to the ion-binding site of NhaA. **a** Cryo-EM map of IF-Na^+^ NhaA in nanodisc at pH 8.5. Densities for NhaA are colored in different shades of blue (protomer A) and cyan (protomer B), Fv6F9 in light gray, and lipid/non-protein densities in dark gray. **b** Atomic model of IF-Na^+^ NhaA protomer. Color code is same as in (**a**). The model of Fv6F9 is omitted here for clarity. **c** Structural comparison of the IF-apo^unplugged^, IF-apo^open-funnel^ and IF-Na^+^ NhaA at pH 8.5, superimposed on the interface domain. General movements of the structures are marked by arrows with circles filled with colors corresponding to different states. **d** Structural comparison of the ion-binding site indicated in (**c**) with the same color code. The ion-binding residues are shown as sticks. **e** Cryo-EM maps and models of the ion-binding site corresponding to the states in (**d**). All maps are sharpened with the same B-factor of −70, low-pass filtered to 3.0-Å, and adjusted to similar threshold. Water (red) and Na^+^ (purple) are in ball representation. **f** Cut-away views of IF-apo^unplugged^, IF-apo^open-funnel^ and IF-Na^+^ NhaA at pH 8.5 in surface representation showing the cytoplasmic funnel. The ion-binding residues Asp163 and Asp164 are shown as sticks. **g** Snapshot of the ion-binding site with a bound Na^+^ ion in MD simulation at pH 8.5. **h** Probability density (*ρ*) of the coordination (top) and the hydration number (bottom) of the Na^+^ ion at the ion-binding site at pH 8.5 in cpH-MD simulations. **i** Water density profile normal to the lipid bilayer (left). The right panel shows the aggregated positions of water molecules near the ion-binding site in the simulations with Na^+^ blocked from entering the cytoplasmic funnel. **j** Protonation states of the ion-binding residues and Na^+^ occupancy of the ion-binding site in cpH-MD simulations as function of pH. Dashed and solid lines show results from simulations with and without Na^+^ blocked at the cytoplasmic funnel, respectively. Separate plots displaying the standard deviations of Na^+^ occupancy and the deprotonation states of Asp163 and Asp164 without Na^+^ blocked are provided in Supplementary Fig. 20. Source data are provided as a Source Data file.

Sequence conservation and mutagenesis studies indicate that NhaA binds Na^+^ through the conserved residues Asp163, Asp164 and Thr132^42,47–50^. To investigate how NhaA recognizes its substrate at molecular level, we determined the cryo-EM structure of NhaA-Fv6F9 in nanodiscs at pH 8.5 in the presence of Na^+^ using the same symmetry expansion approach as in the K^+^-supplemented datasets (Fig. 3a, b and Supplementary Figs. 2e and 3). An inward-facing NhaA protomer structure was determined at 3.0 Å resolution, showing close resemblance to the conformation of IF-apo^open-funnel^ NhaA with a C_α_ RMSD of 0.3 Å (Fig. 3c). This Na^+^-supplemented NhaA structure exhibits the same NT helix pointing outside the cytoplasmic entrance as in the IF-apo^open-funnel^ state (Fig. 3a, b).

Compared to IF-apo^unplugged^ NhaA, the β-hairpins at the dimer interface of both the IF-apo^open-funnel^ and Na^+^-supplemented structures tilt toward the lipid bilayer (Fig. 3c). Concurrently, the cytoplasmic half of the core domain moves away from the cytoplasmic entrance, particularly with TM V shifting ~2 Å toward the cytoplasmic side (Fig. 3c), followed by disruption of the Asp163–Lys300 interaction and subsequent opening of the ion-binding site (Fig. 3d, e). As a result, the cytoplasmic funnels in both the IF-apo^open-funnel^ and Na^+^-supplemented structures are wide open and expose the putative ion-binding residues Asp163 and Asp164 (Fig. 3f). The ion-binding site of the Na^+^-supplemented structure, resolved locally at ~2.9 Å, shows a clear spherical extra density between the putative ion-binding residues Asp163 and Asp164 on TM V as well as Thr132 on loop IVp–IVc (Fig. 3e and Supplementary Fig. 10a). In contrast, the ion-binding site of the IF-apo^open-funnel^ structure shows an elongated density, resolved locally at ~2.85 Å, likely emerging from water molecules entering the binding site (Fig. 3e and Supplementary Fig. 10b). This elongated density cannot be attributed to K^+^, as the distances of the density to the carboxyl groups of Asp163 and Asp164 exceed 4 Å, which is longer than a typical K^+^–Asp coordination distance (~2.8 Å)^51^. Furthermore, NhaA neither binds nor transports K^+^^42,52^. This difference in cryo-EM density at the putative ion-binding site suggests that the spherical extra density in the Na^+^-supplemented NhaA structure is a Na^+^ ion, bound between the carboxyl groups of Asp163 and Asp164, the backbone carbonyl oxygen of Thr132, and a water molecule (Fig. 3e). Consistently, the observed Na^+^–ion-binding residue distances match typical Na^+^ coordination geometry (~2.4 Å to Asp and ~2.4 Å to the Thr backbone carbonyl)^51^ (Fig. 3e). To confirm this, we performed cpH-MD simulations^39^ between pH 4.0 and 9.5, either with Na^+^ moving freely throughout the simulation box, or with Na^+^ blocked from entering the cytoplasmic funnel (Supplementary Fig. 11). The results show that the Na^+^ ions can move into the funnel, and once a Na^+^ ion enters the ion-binding site, it stays stably coordinated with Asp163, Asp164 and Thr132, as well as water molecules (Fig. 3g, h, Supplementary Movie 1). On the other hand, the simulation with Na^+^ blocked from the cytoplasmic funnel shows that water enters freely from the cytoplasmic entrance and constantly occupies the ion-binding site (Fig. 3i). Previous mutagenesis analyses showed that substitutions at Asp163 and Asp164 abolish Na^+^ or Li^+^ binding and transport, whereas mutations at Thr132 only reduce activity, consistent with our structural observations that Thr132 coordinates Na^+^ via its backbone carbonyl rather than the sidechain^42,47–49^. Together, the cryo-EM structures and the MD simulation data suggest that the Na^+^-supplemented NhaA structure represents the inward-facing, Na^+^-bound state (IF-Na^+^), and the IF-apo^open-funnel^ structure is an active, apo state. In our MD simulations, the distance between Asp163 and Lys300 increases as Na^+^ enters the ion-binding site (Supplementary Fig. 12), which generally aligns with the increased Asp163–Lys300 distance observed between the IF-apo^unplugged^ state and the active IF-apo^open-funnel^ and IF-Na^+^ states. The simulations indicate a strong preference for an ion-binding site in which Asp163, Asp164, and Lys300 are all protonated, and Asp133 is deprotonated in the absence of Na^+^ (Fig. 3j). The arrival of Na^+^ triggers deprotonation of the aspartates, first Asp164 and then Asp163 (Fig. 3j). The protonation states of Asp133 and Lys300 are stabilized by their electrostatic interactions with the positively charged N-termini of TMs IVc and XIp, and the negatively charged C-termini of TMs IVp and XIc, respectively (Supplementary Fig. 13a,b). When Na^+^ entry is blocked, Asp164 remains protonated due to its predominantly hydrophobic environment and limited hydration (<2.0 waters) (Supplementary Fig. 13c). Asp163 also remains protonated without Na^+^ at the ion-binding site, forming hydrogen bonds with the hydroxyl group of Thr132 and the backbone of Ala130, although it occasionally reorients toward a more hydrophobic region (Supplementary Fig. 13d).

### A pH increase induces cytoplasmic funnel opening in NhaA via structural changes in the pH sensor

**Fig. 4:**
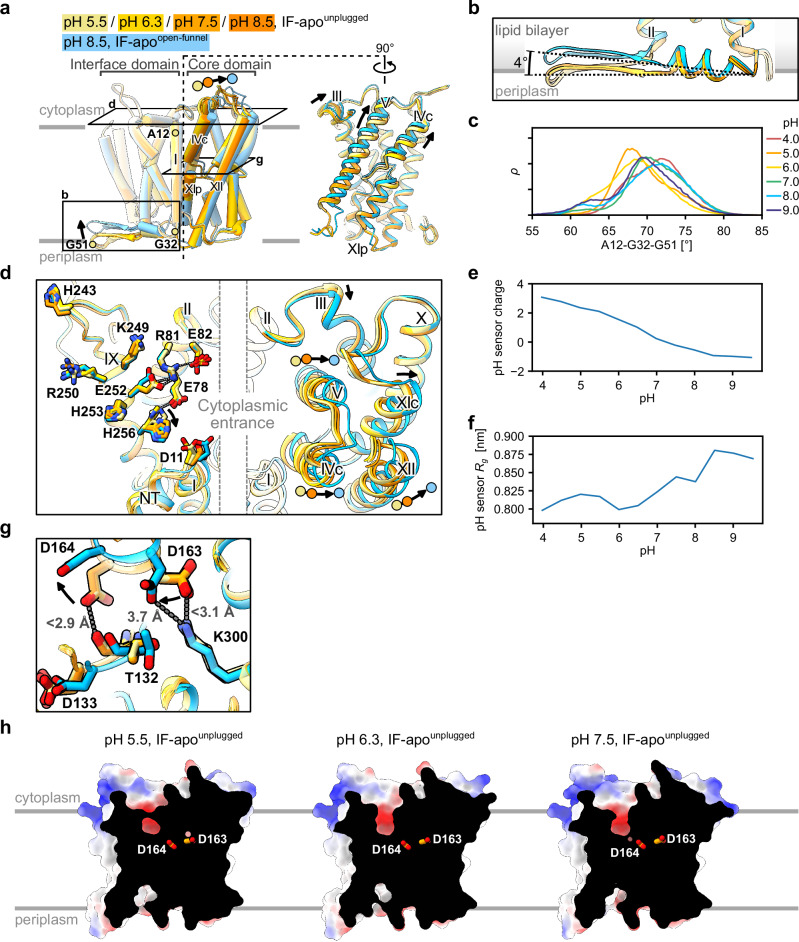
Cytoplasmic funnel of NhaA gradually opens as pH increases. **a** Structural comparison of the IF-apo^unplugged^ states of NhaA at the indicated pH, as well as the IF-apo^open-funnel^ state at pH 8.5, superimposed on the interface domain. General movements of the structures are marked by arrows with circles filled with colors corresponding to different states. **b** Close-up view of the movement of the β-hairpin in the interface domain. **c** MD simulation data showing the changes of the angle between TM I and β-hairpin (Ala12–Gly32–Glu51) at different pH values as probability density (*ρ*). **d** Ribbon representation of the IF-apo^unplugged^ states at different pH values and the IF-apo^open-funnel^ state at pH 8.5 showing the cytoplasmic entrance and the pH sensor residues (sticks). The models are colored same as in (**a**). The left panel shows the changes of the sidechains of the pH sensor residues. The right panel displays the movement of the TMs in the core domain. **e** MD simulation data showing the change of the total charges of pH sensor residues across different pH values. **f** MD simulation data showing the change of the radius of gyration (*R*_*g*_) of the pH sensor residues across different pH values. **g** Structural comparison of the ion-binding site of the IF-apo^open-funnel^ and IF-apo^unplugged^ states at different pH values. The ion-binding residues are shown as sticks with the same color code as in (**d**). **h** Cut-away views of the IF-apo^unplugged^ states at different pH values in surface representation showing the cytoplasmic funnel. The ion-binding residues Asp163 and Asp164 are shown as sticks. Source data for (**e**, **f**) are provided as a Source Data file.

The Na^+^/H^+^ antiport activity of NhaA gradually increases above pH 6.5, reaching the highest activity at pH 8.5^17,53^. Structural comparisons of the IF-apo^unplugged^ states at different pH values show the opening of the cytoplasmic funnel as the pH increases (Fig. 4a). Particularly, the conformation of IF-apo^open-funnel^ NhaA, the major population at pH 8.5, indicates a wider opening of the cytoplasmic half of the core domain, with TMs V and IVc shifting 2.2 and 1.9 Å towards the cytoplasm, respectively (Fig. 4a). In addition, the β-hairpin at the periplasmic side of the dimer interface tilts by 4° toward the lipid bilayer (Fig. 4b). A similar tilt is also observed in the MD simulations as the pH increases (Fig. 4c). Since the β-hairpins of the two NhaA protomers form a joined β-sheet, tilting of one β-hairpin may mediate the allosteric effect observed in the IF-apo^open-funnel^ state.

The pH sensor—a cluster of tightly interacting charged residues (Asp11, Glu78, Arg81, Glu82, His243, Lys249, Arg250, Glu252, His253, and His256) localized near the entrance of the NhaA cytoplasmic funnel—has been suggested to sense the intracellular pH and conveys the signal to the ion-binding site, activating the antiporter^54–58^. In our cryo-EM structures of the unplugged states, the pH sensor residues appear to be responsible for regulating the opening of the cytoplasmic entrance. As the pH increases, His256 moves toward TM I and breaks the salt bridge with Glu78 (Fig. 4d), similar to the previous crystal structure at pH 6.5^35^. The cpH-MD simulations report p*K*_a_ values for Glu78 (6.9), His243 (6.0), His253 (5.7), and His256 (7.1) in the physiological range (Supplementary Fig. 14 and Supplementary Table 3). While the values for histidines are in good agreement with those obtained by Huang et al. (Glu78: 3.1; His243: 6.8; His253: 6.3; His256: 6.9)^59^, our calculations support a more active role for Glu78 in pH sensing. Glu78 starts titrating at acidic pH and becomes deprotonated at alkaline pH, while His256 remains positively charged at lower pH and becomes fully deprotonated above pH 8.0, suggesting a transient Glu78–His256 salt bridge at lower pH, which breaks at higher pH values (Supplementary Fig. 15). Supporting this, the previous study shows that Glu78Ala mutant has a different pH activation profile than the WT^35^. The comparably large scatter in the deprotonation values of Glu78 across the different replicates mostly reflects differences in the initial structures, being about 0.5 p*K*_a_ units lower when starting from the low pH crystal structure (PDB 4AU5)^22^ compared to the new IF-Na^+^ structure (Supplementary Fig. 14a).The cytoplasmic half of the core domain also gradually moves away from the cytoplasmic entrance as the pH increases (Fig. 4d). Simulation data show that the total charge of the pH sensor changes from +3 at pH 4.0, to 0 at pH 7.0, reaching −1 at pH 8.5, favoring the entry of Na^+^ ions (Fig. 4e). Alongside the change of electrostatic potential at the cytoplasmic entrance, the entrance also becomes more open as the radius of gyration of the pH sensor increases (Fig. 4f). Our data together thus suggest a wider opening of the cytoplasmic entrance at alkaline pH, as indicated by a previous simulation study^59^.

A pH-dependent conformational change is also observed at the ion-binding site of the cryo-EM structures (Fig. 4g, Supplementary Fig. 16). Below pH 7.5, the Asp163–Lys300 and Asp164–Thr132 interactions remain as observed in the previous inactive crystal structures. However, at pH 8.5, over 80% of NhaA dimers undergo a conformational change, which not only opens the cytoplasmic entrance, but also the ion-binding site, thus transitioning to the IF-apo^open-funnel^ state (Figs. 2g and 4g). The movement in the ion-binding site may represent initiation of new conformational changes priming the translocation steps. Unlike in the IF-Na^+^ and the IF-apo^open-funnel^ states, there is no extra density observed between Asp163 and Lys300 in the IF-apo^unplugged^ states at all pH values (Fig. 3e and Supplementary Fig. 16). In the IF-apo^unplugged^ cryo-EM structures, the cytoplasmic funnel is progressively deepened as the pH increases, ultimately extending to Asp163 and Asp164 at pH 8.5 (Figs. 3f and 4h).

### Interactions between the N-terminal tail and the cytoplasmic funnel

**Fig. 5:**
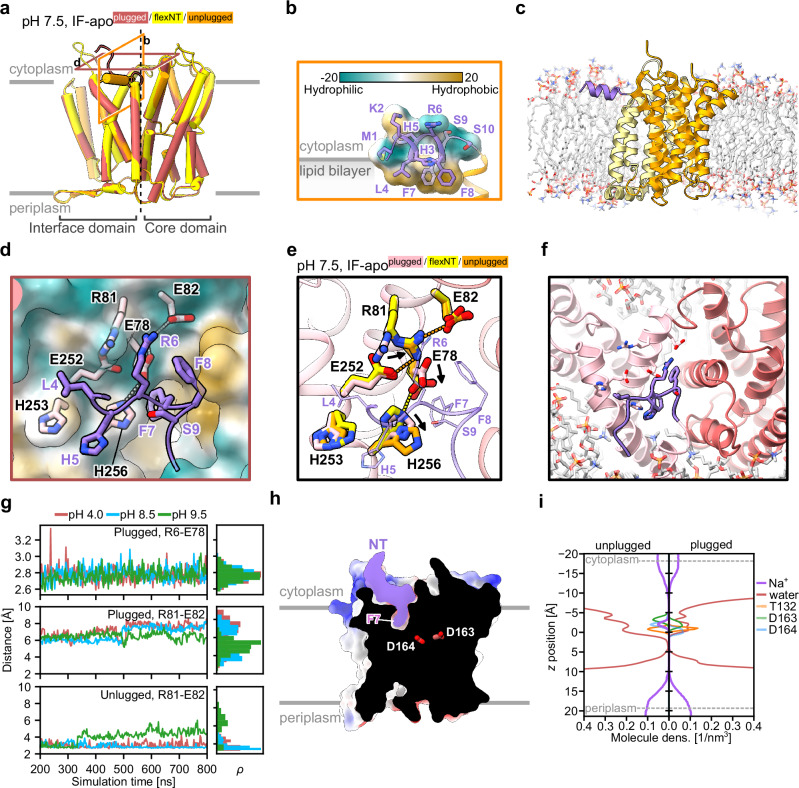
Interactions of the NT with the cytoplasmic funnel of NhaA. **a** Structural comparison of the IF-apo^plugged^, IF-apo^flexNT^ and IF-apo^unplugged^ states of NhaA at pH 7.5, superimposed on the interface domain. **b** Close-up views of the NT of the IF-apo^unplugged^ NhaA at pH 7.5, showing the surface of the NT colored by hydrophobicity. The residues of the NT are shown as sticks. **c** Snapshot of the unplugged NhaA in MD simulation, where the NT (purple) stays stably on the lipid bilayer throughout 1-μs duration. **d** Cytoplasmic view of the IF-apo^plugged^ state at pH 7.5, depicting the interaction between the NT and the cytoplasmic entrance, which is shown in surface representation colored by hydrophobicity with the same color key in (**b**). The residues of the pH sensor and the NT are shown as sticks. **e** Close-up view of the pH sensor residue movement between the IF-apo^plugged^, IF-apo^flexNT^ and IF-apo^unplugged^ states. For simplicity, only the backbone of IF-apo^plugged^ is shown in ribbon representation. **f** Snapshot of the IF-apo^plugged^ NhaA in MD simulation, where the NT (purple) interacts stably with the cytoplasmic entrance throughout 1-μs duration. **g** MD simulation data showing the interaction between the Arg6 (NT) and the pH sensor residue Glu78 in the IF-apo^plugged^ state, and the interactions between the pH sensor residues Arg81 and Glu82. **h** Cut-away view of the IF-apo^plugged^ NhaA at pH 7.5 in surface representation showing the cytoplasmic cavity. The NT is colored in purple. The ion-binding residues Asp163 and Asp164 are shown as sticks. **i**, Density of Na^+^ ions and water molecules along the z-axis across the lipid bilayer in MD simulations for the IF-apo^unplugged^ and IF-apo^plugged^ states.

Apart from the NT, the IF-apo^plugged^, IF-apo^unplugged^ and IF-apo^flexNT^ states of NhaA resemble each other at all pH values with a C_α_ RMSD of 0.4 Å or less across residues 10–388 (Fig. 5a). The NT of the IF-apo^unplugged^ state forms an amphipathic helix sitting on the cytoplasmic side of the lipid bilayer, pointing away from the cytoplasmic entrance (Fig. 5b). Simulation shows that this N-terminal amphipathic helix stays stably on the membrane throughout the entire 1 μs of simulation (Fig. 5c). Unlike the IF-apo^unplugged^ state, the NT of the IF-apo^plugged^ state forms a loop interacting with the cytoplasmic entrance, with Phe7 pointing inward, toward the hydrophobic section of the funnel (Fig. 5d). The NT also interacts with the pH sensor residues Glu78 and Glu82 by forming salt bridges with Arg6 (Fig. 5d). As the N-terminal plug moves out of the cytoplasmic entrance, Arg81 on TM II displaces Arg6, forming salt bridges with Glu82 and Glu252 (Fig. 5e). Multiple sequence alignment shows the NT, specially Arg6 and Phe7, to be highly conserved among enterobacteria (Supplementary Fig. 17). The N-terminal plug binds to the cytoplasmic entrance stably throughout the entire 1 μs of simulation at different pH values (Fig. 5f). The simulations also corroborate a stable interaction between Arg6 and Glu78 in the IF-apo^plugged^ state, and the formation of Arg81–Glu82 interaction when the plug is displaced (Fig. 5g). As a result, in the IF-apo^plugged^ state, the NT completely blocks the cytoplasmic funnel (Fig. 5h, Supplementary Fig. 18). At both acidic and alkaline pH, the simulations indicate that Na^+^ ions are excluded from the cytoplasmic funnel by the N-terminal plug (Fig. 5i). Our results are consistent with the previous cross-linking study, which has suggested a close proximity between Leu4 and Glu252 at neutral pH^60^. Furthermore, the monoclonal antibody 1F6 (mAb1F6), while recognizing the synthetic N-terminal fragment of NhaA (residues 3–10) independent of pH, binds NhaA with the highest affinity at alkaline pH^61^. This coincides with our cryo-EM analysis that the NT unplugs from the cytoplasmic entrance in over 90% of the particle population at pH 8.5, allowing the binding of mAb1F6 (Fig. 2f). Furthermore, Cys-replacement of Glu252 increases the apparent *K*_m_ for Na⁺ by 50-fold and shifts the pH dependence of NhaA activity by one unit toward the alkaline range^55^. Cys-replacement mutations of Glu82 and Glu78 produce similar effects^57^.

To explore the function of the NT of NhaA, we constructed a plasmid expressing mutated NhaA with residues 2–13 deleted (ΔNT). The growth phenotype of ΔNT-NhaA was tested in *E. coli* strain EP432, where both the Na^+^/H^+^ antiporter genes *nhaA* and *nhaB* are deleted^62^. This strain can grow in Luria broth with K^+^ replacing Na^+^ (LBK) but fails to grow in high-salt selective media. When transformed with plasmids encoding either WT- or ΔNT-NhaA, EP432 grew similarly under aerobic conditions in both LBK and selective Na^+^/Li^+^-rich media at pH 7.0 and 8.3, suggesting a similar growth phenotype of ΔNT to WT at neutral to alkaline pH (Supplementary Fig. 19a).

To further assess the function of the NT, we measured Na^+^/H^+^ antiport activity in everted membrane vesicles prepared from the EP432 cells producing either ΔNT- or WT-NhaA^63^. In this assay, the pH-sensitive fluorescent dye acridine orange was used to monitor H^+^ gradients. Upon addition of lactate, respiration drives H^+^ uptake into the vesicles, resulting in fluorescence quenching. Subsequent addition of Na^+^ activates the antiporter, which exports two H^+^ in exchange for one Na^+^, leading to fluorescence dequenching. At pH 8.5, the Na^+^/H^+^ antiport activity of ΔNT-NhaA (*K*_m_ = 0.290 ± 0.015 mM) was close to that of the WT (*K*_m_ = 0.2 mM)^49^ (Supplementary Fig. 19b). Also, ΔNT- and WT-NhaA exhibited similar antiport activity profiles between pH 6.5 and 9.0, as well as comparable protein expression in the membrane (Supplementary Fig. 19c). Overall, these results indicate that the Na^+^/H^+^ antiport activity of ΔNT-NhaA is functionally equivalent to that of the WT at neutral and alkaline pH.

## Discussion

Our cryo-EM structures of the Na^+^/H^+^ antiporter NhaA define key structural transitions that underlie its pH-dependent activation and substrate recognition. By overcoming the previous limitations for small membrane proteins using the fiducial marker Fv6F9 and reconstitution into *E. coli* lipid nanodiscs, we resolved both apo and Na^+^-bound states across a physiologically relevant pH range. Combined with cpH-MD simulations, our results reveal how the ion-binding site, the dynamic NT, and the pH sensor coordinate to regulate NhaA function.

A major advance of this study is the visualization of the previously unresolved NT, which adopts dynamic conformations depending on pH (Fig. 2). The roles of N- and C-terminal tails are often neglected in structural studies of transporters due to their flexible and disordered nature. However, these regions have been implicated in transporter folding, biogenesis, gating, and substrate specificity^64^. While the ΔNT-NhaA mutant retained WT-like growth and antiport activity at neutral and alkaline pH, the population of the IF-apo^plugged^ protomers, predominant at acidic pH, decreases gradually with the increasing pH. This pH-sensitive structural shift resembles the pH dependence of NhaA activity^16,17^. In several LeuT-fold transporters, the N-terminal tail has been shown to participate in cytoplasmic gating or even form a plug that inserts into the cytoplasmic funnel^65–67^. Notably, the cryo-EM structure of the mammalian Na^+^/H^+^ antiporter NHA2, which shares the same NhaA fold, shows a cytoplasmic N-terminal extension helix suggested to interact with lipids, and influence TM mobility and substrate access^32^. These examples may hint at a potential regulatory role for the NT of NhaA.

In the IF-apo^plugged^ state, the NT’s proximity to the pH sensor^54–58^, particularly Glu78 and Glu82 on TM II, raises the possibility of functional coupling, since TM II is the only TM that lines both cytoplasmic and periplasmic funnels and is important for ion translocation^57^ (Fig. 5). Cys-replacement of both Glu78 and Glu82 changes the pH dependence and reduces the antiport activity of NhaA^57^. Similarly, His3 and His5 mutations in the NT also shift pH-dependent activity^61^. Taken together, the NT might be a self-regulatory motif that blocks Na^+^ entrance to the cytoplasmic funnel. While it remains possible that the pH sensor alone fulfills pH-dependent activation^35^, the NT likely serves a reinforcing or fine-tuning role.

Despite several NhaA structures solved at active pH in the presence of Na^+^, they did not capture Na^+^ at the ion-binding site or reveal the fully open IF-apo^open-funnel^ state. In contrast, our cryo-EM structure at pH 8.5 clearly resolves a bound Na^+^ ion at the ion-binding site (Fig. 3). This likely reflects differences in sample conditions: we used nanodiscs with 300 mM NaCl or KCl and native *E. coli* lipids, whereas earlier studies employed detergents and lower salt concentrations^35–37^. The high-salt condition and native lipid environment might favor the opening of the cytoplasmic funnel, therefore unraveling the Na^+^-bound state.

Biophysical and biochemical studies suggest that Na^+^ competes with H^+^ for a single binding site, and that Asp164 is the first proton carrier^2,19,22,54,59,68^. The salt-bridge model, supported by structural and mutagenesis data, proposes that Na^+^ binding displaces a proton from Asp164, then disrupts the salt bridge between Asp163 and Lys300, releasing a second proton from Lys300, followed by Na^+^ binding both Asp163 and Asp164^22,59^. Supporting this model, mutation of the equivalent Lys305 in NapA eliminates electrogenicity^69^, and the absence of Lys300 in electroneutral NHA2 implies its role in proton transfer^32^. A phylogenetic study further links electrogenic transporters to an Asp–Lys pair in the active site^50^. The alternative two-aspartate model proposes that both Asp163 and Asp164 act as proton carriers through p*K*_a_ shifts, without requiring proton release from Lys300^50,70,71^. Moreover, previous fixed-charge MD simulations with Asp163, Asp164 and Lys300 all deprotonated (i.e., corresponding to the salt-bridge model) show instances of two Na^+^ ions binding, which may not be physiologically relevant^35^. Notably, the electrogenic transport was maintained for NhaA when Lys300 alone or both Asp163 and Lys300 were mutated to an uncharged residue, albeit with 40–100× reduction in activity^70,71^.

Our cryo-EM structures and cpH-MD simulations offer refinements to the model for the Na^+^/H^+^ exchange by NhaA. In the Na^+^-bound state, the simulation shows deprotonation of Asp164 and an increase in the Asp163–Lys300 distance. However, Asp163 begins to deprotonate only at alkaline pH, and Lys300 is always protonated across all pH values (Fig. 3j, Supplementary Fig. 15). These results indicate that Asp163–Lys300 might form a hydrogen bond instead of a salt bridge. Such an arrangement could preserve conformational coupling while allowing flexibility in proton transfer. Furthermore, deprotonation of Asp163 upon Na^+^ binding supports the two-aspartate model, in which Asp163 and Asp164 undergo p*K*_a_ shifts and act as proton carriers. While our cpH-MD implementation is not formally validated for very large p*K*_a_ shifts, the algorithm closely resembles that of Harris et al^72^.: both of them are explicit, all-atom cpH-MD implementations using charge interpolation with Particle-Mesh-Ewald (PME), with the latter being able to capture the large p*K*_a_ shift of Asp26 in thioredoxin. Although whether Asp163 or Lys300 is the second proton carrier remains to be fully resolved, our results align with earlier studies proposing that Asp163 or Asp133 may act as proton carriers when Lys300 is mutated, preserving electrogenicity^70,73^.

In terms of conformational transitions, our data suggest a mini-elevator-bundle mechanism, a hybrid of the rocking-bundle and elevator models. Previous studies proposed a rocking-bundle transition in NhaA, possibly with limited elevator-like displacement^74–77^. Our cryo-EM structures show that TM V, together with the ion-binding site, moves 2 Å perpendicular to the membrane towards the cytoplasm upon Na^+^ binding. This is a modest movement compared to a canonical elevator movement (~10–18 Å per transport cycle)^76^, but sufficient to support an alternating access mechanism. This limited movement, combined with angular shifts in TMs, may allow NhaA to achieve high turnover while maintaining tight control of ion access and coupling^53,77^.

**Fig. 6:**
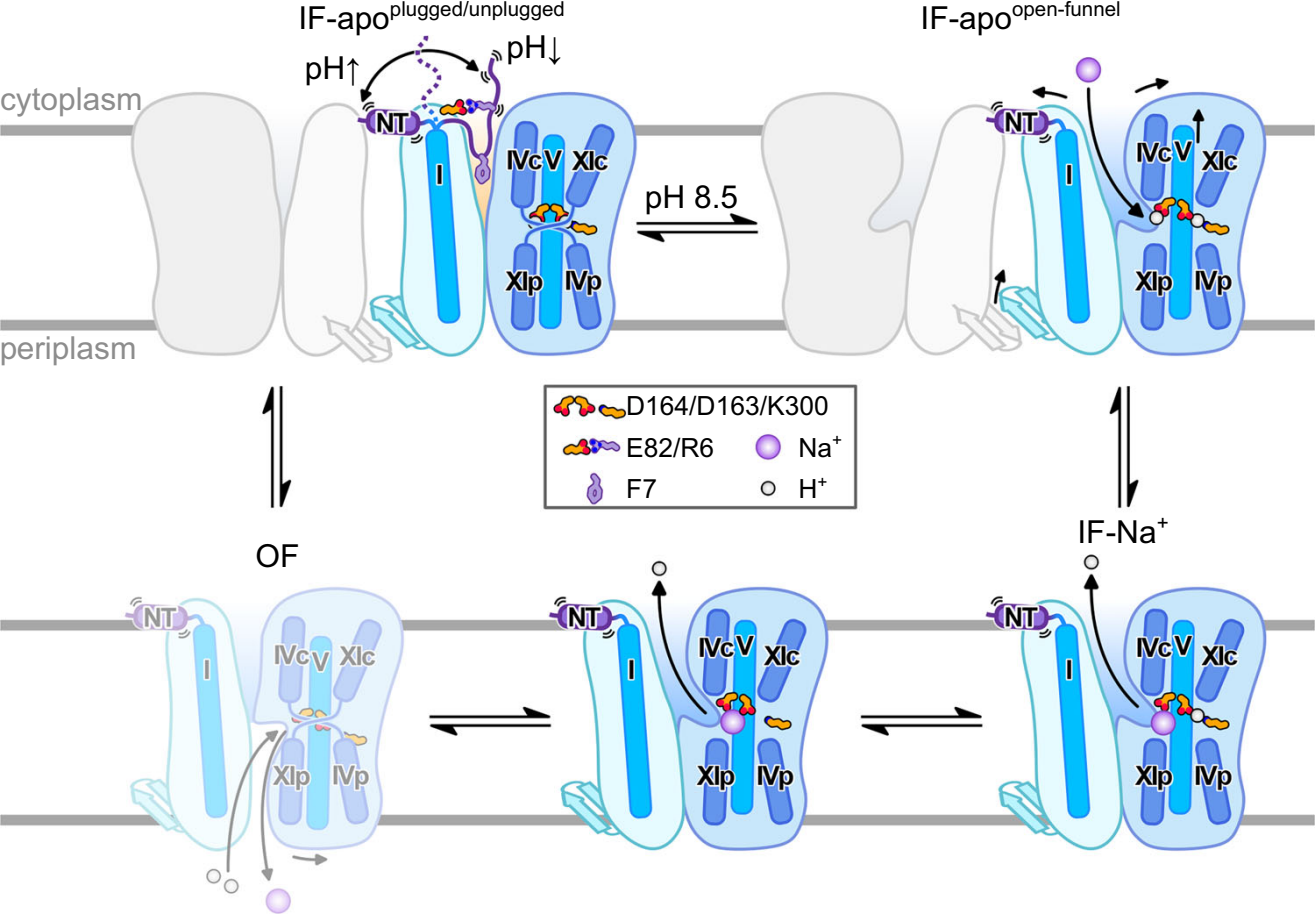
Schematic illustration of NhaA transport cycle. The second protomer (gray) is only shown for the inward-facing apo states to highlight the allosteric conformational change. For clarity, the unwound loops in TMs IV and XI are hidden in the IF-apo^open-funnel^ and IF-Na^+^ states to clearly show the ion-binding site. The OF conformation is hypothesized based on known structures of NhaA fold transporters.

Taken together, our cryo-EM structures and MD simulations define a sequential mechanism for NhaA electrogenic transport (Fig. 6). At low pH, NhaA exists in a conformational equilibrium between the plugged and unplugged IF states. As pH rises, rearrangement of the pH sensor promotes NT unplugging and progressive funnel opening. Consequently, the cytoplasmic funnel becomes fully open at pH 8.5. Na^+^ enters the cytoplasmic funnel, binds Asp164 (displacing one proton), and disrupts the Asp163–Lys300 interaction (releasing a second proton). This transition initiates the conformational switch toward the outward-facing state. Re-protonation and Na^+^ release complete the cycle, allowing the transporter to reset.

In summary, our study elucidates how pH sensing, N-terminal gating, and substrate recognition are integrated in NhaA. The use of cryo-EM with a native-like lipid environment enabled resolution of elusive structural elements and captured key intermediate states along the activation trajectory. These insights revise current models of Na^+^/H^+^ exchange and establish a framework for understanding other electrogenic antiporters, with potential implications for the design of antimicrobial strategies targeting bacterial ion homeostasis.

## Methods

### Generation of N-terminal deletion NhaA construct

Deletion of the first 13 amino acids at the N-terminus of *nhaA* was carried out by polymerase chain reaction using the bacterial expression plasmid pAXH3, which carries WT *nhaA*, as the template^78^. Two linear fragments of the plasmid were amplified using primer pairs F2del13AA/R1del13AA and F1del13AA/R2del13AA (Supplementary Table 4). The design deleted residues 1–13, added a methionine residue as a new initiation codon, and incorporated overhangs on both fragments for downstream assembly. The resulting PCR fragments were assembled using Gibson Assembly Master Mix (NEB). The assembled plasmid (pAXH3MΔ1–13, hereafter referred to as pΔNT) was transformed into XL10-Gold cells, sequenced for verification, and propagated. Plasmid DNA was purified using the FB Plasmid Miniprep Kit (FairBiotech).

### Production and purification of wild-type NhaA

Overexpression and affinity purification of the C-terminally His-tagged WT NhaA were performed as described^19^. In brief, *E. coli* strain RK20 cells transformed with pAXH3 harboring His-tagged NhaA and pI^Q^ (a pACYC184 derivative) encoding LacI^Q^ were grown in minimal medium supplemented with 6.5 μg/ml tetracycline and 100 μg/ml carbenicillin until 0.8 OD_600_ followed by induction with 1 mM isopropyl β-D-1-thiogalactopyranoside (IPTG). After 1.5 h, the cells were harvested, washed with TSC buffer (10 mM Tris pH 7.5, 250 mM sucrose, and 150 mM choline chloride), and resuspended in the same buffer with additional 1 mM dithiothreitol, 1 mM aminocaproic acid, 1 mM benzamidine, 2.5 mM MgCl_2_, 0.05 mg/ml DNase I (Roche), and 0.1 g/ml SigmaFast ethylenediaminetetraacetic acid (EDTA)-free protease inhibitor cocktail (Sigma-Aldrich). The cells were disrupted using a microfluidizer (Microfluidics), and the lysate was centrifuged with a Sorvall GSA rotor at 24,000 *g*, 4 °C for 45 min. Subsequently, the supernatant was centrifuged with a Ti45 rotor at 220,000 *g*, 4 °C for 2 h. Pelleted membranes were resuspended in TSC buffer, flash-frozen in liquid nitrogen, and stored at −80 °C.

Isolated membranes were thawed, solubilized in solubilization buffer (100 mM MOPS-KOH pH 7.0, 25% (v/v) glycerol, 1% (w/v) n-dodecyl-β-D-maltoside (β-DDM)), and incubated on a rotary wheel at 4 °C for 1 h. The insoluble membrane fraction was removed by centrifuging with a Ti70 rotor at 210,000 *g*, 4 °C for 1 h. The supernatant was subjected to Ni-NTA resin pre-equilibrated with binding buffer (20 mM Tris pH 8.0, 500 mM KCl (analytical grade), 5 mM imidazole pH 7.9, and 0.03% β-DDM). The resin was first washed with 10 column volume (CV) binding buffer, 10 CV wash buffer (20 mM Tris pH 8.0, 500 mM KCl, 30 mM imidazole pH 7.9, and 0.03% β-DDM) and eluted with 4 CV elution buffer (20 mM Tris pH 8.0, 500 mM KCl, 300 mM imidazole pH 7.9, and 0.03% β-DDM). Subsequently, the eluate was subjected to size-exclusion chromatography using a Superdex Increase 200 10/300 column (Cytiva) equilibrated in 20 mM HEPES-KOH pH 8.0, 100 mM KCl, and 0.02% β-DDM. The peak fractions were pooled for nanodisc reconstitution.

### Production and purification of Fv6F9

The DNA sequences encoding the Fv fragment of the monoclonal antibody 6F9^40^ was cloned into the plasmid pASK68^79^. Overexpression and affinity purification of the strep-tagged Fv6F9 was performed as previously described^79^. In brief, *E. coli* strain JM83 cells transformed with pASK68-Fv6F9 were grown in Luria broth (LB) containing 50 μg/ml ampicillin at 22.5 °C until 0.5 OD_600_, followed by induction with 1 mM IPTG. After 2.5 h, the cells were harvested, resuspended in ice-cold periplasm extraction buffer (50 mM Tris pH 8.0, 500 mM sucrose, and 1 mM EDTA, 0.2 mg/ml lysozyme), and incubated without stirring for 30 min. Spheroplasts were removed by centrifugation with a Sorvall GS-3 rotor at 12,000 *g*, 4 °C for 30 min. The resulting periplasm extract was incubated at 4 °C for 30 min with additional 8 mM MgCl_2_, 40 μg/ml avidin, and 0.05 mg/ml DNase I, flash-frozen in liquid nitrogen, and stored at −80 °C.

To purify Fv6F9, the periplasm extract was thawed and loaded onto Strep-Tactin XT 4Flow (IBA Lifesciences) resin pre-equilibrated with equilibration buffer containing 50 mM Tris pH 8.0, 100 mM KCl, 1 mM EDTA, washed with 10 CV equilibration buffer, and eluted with 6 CV equilibration buffer containing 50 mM biotin. The eluted Fv6F9 was concentrated using an Amicon 10 kDa cut-off concentrator (Millipore), flash-frozen in liquid nitrogen, and stored at −80 °C for future use.

### Cell growth phenotype assay

Cells were grown either in LB or in modified LB (LBK), in which NaCl was replaced with KCl. The medium was buffered with 60 mM 1,3-bis[tris(hydroxymethyl)methylamino]propane (BTP). For plates, 1.5% (w/v) agar was used. To test resistance to Li^+^ or Na^+^, EP432 cells^62^ transformed with the respective plasmids were grown in LBK to an OD_600_ of 0.5. Samples (2 μl) of serial 10-fold dilutions of the cultures were spotted onto agar plates containing the selective media: modified LB in which NaCl was replaced with the indicated concentrations of NaCl or LiCl at the various pH values and incubated for 2–3 days at 37 °C. pBR322 was used as a negative control.

### Detection and quantitation of NhaA and its variants in the membrane

Total membrane protein was determined according to Bradford^80^. The expression level of His-tagged NhaA mutant was determined by resolving the Ni-NTA-purified proteins by SDS-PAGE, staining the gels with Coomassie Blue, and quantifying the band densities by Image Gauge software (Fujifilm).

### Isolation of everted membrane vesicles and assay of Na^+^/H^+^ antiport activity

Everted membrane vesicles were isolated from EP432 cells expressing WT-NhaA or the indicated variants grown in LBK (pH 7.0) as described^63,81^. The ΔpH across the membranes was monitored using acridine orange, a fluorescence probe of ΔpH. The reaction mixture (2.5 ml) contained 50–100 µg of membrane protein, 0.5 µM acridine orange, 150 mM KCl, 50 mM BTP, 5 mM MgCl_2_, and the pH was titrated with HCl as indicated in Supplementary Fig. 19. The fluorescence of acridine orange was measured using a PerkinElmer LS-45 fluorescence spectrometer with an excitation wavelength of 490 nm and an emission wavelength of 530 nm. At the onset of the reaction, 2 mM D-lactate was added, and the fluorescence quenching was recorded until a steady-state level of ΔpH (100 % quenching) was reached. NaCl (10 mM) was then added, and the new steady state of the fluorescence (dequenching) was monitored. Fluorescence dequenching indicated that protons are exiting the vesicles in response to Na^+^ influx via the antiporter. All experiments were repeated at least three times. In the *K*_m_ measurement for ΔNT-NhaA at pH 8.5, NaCl was added as indicated in Supplementary Fig. 19b for each data point. The *K*_m_ of ΔNT-NhaA was acquired by fitting the Michaelis-Menten equation using python.

### Nanodisc reconstitution of NhaA-Fv6F9

The *E. coli* polar lipid extract (Avanti) dissolved in chloroform at 25 mg/ml was dried under nitrogen gas overnight. The dried lipid was resuspended in buffer containing 20 mM Tris pH 7.5, 150 mM KCl, and 200 mM sodium cholate to a final concentration of 78.6 mg/ml, and sonicated in a water bath sonicator to transparency. Nanodisc reconstitution was performed by mixing purified NhaA, MSP1D1, and lipids at a molar ratio of 1:10:600 with a final sodium cholate concentration adjusted to 25 mM, followed by incubation at 4 °C for 1 h. The detergent was then removed by adding 0.04 g/ml activated SM-2 Bio-Beads (Bio-Rad) and incubated on a rotary wheel at 4 °C for 1 h, then 0.04 g/ml SM-2 Bio-Beads followed by incubation at 4 °C for 1 h, and finally 0.06 g/ml SM-2 Bio-Beads followed by incubation at 4 °C overnight. Subsequently, the reconstituted nanodiscs were filtered and loaded onto a Superdex Increase 200 10/300 column equilibrated with 20 mM BTP pH 8.5, and 100 mM KCl. Peak fractions of NhaA-containing nanodiscs were pooled, mixed with purified Fv6F9 in a molar ratio of 1:1.2, and loaded onto a Superdex Increase 200 10/300 column equilibrated with 20 mM BTP pH 8.5, and 100 mM KCl. Peak fractions for size-exclusion chromatography were pooled for single-particle cryo-EM analysis.

### Cryo-EM sample preparation

The reconstituted NhaA-Fv6F9 complex in nanodiscs was buffer-exchanged to different conditions indicated in Supplementary Table 2 using Amicon 50 kDa cut-off concentrators and concentrated to 1.5 mg/ml for cryo-EM sample preparation. Quantifoil R1.2/1.3 300 mesh copper grids were washed in chloroform and glow-discharged with a PELCO easiGlow device at 15 mA for 90 s. A volume of 4 μl sample was applied to a glow-discharged grid, blotted for 4 s with a nominal blot force of 20, and plunge-frozen in liquid ethane using a Vitrobot Mark IV (Thermo Scientific).

### Cryo-EM data collection

Each cryo-EM dataset was recorded in energy-filtered transmission electron microscopy mode using a Titan Krios G3i microscope (Thermo Scientific) operated at 300 kV. Electron-optical alignment was performed with EPU 3.7–3.9 (Thermo Scientific). Images were recorded in electron-counting mode using a Gatan K3 direct electron detector with a nominal magnification of 105,000, corresponding to a calibrated pixel size of 0.837 Å, and with a nominal defocus between −1.1 and −2.1 μm. Dose fractionated movies of 50 frames were recorded at an electron dose rate of about 15 e^−^/pixel/s for 3.2 s, corresponding to a total dose of approximately 50 e^−^/Å^2^.

### Cryo-EM image processing

Each cryo-EM dataset was processed using the same approach: Beam-induced motion correction and dose-weighted image generation were done using MotionCor2 implemented in RELION 4^82,83^. CtfFind was used to estimate contrast transfer function (CTF) parameters^84^. Images with estimated resolution over 4.0 or 4.5 Å and astigmatism over 400 Å were discarded. Particles were picked with TOPAZ for further processing^85^. Two-dimensional classification, initial model generation, three-dimensional (3D) classification, 3D refinement, Bayesian polishing, CTF refinement, and final map reconstruction with map sharpening B-factors shown in Supplementary Table 2 were done using RELION 4^83^. Fourier shell correlation (FSC) curves and local resolution estimation were done in RELION 4 for all final maps. An overview of the data processing workflow and a summary of map qualities are shown in Supplementary Figs. 2 and 3, respectively.

Assuming no allosteric coupling between protomers, the theoretical (non-allosteric) ratios of NhaA dimer states (Fig. 2g) were calculated from the fractional populations of individual protomer states (${P}_{{state}}$; Fig. 2f) as all pairwise products of the four possible states, corresponding to the expansion of

$${\left({P}_{{plugged}}+{P}_{{flexNT}}+{P}_{{unplugged}}+{P}_{{open}-{funnel}}\right)}^{2}$$

This yields both homodimeric (e.g., ${{P}_{{plugged}}}^{2}$) and heterodimeric (e.g., ${2P}_{{plugged}}\times {P}_{{unplugged}}$) combinations expected for independent protomers.

### Model building and geometry refinement

The first atomic model was built into the cryo-EM density map of the IF-apo NhaA-Fv6F9 complex at pH 7.5 using ISOLDE within ChimeraX and Coot^86–88^, based on the crystal structure of NhaA (PDB 4AU5)^22^ and the Fv6F9 model generated by ModelAngelo^61^, followed by real space refinement in Phenix^89,90^. The refined model was manually inspected and adjusted if necessary. The model of IF-apo NhaA-Fv6F9 at pH 7.5 was used as a template to build the other atomic models. All the models were validated by MolProbity implemented in Phenix^91^. Map-to-model cross-validation was performed in Phenix^89^. Comprehensive information on the cryo-EM data collection, refinement, and validation statistics is shown in Supplementary Table 2. ChimeraX was used to visualize the structures shown in this study.

### Molecular dynamics simulations

Explicit, all-atom constant-pH molecular dynamics (cpH-MD) simulations^39^ were performed starting from the monomeric form of a previously determined crystal structure at pH 3.5 (PDB 4AU5)^22^, the IF-Na^+^ structure without the NT, as well as the IF-apo^plugged^ and IF-apo^unplugged^ structures (Supplementary Table 5). The protein structures were oriented in the membrane using the OPM webserver^92^ and, following the previous simulation studies^59,73^, inserted into a 1-palmitoyl-2-oleoyl-sn-glycero-3-phosphocholine (POPC) membrane using the Membrane Builder module of CHARMM-GUI^93^. The membrane consisted of 198 or 199 lipids, depending on the starting structure, and was surrounded by a 30 Å-thick water layer on both sides, providing a 60 Å separation between periodic images. Sodium and chloride ions were added to neutralize the system at a salt concentration of ~150 mM in the aqueous phase, resulting in systems comprising approximately 80,000 atoms. We equilibrated the systems following the CHARMM-GUI protocol. The equilibrated systems were prepared for cpH-MD by phbuilder (v1.3)^94^ using 48 buffer particles and simulated in three replicas between pH 4.0 and 9.5 in pH increments of 0.5 (initial structures 4AU5 and IF-Na^+^ without the NT), and at pH 4.0, 8.5, 9.5 (initial structures IF-apo^plugged^ and IF-apo^unplugged^) using the Particle-Mesh-Ewald-enabled^95^ continuous cpH-MD code^39^ implemented in the GROMACS package (v2021)^96^. The cpH-MD adapted version^94^ of the CHARMM36m^97^ force field was used to represent the protein, and the CHARMM TIP3P^98^ model was used for water molecules. During the simulations, all Asp, Glu, His, Lys, and Arg residues were allowed to titrate. All cpH-MD-related simulation parameter were adopted from Aho et al^39^.. In particular, the mass of the λ-particle was 5 a.u. and the coupling time was set to 2 ps. After successive energy minimization and 4 ns of restrained simulations in the NVT and NPT ensembles, 2 μs-long trajectories were simulated with a timestep of 2 fs. The temperature was kept at 300 K using a velocity-rescaling thermostat, while a pressure of 1 bar was maintained using a c-rescale barostat^99^. For constant-pH-related options, the default were used^39,94^. All calculations were repeated with a layer-like flat-bottomed position restraint (*k* = 200 kJ/mol/nm^−2^) applied to all Na^+^ ions to block them from entering the active site, and simulated for 1 μs. Convergence was assessed based on the RMSD of the C_α_ atoms involved in the TMs and on the protonation state of the titratable residues, shown in Supplementary Figs. 11 and 13. The first 200 ns of these trajectories was discarded as equilibration, and the rest was analyzed using in-house scripts written using the MDAnalysis package (v2.10.0)^100^. Discarding the first 500 ns instead of 200 ns did not affect the extracted p*K*_a_ values, which are collected in Supplementary Table 3. While the behavior of titratable residues appears to be converged, the C_α_ RMSD shows conformational dynamics at the highest pH beyond 200 ns. Although clarifying these motions would be highly desirable, we were limited by the current performance of our cpH-MD implementation.

### Multiple sequence alignment

The multiple protein sequence alignment of enterobacterial NhaA was performed using Clustal Omega^101^. The alignment result was used to generate the sequence logo using WebLogo^102^.

### Reporting summary

Further information on research design is available in the Nature Portfolio Reporting Summary linked to this article.

## Supplementary information

Supplementary Information

Description of Additional Supplementary Files

Supplementary Movie 1

Reporting Summary

Transparent Peer Review file

## Source data

Source Data

## Data Availability

Cryo-EM maps and atomic models of *E. coli* NhaA have been deposited in the Electron Microscopy Data Bank and Protein Data Bank under accession codes: EMD-53954 and PDB 9RH1 10.2210/pdb9rh1/pdb (IF-apo consensus dimer (pH 7.5)); EMD-53955 and PDB 9RH2 10.2210/pdb9rh2/pdb (IF-apo^unplugged^ (pH 7.5)); EMD-53956 and PDB 9RH3 10.2210/pdb9rh3/pdb (IF-apo^plugged^ (pH 7.5)); EMD-53957 and PDB 9RH4 10.2210/pdb9rh4/pdb (IF-apo^flexNT^ (pH 7.5)); EMD-53958 and PDB 9RH5 10.2210/pdb9rh5/pdb (IF-apo^unplugged^ (pH 6.3)); EMD-53959 and PDB 9RH6 10.2210/pdb9rh6/pdb (IF-apo^plugged^ (pH 6.3)); EMD-53960 and PDB 9RH7 10.2210/pdb9rh7/pdb (IF-apo^flexNT^ (pH 6.3)); EMD-53961 and PDB 9RH8 10.2210/pdb9rh8/pdb (IF-apo^unplugged^ (pH 5.5)); EMD-53962 and PDB 9RH9 10.2210/pdb9rh9/pdb (IF-apo^plugged^ (pH 5.5)); EMD-53963 and PDB 9RHA 10.2210/pdb9rha/pdb (IF-apo^flexNT^ (pH 5.5)); EMD-53964 and PDB 9RHB 10.2210/pdb9rhb/pdb (IF-apo^open-funnel^ (pH 8.5)); EMD-53965 and PDB 9RHC 10.2210/pdb9rhc/pdb (IF-apo^unplugged^ (pH 8.5)); EMD-53966 and PDB 9RHD 10.2210/pdb9rhd/pdb (IF-apo^plugged^ (pH 8.5)); EMD-53967 and PDB 9RHE 10.2210/pdb9rhe/pdb (IF-apo^flexNT^ (pH 8.5)); EMD-53968 and PDB 9RHF 10.2210/pdb9rhf/pdb (IF-Na^+^ (pH 8.5)). All molecular dynamics simulation input files and final outputs are available on Zenodo (DOI: 10.5281/zenodo.19354200 10.5281/zenodo.19354200. All other data are presented in the main text or supplementary materials. Source data are provided with this paper. Several previously published PDB codes are referred in this study: PDB 4AU5 10.2210/pdb4au5/pdb; PDB 7S24 10.2210/pdb7s24/pdb; PDB 7A0W 10.2210/pdb7a0w/pdb; PDB 8PS0 10.2210/pdb8ps0/pdb. Source data are provided with this paper.
