# Supplementary material for: pH-dependent activation of the Na+/H+ antiporter NhaA and conformational dynamics of its N-terminus
Source: Nat Commun. 2026 Jun 12;17:7489. doi: 10.1038/s41467-026-73424-2 (PMC13408996; doi:10.1038/s41467-026-73424-2)
Supplement: Supplementary file 1 — Supplementary Information [file 41467_2026_73424_MOESM1_ESM.pdf]

pH-dependent activation of the Na^{+}/H^{+} antiporter NhaA and conformational dynamics of its N-terminus

Tsai-Hsuan Weng[1†], Balazs Fabian[2†], Elena Olkhova[2], Sonja Welsch[3], Sarah Luise Schmidt[1‡], Tsafi Danieli[4], Yael Keren[4], Abraham Rimon[5], Schara Safarian[1,6–8], Gerhard Hummer[2,9], Etana Padan[5*], Hartmut Michel[1*]

Affiliations

[1]Emeritus Group Molecular Membrane Biology, Max Planck Institute of Biophysics, Frankfurt, Germany

[2]Department of Theoretical Biophysics, Max Planck Institute of Biophysics, Frankfurt, Germany

[3]Central Electron Microscopy Facility, Max Planck Institute of Biophysics, Frankfurt, Germany

[4]The Protein Production Facility, Alexander Silberman Institute of Life Sciences, the Hebrew University of Jerusalem, Jerusalem, Israel

[5]Department of Biological Chemistry, Alexander Silberman Institute of Life Sciences, the Hebrew University of Jerusalem, Jerusalem, Israel

[6]Fraunhofer Institute for Translational Medicine and Pharmacology ITMP Frankfurt, Frankfurt, Germany

[7]Fraunhofer Cluster of Excellence for Immune Mediated Diseases CIMD, Frankfurt, Germany

[8]Institute of Clinical Pharmacology, Goethe University Frankfurt, Frankfurt, Germany

[9]Institute of Biophysics, Goethe University Frankfurt, Frankfurt, Germany

[*]Correspondence and requests for materials should be addressed to Etana Padan (etana.padan@mail.huji.ac.il), and Hartmut Michel (hartmut.michel@biophys.mpg.de).

[†]These authors contributed equally.

[‡]Current address: Center for Life Sciences, Paul Scherrer Institute, Villigen PSI, Switzerland

This file includes:

Supplementary Tables 1–5

Supplementary Figs. 1–21

Supplementary Table 1: Buffer conditions for the NhaA cryo-EM samples

Sample	Buffer
pH 5.5-K^+	20 mM MES[a] pH 5.5, 300 mM KCl
pH 6.3-K^+	20 mM BTP[b] pH 6.3, 300 mM KCl
pH 7.5-K^+	20 mM BTP pH 7.5, 300 mM KCl
pH 8.5-K^+	20 mM BTP pH 8.5, 300 mM KCl
pH 8.5-Na^+	20 mM BTP pH 8.5, 300 mM NaCl

[a] 2-(*N*-morpholino)ethanesulfonic acid

[b] 1,3-bis[tris(hydroxymethyl)methylamino]propane

Supplementary Table 2: NhaA cryo-EM and model data statistics.

	IF-apo consensus dimer (pH 7.5) (EMD-53954; PDB 9RH1)	IF-apo^{unplugged} (pH 7.5) (EMD-53955; PDB 9RH2)	IF-apo^{plugged} (pH 7.5) (EMD-53956; PDB 9RH3)	IF-apo^{flexNT} (pH 7.5) (EMD-53957; PDB 9RH4)	IF-apo^{unplugged} (pH 6.3) (EMD-53958; PDB 9RH5)
**Data collection and processing**					
Magnification	105,000	105,000	105,000	105,000	105,000
Voltage (kV)	300	300	300	300	300
Electron dose (e^{-}/Å^{2})	50	50	50	50	50
Defocus range (µm)	−1.1 to −2.1	−1.1 to −2.1	−1.1 to −2.1	−1.1 to −2.1	−1.1 to −2.1
Pixel size (Å)	0.837	0.837	0.837	0.837	0.837
Symmetry imposed	*C*2	*C*1	*C*1	*C*1	*C*1
Initial particle images (no.)	5,317,495	5,317,495	5,317,495	5,317,495	11,414,713
Final particle images (no.)	144,462	127,302	131,498	30,124	87,709
Map resolution (Å)	2.7	2.7	2.7	3.1	3.3
FSC threshold	0.143	0.143	0.143	0.143	0.143
Map resolution range (Å)	2.6–4.2	2.6–4.2	2.6–4.2	2.9–5.0	3.3–4.9
**Refinement**					
Initial model used	PDB 4AU5 (NhaA) ModelAngelo generated (Fv6F9)	PDB 9RH1	PDB 9RH1	PDB 9RH1	PDB 9RH1
Model resolution (Å)	2.7	2.7	2.7	3.1	3.5
FSC threshold	0.5	0.5	0.5	0.5	0.5
Model resolution range (Å)					
Map sharpening *B* factor (Å^{2})	−40	−50	−50	−50	−90
Model composition					
Non-hydrogen atoms	9224	4643	4643	4553	4626
Protein residues	1206	612	612	602	610
Water	–	–	–	–	–
Ligands	CDL: 1<br>PGT: 1	–	–	–	–
Average *B* factors (Å^{2})					
Protein	86.02	85.00	88.65	74.63	65.05
Ligand	58.38	–	–	–	–
R.m.s. deviations					
Bond lengths (Å)	0.003	0.004	0.004	0.002	0.004
Bond angles (°)	0.660	0.636	0.581	0.527	0.802
Validation					
MolProbity score	1.35	1.29	1.31	1.20	1.45
Clashscore	6.31	5.42	5.21	4.12	8.23
Poor rotamers (%)	0.00	0.61	0.20	0.00	0.00
CαBLAM outliers (%)	1.52	1.67	1.33	1.69	1.67
Ramachandran plot					
Favored (%)	98.07	98.02	97.85	98.15	98.18
Allowed (%)	1.93	1.98	2.15	1.85	1.82
Disallowed (%)	0.00	0.00	0.00	0.00	0.00

Supplementary Table 2: (continued)

	IF-apo^{plugged} (pH 6.3) (EMD-53959; PDB 9RH6)	IF-apo^{flexNT} (pH 6.3) (EMD-53960; PDB 9RH7)	IF-apo^{unplugged} (pH 5.5) (EMD-53961; PDB 9RH8)	IF-apo^{plugged} (pH 5.5) (EMD-53962; PDB 9RH9)	IF-apo^{flexNT} (pH 5.5) (EMD-53963; PDB 9RHA)
**Data collection and processing**					
Magnification	105,000	105,000	105,000	105,000	105,000
Voltage (kV)	300	300	300	300	300
Electron dose (e$^-$/Å^2)	50	50	50	50	50
Defocus range (µm)	−1.1 to −2.1	−1.1 to −2.1	−1.1 to −2.1	−1.1 to −2.1	−1.1 to −2.1
Pixel size (Å)	0.837	0.837	0.837	0.837	0.837
Symmetry imposed	*C*1	*C*1	*C*1	*C*1	*C*1
Initial particle images (no.)	11,414,713	11,414,713	4,819,074	4,819,074	4,819,074
Final particle images (no.)	108,437	28,412	46,108	70,705	16,815
Map resolution (Å)	3.4	3.7	3.1	3.1	3.5
FSC threshold	0.143	0.143	0.143	0.143	0.143
Map resolution range (Å)	3.3–4.8	3.6–5.7	3.1–4.9	3.0–4.7	3.4–5.8
**Refinement**					
Initial model used	PDB 9RH1	PDB 9RH1	PDB 9RH1	PDB 9RH1	PDB 9RH1
Model resolution (Å)	3.5	3.8	3.3	3.1	3.6
FSC threshold	0.5	0.5	0.5	0.5	0.5
Model resolution range (Å)					
Map sharpening *B* factor (Å^2)	−80	−70	−60	−60	−50
Model composition					
Non-hydrogen atoms	4635	4553	4626	4635	4553
Protein residues	611	602	610	611	602
Water	–	–	–	–	–
Ligands	–	–	–	–	–
Average *B* factors (Å^2)					
Protein	89.89	79.85	116.18	108.80	80.65
Ligand	–	–	–	–	–
R.m.s. deviations					
Bond lengths (Å)	0.004	0.003	0.003	0.004	0.004
Bond angles (°)	0.694	0.578	0.568	0.578	0.556
Validation					
MolProbity score	1.58	1.41	1.24	1.28	1.41
Clashscore	7.89	7.48	4.70	3.94	4.88
Poor rotamers (%)	0.20	0.62	0.00	0.20	0.21
CαBLAM outliers (%)	1.84	1.53	1.17	1.50	2.03
Ramachandran plot					
Favored (%)	97.19	98.32	98.01	97.52	97.15
Allowed (%)	2.81	1.68	1.99	2.48	2.85
Disallowed (%)	0.00	0.00	0.00	0.00	0.00

Supplementary Table 2: (continued)

	IF-apo$^{open\text{-}funnel}$ (pH 8.5) (EMD-53964; PDB 9RHB)	IF-apo^{unplugged} (pH 8.5) (EMD-53965; PDB 9RHC)	IF-apo^{plugged} (pH 8.5) (EMD-53966; PDB 9RHD)	IF-apo^{flexNT} (pH 8.5) (EMD-53967; PDB 9RHE)	IF-Na^+ (pH 8.5) (EMD-53968; PDB 9RHF)
**Data collection and processing**					
Magnification	105,000	105,000	105,000	105,000	105,000
Voltage (kV)	300	300	300	300	300
Electron dose (e^-/Å^2)	50	50	50	50	50
Defocus range (μm)	−1.1 to −2.1	−1.1 to −2.1	−1.1 to −2.1	−1.1 to −2.1	−1.1 to −2.1
Pixel size (Å)	0.837	0.837	0.837	0.837	0.837
Symmetry imposed	*C*1	*C*1	*C*1	*C*1	*C*1
Initial particle images (no.)	7,971,548	7,971,548	7,971,548	7,971,548	5,657,422
Final particle images (no.)	149,520	92,700	79,098	18,232	173,768
Map resolution (Å)	2.9	3.2	3.2	3.5	3.0
FSC threshold	0.143	0.143	0.143	0.143	0.143
Map resolution range (Å)	2.8–4.3	3.0–4.9	3.0–5.0	3.4–6.2	2.8–4.2
**Refinement**					
Initial model used	PDB 9RH1	PDB 9RH1	PDB 9RH1	PDB 9RH1	PDB 9RH1
Model resolution (Å)	3.0	3.3	3.3	3.6	3.0
FSC threshold	0.5	0.5	0.5	0.5	0.5
Model resolution range (Å)					
Map sharpening *B* factor (Å^2)	−70	−70	−70	−70	−70
Model composition					
Non-hydrogen atoms	4644	4643	4587	4553	4646
Protein residues	612	612	606	602	612
Water	1	–	–	–	2
Ligands	–	–	–	–	NA: 1
Average *B* factors (Å^2)					
Protein	102.22	102.44	95.46	71.03	101.01
Ligand	–	–	–	–	70.74
R.m.s. deviations					
Bond lengths (Å)	0.004	0.005	0.003	0.002	0.003
Bond angles (°)	1.043	0.780	0.739	0.583	0.561
Validation					
MolProbity score	1.35	1.51	1.48	1.39	1.39
Clashscore	4.47	9.68	8.08	6.40	5.00
Poor rotamers (%)	0.20	0.00	0.41	0.21	0.20
CαBLAM outliers (%)	1.17	1.67	1.35	1.53	1.17
Ramachandran plot					
Favored (%)	97.36	98.02	97.83	97.82	97.36
Allowed (%)	2.64	1.98	2.17	2.18	2.64
Disallowed (%)	0.00	0.00	0.00	0.00	0.00

Supplementary Table 3: Estimated pK_a values from fitting the Hill equation with Hill coefficient n to the data from constant pH simulations. The Asp163 and Asp164 values were computed from simulations where Na^+ was blocked from entering the active site. For all other residues, the data was combined from all simulations. While titratable, all arginine residues were protonated over the entire simulated pH range.

**Residue**	**pK_a**	***n***
**pH Sensor**		
Asp11	<4.0	
Glu78	7.1 ± 0.4	0.8 ± 0.2
Glu82	<4.0	
His243	6.0 ± 0.1	1.1 ± 0.1
Lys249	>9.5	
Glu252	<4.0	
His253	5.7 ± 0.1	0.9 ± 0.1
His256	7.1 ± 0.1	0.9 ± 0.1
**Active site**		
Asp133	<4.0	
Asp163	>9.5	
Asp164	>9.5	
Lys300	>9.5	
**Others**		
His39	7.4 ± 0.1	0.9 ± 0.1
Asp40	4.1 ± 0.1	1.0 ± 0.1
Glu43	4.5 ± 0.1	0.8 ± 0.1
Glu54	4.0 ± 0.1	1.1 ± 0.1
Lys57	>9.5	
Asp65	5.9 ± 0.8	0.3 ± 0.2
Lys80	>9.5	
Asp119	4.7 ± 0.2	1.1 ± 0.1
Glu124	4.7 ± 0.3	1.0 ± 0.1
Lys153	>9.5	
Asp178	<4.0	
Lys221	>9.5	
His225	<4.0	
Lys240	>9.5	
Glu241	<4.0	
Lys242	>9.5	
Asp282	<4.0	
Lys315	>9.5	
His318	5.6 ± 0.1	0.9 ± 0.1
Glu321	<4.0	
Asp354	<4.0	
Glu356	<4.0	
Lys362	>9.5	

Supplementary Table 4: primers used in this study.

Primer	Sequence
F1del13AA	CTATTCACCTGAAAGAGAAATAAAAAatgGGAGGCATTATTCTTATCATTGCCGC
R2del13AA	GTAAACTTGGTCTGACAGTTACCAATGCTTAATCAGTG
F2del13AA	CTGTCAGACCAAGTTTACTCATATATAC
R1del13AA	TTTTTATTTCTCTTTCAGGTGAATAGATCG

Supplementary Table 5: system setup for the cpH-MD simulations performed in this study.

System / Structure	Box	Total atoms	Waters molecules	Salt	Lipids	pH	Replicas	Simulation time
4AU5 (monomer, pH 3.5 crystal structure)	$L_x \approx 9$ nm<br>$L_y \approx 9$ nm<br>$L_z \approx 10$ nm	~80,000	~15,000	150 mM NaCl	POPC: 198 or 199	4.0–9.5 (0.5 increment)	3 for each pH values	~2 µs
4AU5 (monomer, pH 3.5 crystal structure) *Na^+ blocked*	$L_x \approx 9$ nm<br>$L_y \approx 9$ nm<br>$L_z \approx 10$ nm	~80,000	~15,000	150 mM NaCl	POPC: 198 or 199	4.0–9.5 (0.5 increment)	3 for each pH values	~1 µs
IF-Na^+ (without NT)	$L_x \approx 9$ nm<br>$L_y \approx 9$ nm<br>$L_z \approx 10$ nm	~80,000	~15,000	150 mM NaCl	POPC: 198 or 199	4.0–9.5 (0.5 increment)	3 for each pH values	~2 µs
IF-Na^+ (without NT) *Na^+ blocked*	$L_x \approx 9$ nm<br>$L_y \approx 9$ nm<br>$L_z \approx 10$ nm	~80,000	~15,000	150 mM NaCl	POPC: 198 or 199	4.0–9.5 (0.5 increment)	3 for each pH values	~1 µs
IF-apo^{plugged}	$L_x \approx 9$ nm<br>$L_y \approx 9$ nm<br>$L_z \approx 10$ nm	~80,000	~15,000	150 mM NaCl	POPC: 198 or 199	4.0, 8.5, 9.5	3 for each pH values	~1 µs
IF-apo^{unplugged}	$L_x \approx 9$ nm<br>$L_y \approx 9$ nm<br>$L_z \approx 10$ nm	~80,000	~15,000	150 mM NaCl	POPC: 198 or 199	4.0, 8.5, 9.5	3 for each pH values	~1 µs

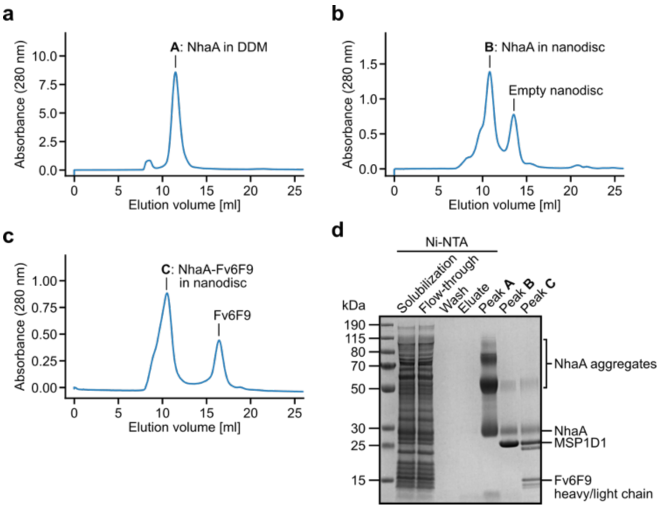

Supplementary Fig. 1: Purification and nanodisc reconstitution of NhaA. **a**–**c**, Size-exclusion chromatography profiles for NhaA in β-DDM after affinity purification (**a**), NhaA reconstituted into nanodisc (**b**) and NhaA-Fv6F9 in nanodisc (**c**). **d**, Coomassie-stained SDS-PAGE for NhaA purification and nanodisc reconstitution. Peaks A, B and C represent concentrated fractions from the main peaks marked in panels **a**–**c**. The high molecular weight bands of Peak A, B and C were aggregation of NhaA resulted from heating of the samples before SDS-PAGE. The uncropped gel is shown in Supplementary Fig. 21.

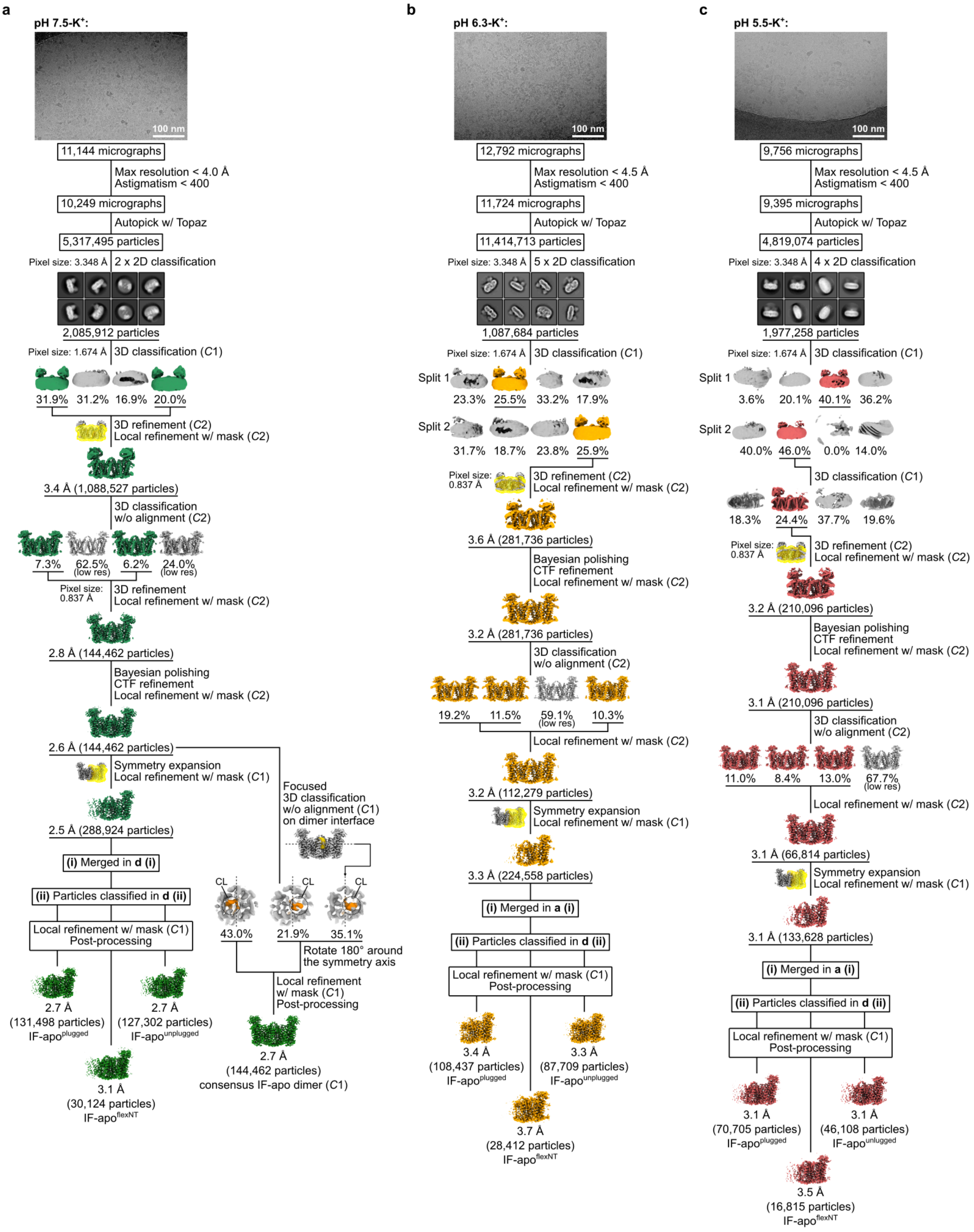

Supplementary Fig. 2: Single-particle cryo-EM analysis of NhaA under different conditions. **a**–**d**, NhaA, in the presence of K^+, at pH 8.5, 7.5, 6.3, and 5.5, respectively. **e**. NhaA at pH 8.5 in the presence of Na^+.

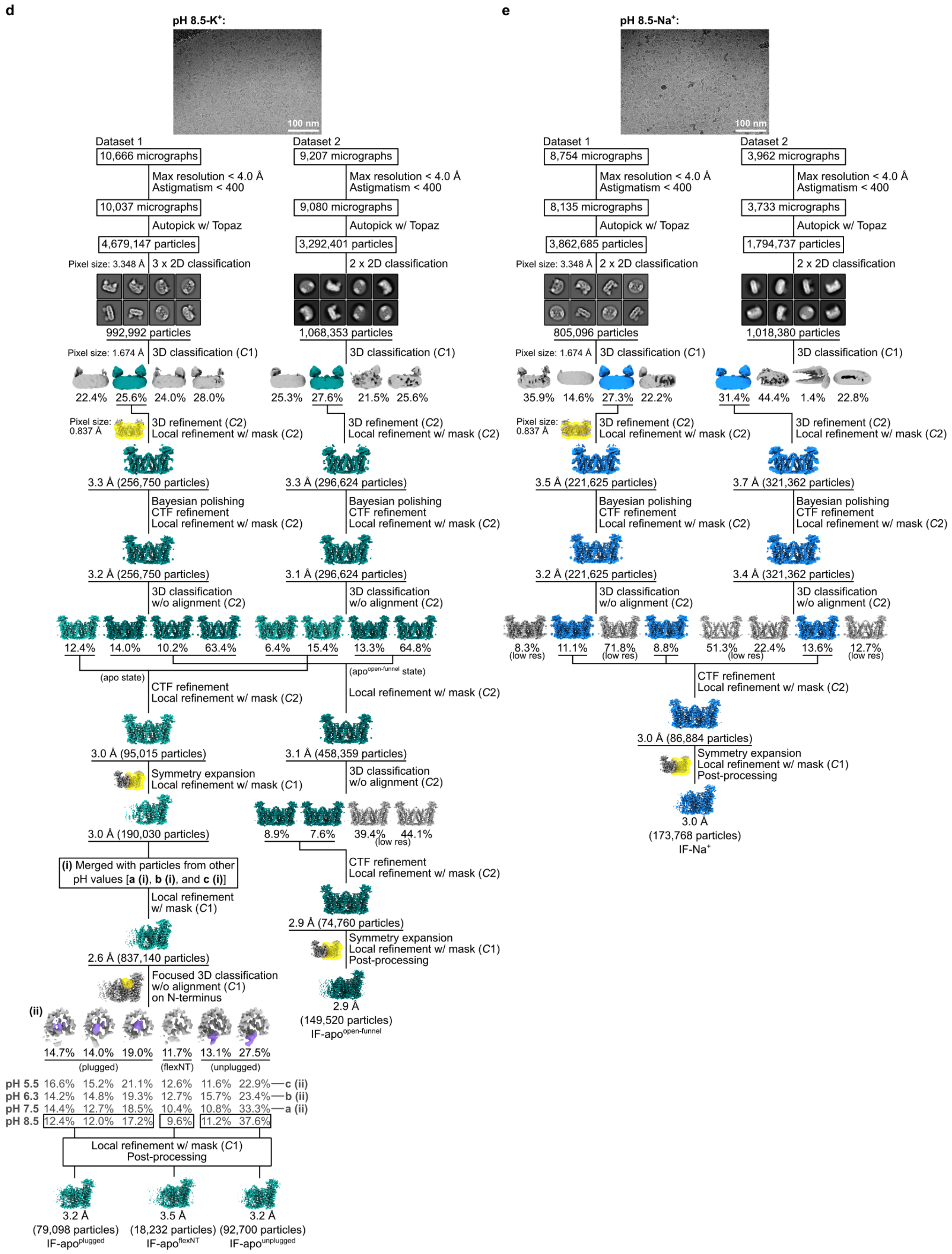

Supplementary Fig. 2: (continued)

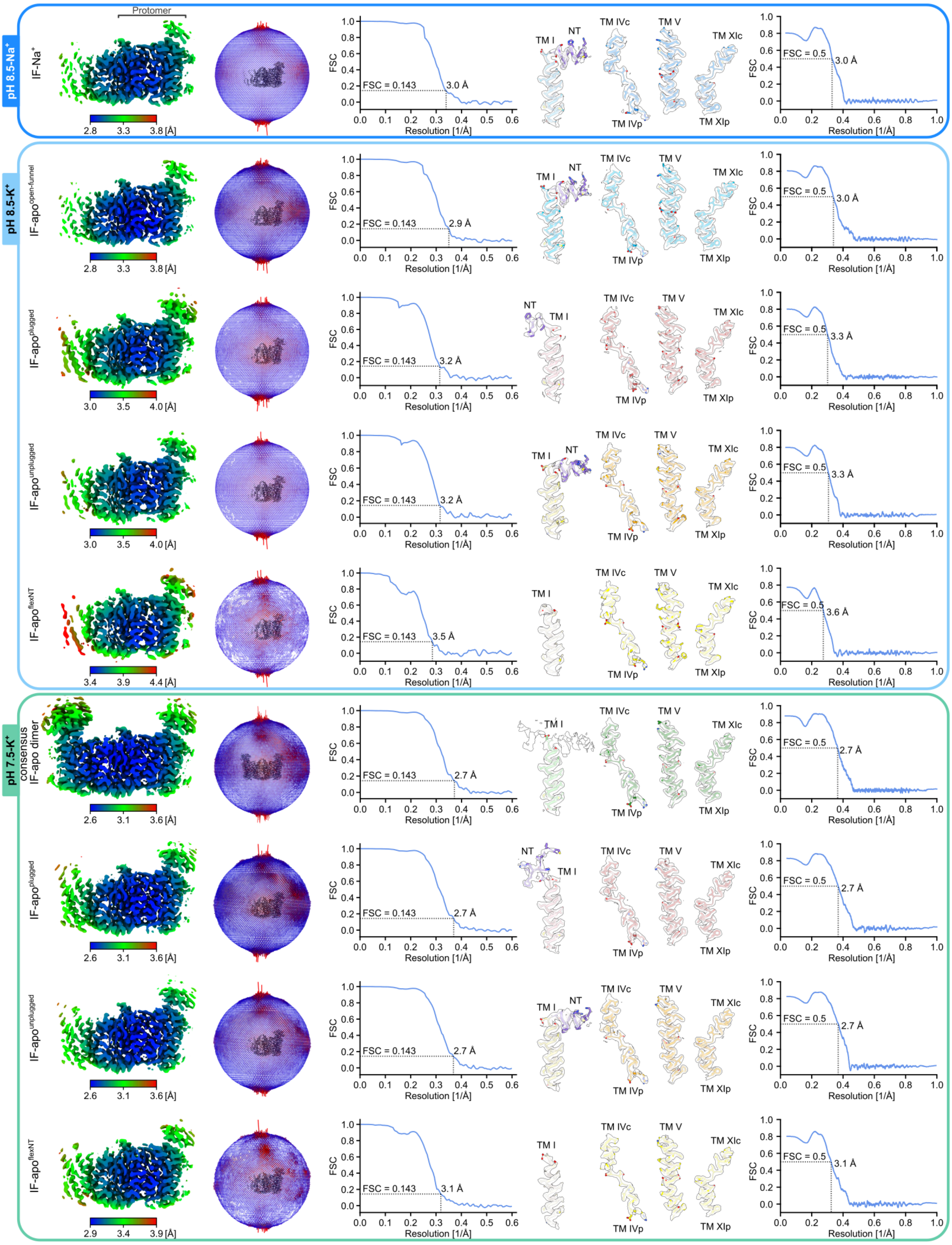

Supplementary Fig. 3: Local resolution estimation, angular distribution of particles, Fourier shell correlation (FSC) curves, representative cryo-EM maps of TMs, and cryo-EM map-to-model fitting FSCs of the final cryo-EM maps of NhaA under different conditions. The local resolution maps are shown as cut-away views exposing the ion-binding site.

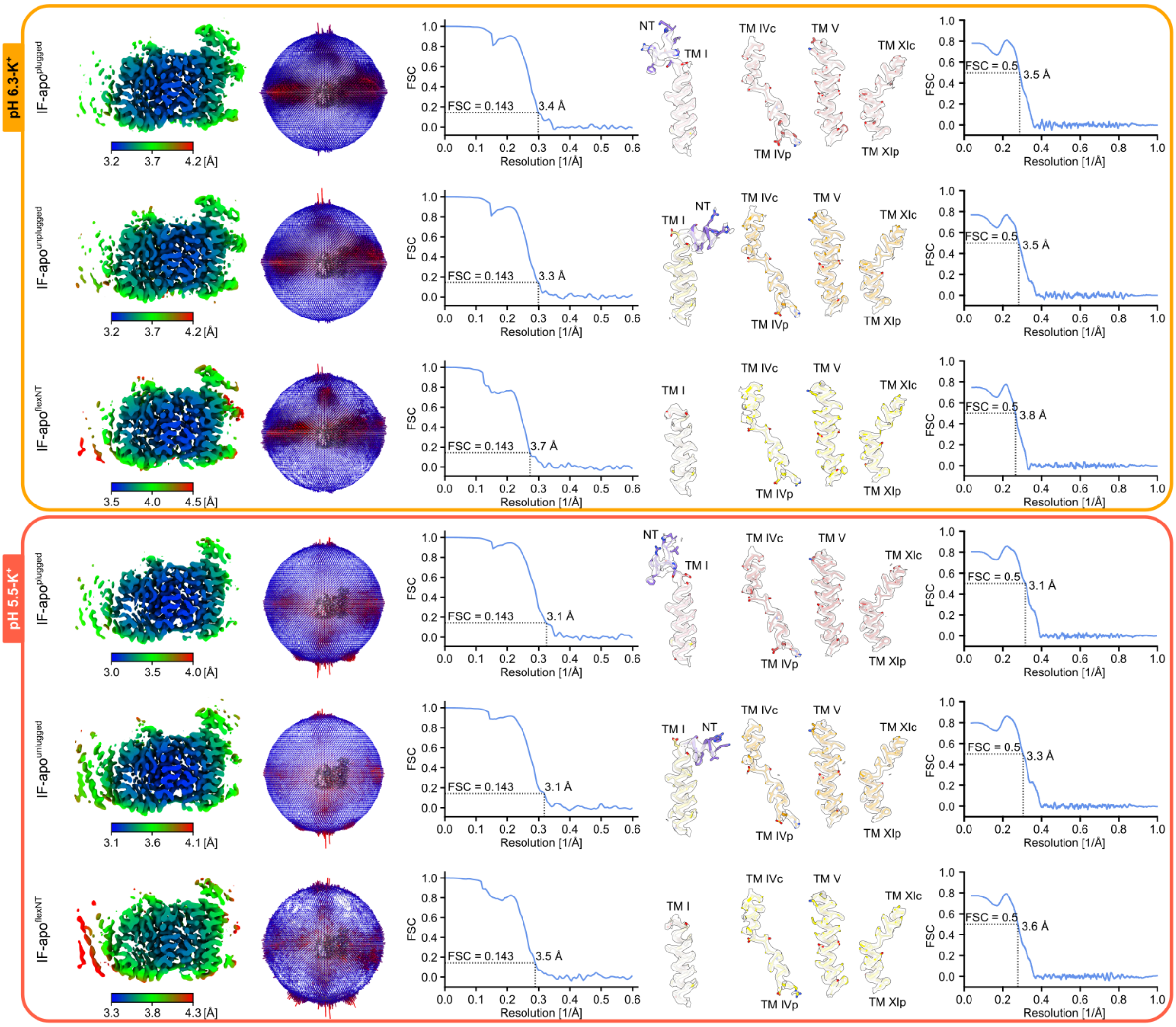

Supplementary Fig. 3: (continued)

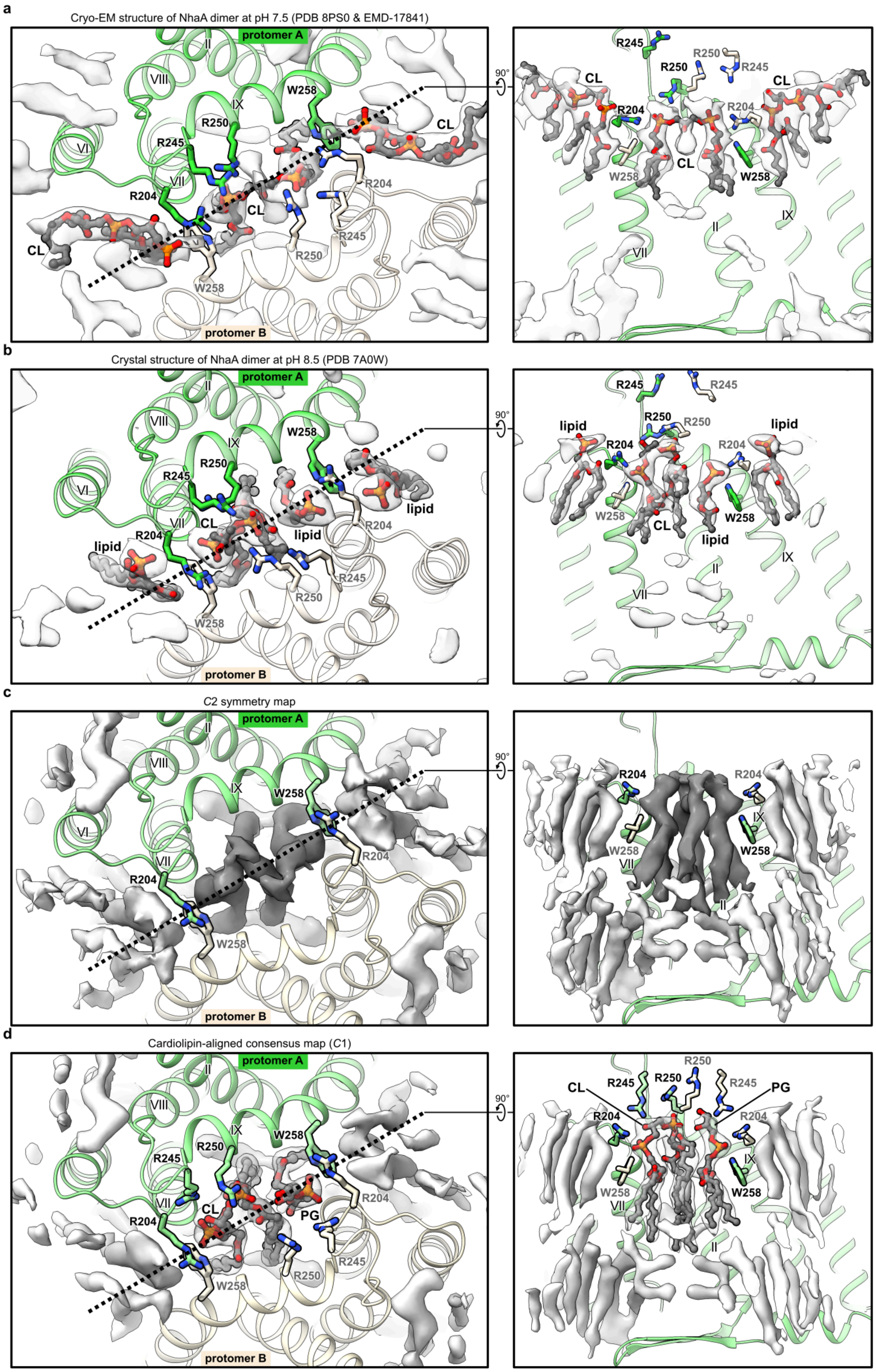
a
Cryo-EM structure of NhaA dimer at pH 7.5 (PDB 8PS0 & EMD-17841)
protomer A
protomer B
II
VIII
VI
VII
IX
W258
R250
R245
R204
CL
90°
b
Crystal structure of NhaA dimer at pH 8.5 (PDB 7A0W)
lipid
c
C2 symmetry map
d
Cardiolipin-aligned consensus map (C1)
PG

Supplementary Fig. 4: Analysis of non-protein density at the dimer interface of NhaA. **a**, Cryo-EM structure of *E. coli* NhaA dimer at pH 7.5 (PDB 8PS0; EMD-17841). **b**, Crystal structure of *S. enterica* NhaA dimer at pH 8.5 (PDB 7A0W). **c**, *C*2 symmetric cryo-EM map of the dimer interface at pH 7.5 in this study. **d**, Cryo-EM structure of NhaA dimer derived from the cardiolipin-aligned consensus map (*C*1) at pH 7.5. Left panels show the dimerization interface viewed from the cytoplasmic side. Right panels show the cross-section of the dimer interface. Lipid molecules are shown in ball-and-stick representation. Interface residues interacting with the phospholipids are shown as sticks. CL, cardiolipin; PG, phosphatidylglycerol.

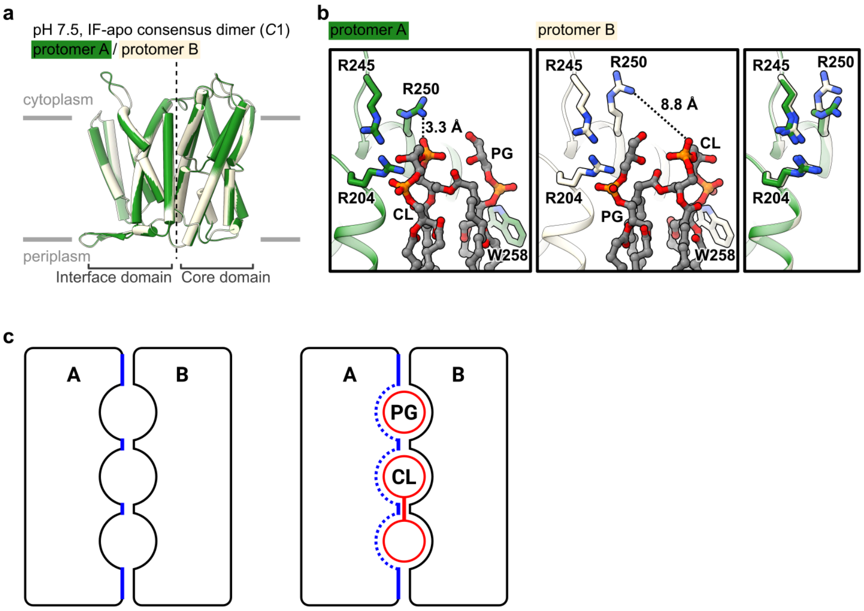

Supplementary Fig. 5: Structural comparison of the two NhaA protomers and the dimer interface. **a**, Superimposition of both protomers in the IF-apo NhaA consensus dimer structure at pH 7.5. **b**, Interactions between the dimer-interface phospholipids and protomer A (left) or protomer B (middle) in the consensus dimer structure. The right panel shows the structural comparison of the dimer interfaces of both protomers. The phospholipids are in ball-and-stick representation. The residues in close contact with the phospholipids at the dimer interface are shown as sticks. **c**, Schematic representation of NhaA dimer without phospholipids (left) and with bound PG and CL (right) at the dimer interface. The contact area at the dimer interface contributed by protomer A is highlighted by blue solid line (direct protein-protein contact) and blue dashed line (contact through phospholipids).

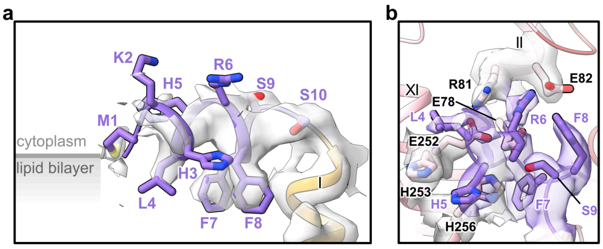

Supplementary Fig. 6: Cryo-EM maps and models of NhaA NT in the IF-apo^{unplugged} (**a**) and IF-apo^{plugged} (**b**) states at pH 7.5 in the presence of K^+.

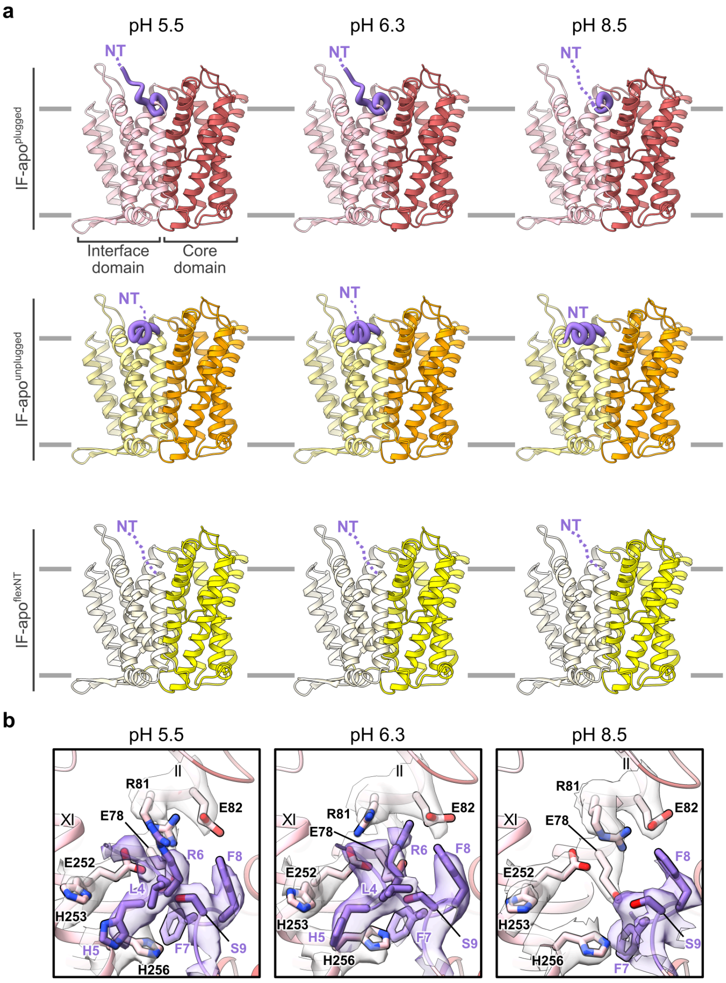

Supplementary Fig. 7: Cryo-EM structures of NhaA protomer determined in different states across a wide pH range. **a**, Atomic models of NhaA protomer determined in the IF-apo^{plugged}, IF-apo^{unplugged}, and IF-apo^{flexNT} states at pH 5.5, 6.3 and 8.5 in the presence of K^{+}. The core and interface domains are colored in different shades of red, orange, and yellow for the IF-apo^{plugged} and IF-apo^{unplugged}, and IF-apo^{flexNT} states, respectively. The NT is shown in worm representation and colored in purple. **b**, Close-up cytoplasmic views of the cryo-EM maps and models of the NT in IF-apo^{plugged} NhaA shown in **a**. From left to right: pH 5.5, 6.3, and 8.5.

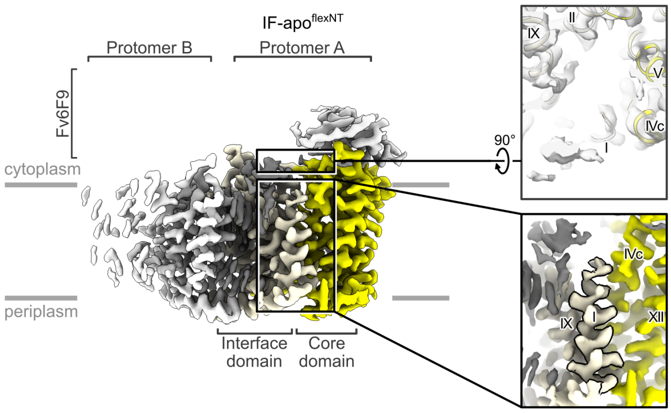

Supplementary Fig. 8: Cryo-EM map of IF-apo^{flexNT} NhaA at pH 7.5 in the presence of K^+. Densities for NhaA are colored in different shades of yellow (protomer A) and white (protomer B), Fv6F9 in light gray, and lipid/non-protein densities in dark gray. The close-up views on the right highlight the cytoplasmic entrance (top) and TM I (bottom).

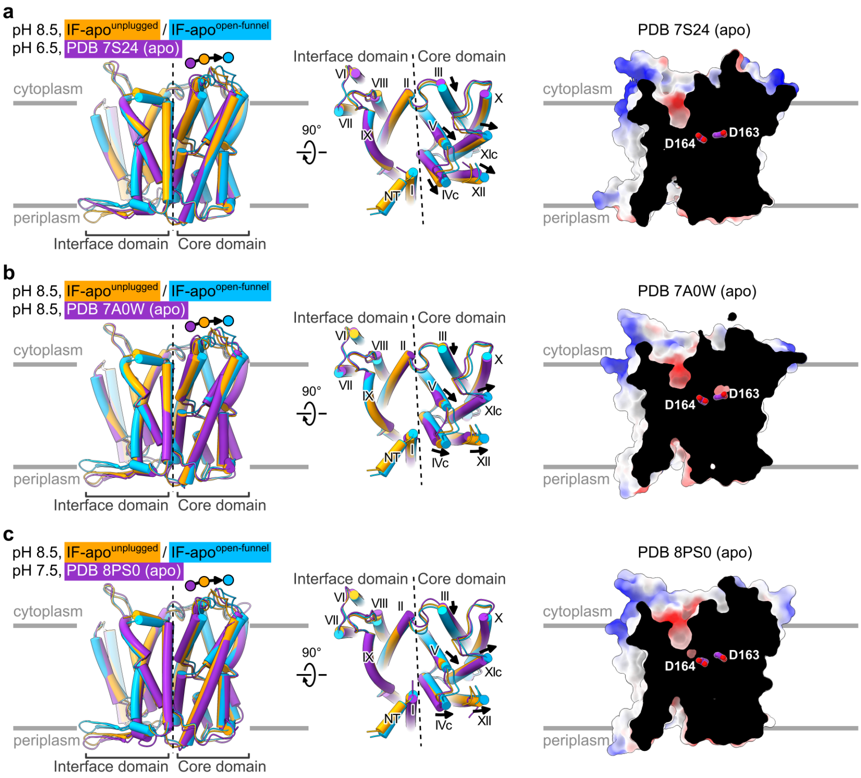

Supplementay Fig. 9: Structural comparisons of the IF-apo^{unplugged} and IF-apo$^{open\text{-}funnel}$ states at pH 8.5 determined in this study to the previously determined crystal structures of *E. coli* NhaA monomer at pH 6.5 (PDB 7S24) (**a**), *S. enterica* NhaA dimer at pH 8.5 (PDB 7A0W) (**b**) and the cryo-EM structure of *E. coli* NhaA dimer at pH 7.5 (PDB 8PS0) (**c**). The right panels are the cut-away views of the respective structures.

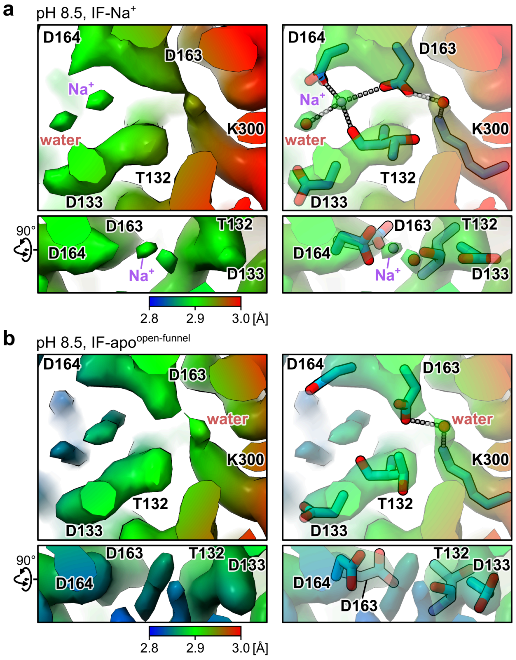

Supplementary Fig. 10: Local-resolution map of the ion-binding site in the IF-Na^+ (**a**) and IF-apo$^{open\text{-}funnel}$ (**b**) states. The cryo-EM maps are filtered and colored by local resolution, and are displayed in the same viewing angle as in Fig. 3e. For clarity, overlays of the ion-binding residues and the transparent cryo-EM maps are shown in the right panels.

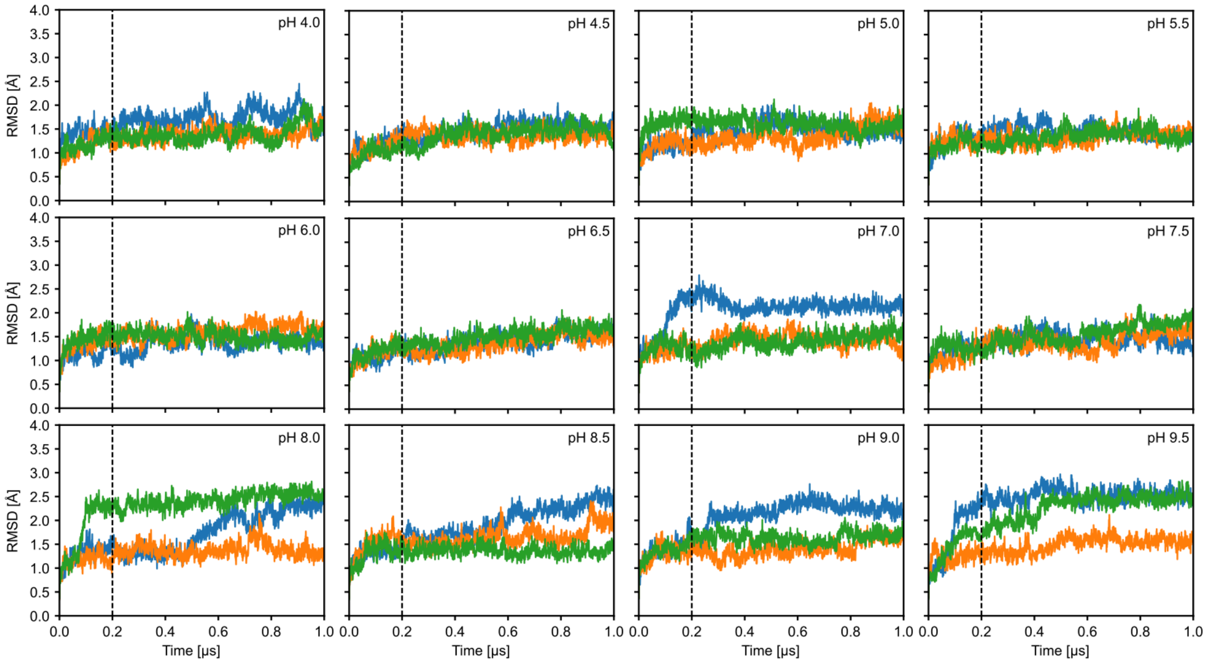

Supplementary Fig. 11: Representative root-mean-square deviation (RMSD) of the C_α atoms in the transmembrane helices of NhaA, as observed in the simulations with Na^+ ion blocked from the ion-binding site. The colors correspond to the three replicas. The first 200 ns of the simulations was discarded for equilibration purposes.

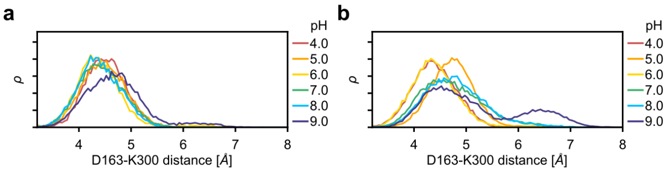

Supplementary Fig. 12: Probability density (*ρ*) of Asp163–Lys300 distance in the constant pH simulations when Na^+ is blocked from entering the cytoplasmic funnel (**a**) or when Na^+ moves freely into the ion-binding site (**b**).

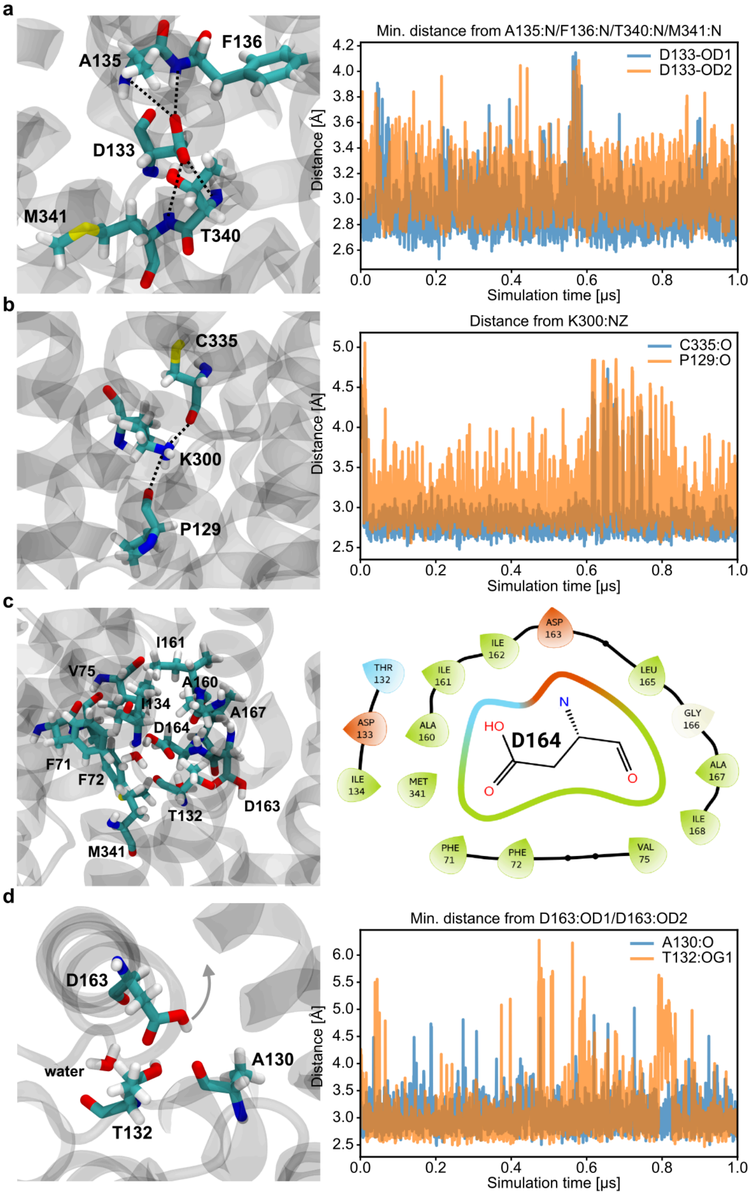

Supplementary Fig. 13: Stabilization of the protonation state of the ion-binding residues in MD simulations. Representative snapshots of the local environment for the ion-binding residues Asp133 (**a**), Lys300 (**b**), Asp164 (**c**) and Asp163 (**d**) are shown. The ion-binding residues and those they interact with or surrounding them are shown as sticks. The right panels show representative MD simulation time series of the ion-binding residues forming electrostatic interactions (Asp133 and Lys300) or hydrogen bonding (Asp163) with nearby residues. For Asp164, a schematic overview of the local hydrophobic environment is shown in the right panel.

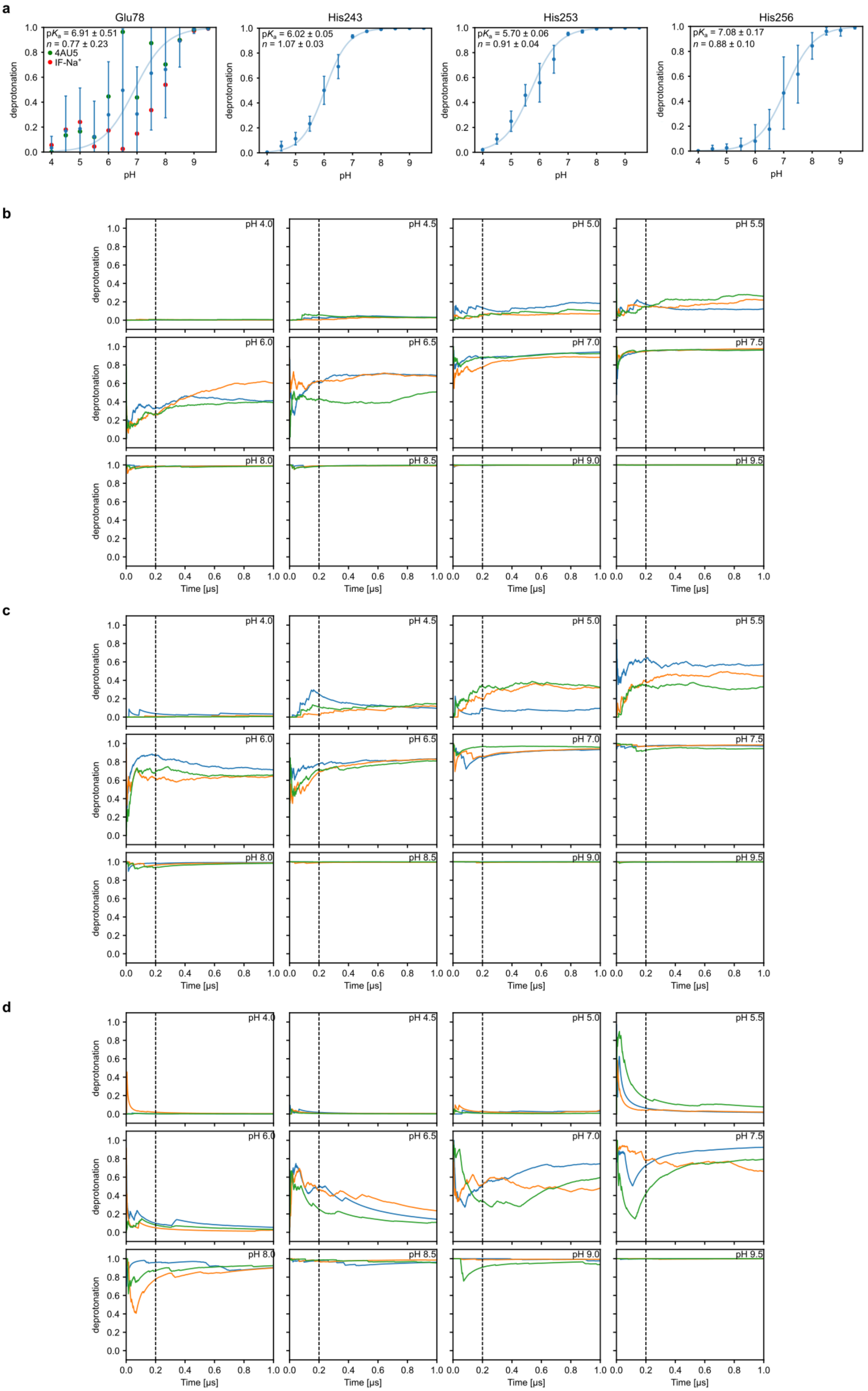
a
Glu78
pKa = 6.91 ± 0.51
n = 0.77 ± 0.23
4AU5
IF-Na+
His243
pKa = 6.02 ± 0.05
n = 1.07 ± 0.03
His253
pKa = 5.70 ± 0.06
n = 0.91 ± 0.04
His256
pKa = 7.08 ± 0.17
n = 0.88 ± 0.10
deprotonation
pH
b
c
d
pH 4.0
pH 4.5
pH 5.0
pH 5.5
pH 6.0
pH 6.5
pH 7.0
pH 7.5
pH 8.0
pH 8.5
pH 9.0
pH 9.5
Time [μs]

Supplementary Fig. 14: Titration curves of the key pH sensor residues. **a**, Titration curves of Glu78, His243, His253 and His256, as jointly observed in the simulations with and without sodium exclusion. For each simulation, the mean deprotonation values (blue circle) were computed after discarding the first 200 ns. The data shown is the average of all the mean values across all the simulations, and the error bar is their standard deviation. For Glu78, the mean values of simulations starting from the low pH crystal structure (PDB 4AU5; green circle) or from the IF-Na^+ structure (red circle) are also presented. Note that discarding the first 500 ns simulations has a negligible effect on the titration curves. The pK_a values were obtained by fitting the data with the Hill equation, with Hill-coefficient *n*[1]. **b**-**d**, Cumulative deprotonation plots of His243 (**b**), His253 (**c**) and His256 (**d**), as observed in the simulations with Na^+ blocked from the ion-binding site. The colors correspond to the three replicas. The first 200 ns of the simulations was discarded for equilibration purposes. The curves at intermediate pH values show increased uncertainty due to the small free energy difference between the protonated and unprotonated forms. Source data for **a** are provided as a Source Data file.

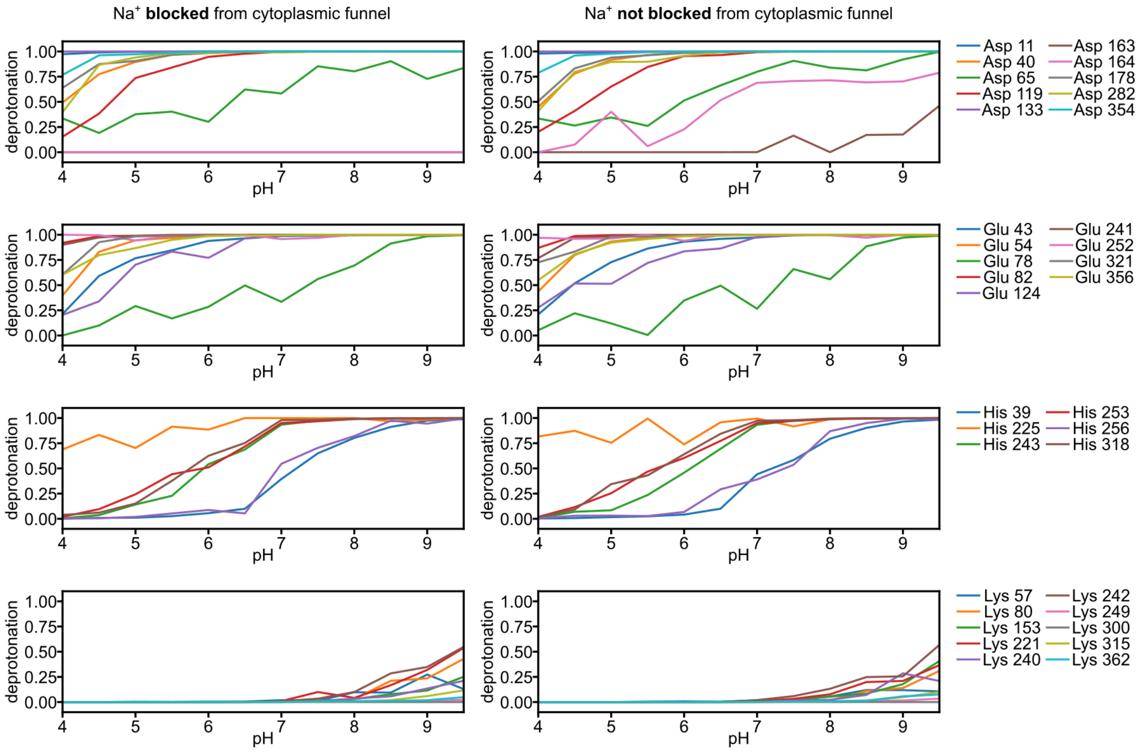

Supplementary Fig. 15: Deprotonation of amino acid residues Asp, Glu, His, and Lys in NhaA in the constant pH simulations, with Na^+ blocked from entering the cytoplasmic funnel (left) or Na^+ moving freely (right). Source data are provided as a Source Data file.

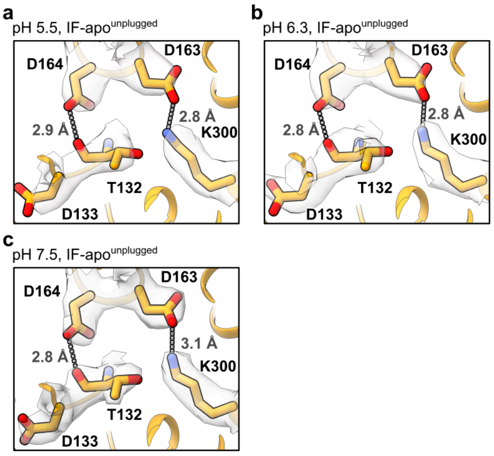

Supplementary Fig. 16: Cryo-EM maps and models of the ion-binding site of IF-apo^{unplugged} NhaA at pH 5.5 (**a**), 6.3 (**b**) and 7.5 (**c**). The ion-binding site residues are shown as sticks.

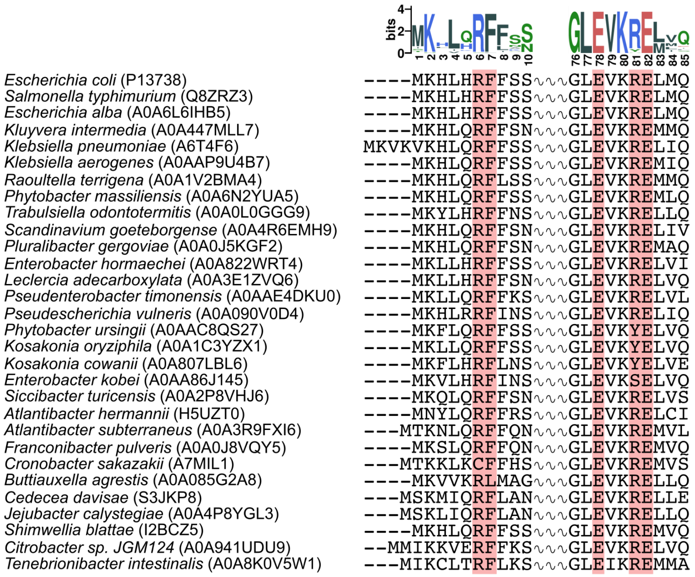

Supplementary Fig. 17: Multiple sequence alignment of NhaA among enterobacteria. The sequences of the NT and part of TM II accommodating Glu78, Arg81 and Glu82 from various enterobacteria and the respective UniProt ID are shown. Residues participating in the NT-TM II interaction are highlighted in red box. Conservation of the sequences are shown as a sequence logo above the alignment, with the residue numbering in *E. coli* NhaA.

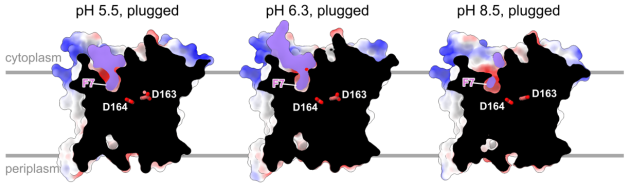

Supplementary Fig. 18: Cut-away views of the IF-apo^{plugged} NhaA at pH 5.5, 6.3 and 8.5 in surface representation showing the cytoplasmic cavity. The NT domain is colored in purple. The ion-binding residues Asp163 and Asp164 are shown as sticks.

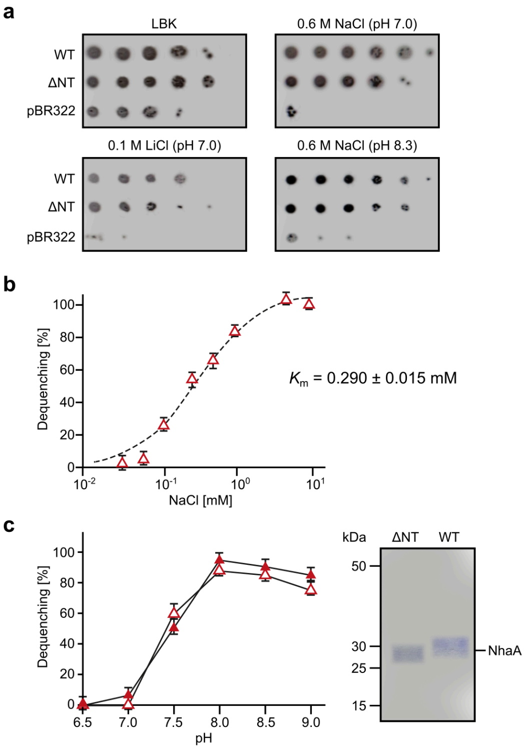

Supplementary Fig. 19: Growth phenotype and transport activity of ΔNT-NhaA. **a**, Growth phenotype of EP432 cells producing WT- or ΔNT-NhaA on LBK or selective media with high salt concentration at different pH values. pBR322 served as a negative control. **b**, Na^+/H^+ antiport activity of ΔNT-NhaA in everted membrane vesicles at pH 8.5, measured as dequenching of acridine orange fluorescence. **c**, Na^+/H^+ antiport activity of WT-NhaA (filled triangle) and ΔNT mutant (open triangle) in everted membrane vesicles at different pH values. The right panel shows the expression level of the indicated proteins by SDS-PAGE stained with Coomassie Blue. n = 3 independent technical replicates. Data shown are mean ± standard deviation (error bars). The uncropped gel is shown in Supplementary Fig. 21.

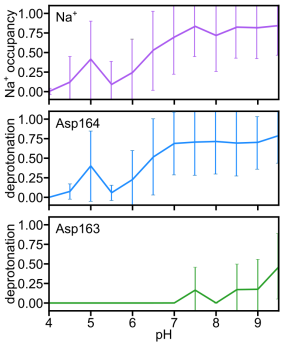

Supplementary Fig. 20: Separate plots displaying the Na^+ occupancy and the deprotonation of Asp164 and Asp163 in cpH-MD simulations with freely moving Na^+ (corresponding to Fig. 3j), with error bars representing one standard deviation. Source data are provided as a Source Data file.

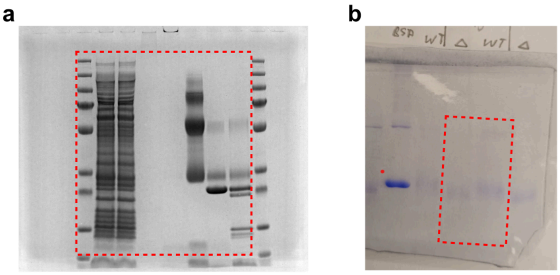

Supplementary Fig. 21: Uncropped scans of gels shown in Supplementary Fig. 1d (**a**) and Supplementary Fig. 19c (**b**). The cropped areas are marked by the red dashed rectangle.

References:

1. Onufriev, A., Case, D. A. & Ullmann, G. M. A Novel View of pH Titration in Biomolecules. *Biochemistry* **40**, 3413–3419 (2001).
