# Supplementary material for: pH-dependent activation of the Na+/H+ antiporter NhaA and conformational dynamics of its N-terminus
Source: Nat Commun. 2026 Jun 12;17:7489. doi: 10.1038/s41467-026-73424-2 (PMC13408996; doi:10.1038/s41467-026-73424-2)
Supplement: Supplementary file 2 — Description of Additional Supplementary Files [file 41467_2026_73424_MOESM2_ESM.pdf]

Description of Additional Supplementary Files

File name: Supplementary Movie 1
Description: Molecular movie of Na^+ binding to the ion-binding site of NhaA in constant pH molecular dynamics simulations. The protein chains are shown in a transparent cartoon representation from the side (left) and from the cytoplasm (right). The titratable residues are shown as sticks, and the Na^+ ion is represented by a yellow van der Waals sphere. The surrounding membrane, waters, and protein hydrogen atoms are omitted for clarity.
