# Supplementary material for: pH-dependent activation of the Na+/H+ antiporter NhaA and conformational dynamics of its N-terminus
Source: Nat Commun. 2026 Jun 12;17:7489. doi: 10.1038/s41467-026-73424-2 (PMC13408996; doi:10.1038/s41467-026-73424-2)
Supplement: Supplementary file 4 — Reporting Summary [file 41467_2026_73424_MOESM4_ESM.pdf]

nature portfolio

Corresponding author(s): Dr. Etana Padan, Dr. Hartmut Michel

Last updated by author(s): Apr 2, 2026

Reporting Summary

Nature Portfolio wishes to improve the reproducibility of the work that we publish. This form provides structure for consistency and transparency in reporting. For further information on Nature Portfolio policies, see our Editorial Policies and the Editorial Policy Checklist.

Statistics

For all statistical analyses, confirm that the following items are present in the figure legend, table legend, main text, or Methods section.

n/a	Confirmed	
☐	☒	The exact sample size (*n*) for each experimental group/condition, given as a discrete number and unit of measurement
☐	☒	A statement on whether measurements were taken from distinct samples or whether the same sample was measured repeatedly
☒	☐	The statistical test(s) used AND whether they are one- or two-sided<br>*Only common tests should be described solely by name; describe more complex techniques in the Methods section.*
☒	☐	A description of all covariates tested
☒	☐	A description of any assumptions or corrections, such as tests of normality and adjustment for multiple comparisons
☐	☒	A full description of the statistical parameters including central tendency (e.g. means) or other basic estimates (e.g. regression coefficient) AND variation (e.g. standard deviation) or associated estimates of uncertainty (e.g. confidence intervals)
☒	☐	For null hypothesis testing, the test statistic (e.g. *F*, *t*, *r*) with confidence intervals, effect sizes, degrees of freedom and *P* value noted<br>*Give P values as exact values whenever suitable.*
☒	☐	For Bayesian analysis, information on the choice of priors and Markov chain Monte Carlo settings
☒	☐	For hierarchical and complex designs, identification of the appropriate level for tests and full reporting of outcomes
☒	☐	Estimates of effect sizes (e.g. Cohen's *d*, Pearson's *r*), indicating how they were calculated

Our web collection on statistics for biologists contains articles on many of the points above.

Software and code

Policy information about availability of computer code

Data collection	Titan Krios microscopes operated at 300 kV and equipped with a BioQuantum energy filter and a K3 camera (Gatan); Data collection quality was monitored through EPU 3.7-3.9.
Data analysis	For cryo-EM data analysis:<br>ChimeraX 1.6 and 1.9; CTFFind 4.1.5; RELION 4.0; COOT 0.9.8; Phenix 1.20.1; MolProbity 4.5; ISOLDE 1.6 and 1.9; GraphPad Prism 10.5.0; Microsoft Excel 16;<br>For molecular dynamics simulation analysis:<br>A constant-pH variant of the CHARMM36m force field was used for protein, lipids, and ions, together with TIP3P water; LINCS algorithm was used to constrain the bonds involving hydrogen atoms; modified GROMACS 2021 (https://gitlab.com/gromacs-constantph/constantph);<br>Simulations were analysed using Python libraries numpy 1.25.2, matplotlib 3.10.5, pandas 2.3.1 and mdanalysis 2.9.0 in jupyter-lab 4.4.6.

For manuscripts utilizing custom algorithms or software that are central to the research but not yet described in published literature, software must be made available to editors and reviewers. We strongly encourage code deposition in a community repository (e.g. GitHub). See the Nature Portfolio guidelines for submitting code & software for further information.

Data

Policy information about availability of data

All manuscripts must include a data availability statement. This statement should provide the following information, where applicable:

- Accession codes, unique identifiers, or web links for publicly available datasets
- A description of any restrictions on data availability
- For clinical datasets or third party data, please ensure that the statement adheres to our policy

Cryo-EM maps and atomic models of E. coli NhaA have been deposited in the Electron Microscopy Data Bank and Protein Data Bank under accession numbers: EMD-53954, PDB 9RH1; EMD-53955, PDB 9RH2; EMD-53956, PDB 9RH3; EMD-53957, PDB 9RH4; EMD-53958, PDB 9RH5; EMD-53959, PDB 9RH6; EMD-53960, PDB 9RH7; EMD-53961, PDB 9RH8; EMD-53962, PDB 9RH9; EMD-53963, PDB 9RHA; EMD-53964, PDB 9RHB; EMD-53965, PDB 9RHC; EMD-53966, PDB 9RHD; EMD-53967, PDB 9RHE; EMD-53968, PDB 9RHF.
All molecular dynamics simulation input files for this study are deposited in a Zenodo repository and freely available via the following DOI: 10.5281/zenodo.19354200.

Research involving human participants, their data, or biological material

Policy information about studies with human participants or human data. See also policy information about sex, gender (identity/presentation), and sexual orientation and race, ethnicity and racism.

Reporting on sex and gender	N/A
Reporting on race, ethnicity, or other socially relevant groupings	N/A
Population characteristics	N/A
Recruitment	N/A
Ethics oversight	N/A

Note that full information on the approval of the study protocol must also be provided in the manuscript.

Field-specific reporting

Please select the one below that is the best fit for your research. If you are not sure, read the appropriate sections before making your selection.

☒ Life sciences ☐ Behavioural & social sciences ☐ Ecological, evolutionary & environmental sciences

For a reference copy of the document with all sections, see nature.com/documents/nr-reporting-summary-flat.pdf

Life sciences study design

All studies must disclose on these points even when the disclosure is negative.

Sample size	Sample sizes (number of collected micrographs) of respective cryo-EM datasets were chosen based on instrument availability and experimental design. Datasets of > 8000 micrographs ensured a sufficient number of particles to achieve resolutions < 3.5 Å. The smallest collected dataset contained 9756 micrographs (NhaA pH 5.5-K+ dataset) while the largest dataset contained 19873 micrographs (NhaA pH 8.5-K+ dataset).
Data exclusions	No data were excluded.
Replication	Single particle cryo-EM is based on averaging protein particles of nearly identical orientation within a vitreous layer of ice. Therefore, replication is not per se required to ensure statistical robustness of structural data. In case of this work, we have determined 15 individual structures of NhaA under different sample conditions. Hence these data can be considered as biological replicates of the presented structural data.<br>For constant pH MD simulations, all conditions were performed with three replicates.<br>For cell growth phenotype assay and antiport activity assay, n = 3 technical replicates.<br>All reported findings were reproducible, and no inconsistencies were found.
Randomization	Generally, no randomization was required for the experimental design of this study. However, it is to note that particles were randomized during data processing steps in Relion (randomization during 2D classification; randomized half sets during 3D refinement). Randomized half sets of particles were used in final reconstruction steps in order to determine Fourier shell correlations based on the gold-standard 0.143 level.
Blinding	For all studies in this manuscript, such as 3D reconstruction, there was no awareness of group assignment that caused biased results, so blinding was not relevant for data reliability.

Reporting for specific materials, systems and methods

We require information from authors about some types of materials, experimental systems and methods used in many studies. Here, indicate whether each material, system or method listed is relevant to your study. If you are not sure if a list item applies to your research, read the appropriate section before selecting a response.

Materials & experimental systems

n/a	Involved in the study
☐	☒ Antibodies
☒	☐ Eukaryotic cell lines
☒	☐ Palaeontology and archaeology
☒	☐ Animals and other organisms
☒	☐ Clinical data
☒	☐ Dual use research of concern
☒	☐ Plants

Methods

n/a	Involved in the study
☒	☐ ChIP-seq
☒	☐ Flow cytometry
☒	☐ MRI-based neuroimaging

Antibodies

Antibodies used

Variable fragment of monoclonal antibody 6F9, derived from mouse IgG, produced in E. coli. This was only used as a fiducial marker for cryo-EM analysis, and the amount used was described in the Method section.

Validation

Padan, E., Venturi, M., Michel, H. & Hunte, C. Production and characterization of monoclonal antibodies directed against native epitopes of NhaA, the Na+/H+ antiporter of Escherichia coli. FEBS Lett 441, 53–58 (1998).

Plants

Seed stocks

Report on the source of all seed stocks or other plant material used. If applicable, state the seed stock centre and catalogue number. If plant specimens were collected from the field, describe the collection location, date and sampling procedures.

Novel plant genotypes

Describe the methods by which all novel plant genotypes were produced. This includes those generated by transgenic approaches, gene editing, chemical/radiation-based mutagenesis and hybridization. For transgenic lines, describe the transformation method, the number of independent lines analyzed and the generation upon which experiments were performed. For gene-edited lines, describe the editor used, the endogenous sequence targeted for editing, the targeting guide RNA sequence (if applicable) and how the editor was applied.

Authentication

Describe any authentication procedures for each seed stock used or novel genotype generated. Describe any experiments used to assess the effect of a mutation and, where applicable, how potential secondary effects (e.g. second site T-DNA insertions, mosiacism, off-target gene editing) were examined.
