# Supplementary material for: pH-dependent activation of the Na+/H+ antiporter NhaA and conformational dynamics of its N-terminus
Source: Nat Commun. 2026 Jun 12;17:7489. doi: 10.1038/s41467-026-73424-2 (PMC13408996; doi:10.1038/s41467-026-73424-2)
Supplement: Supplementary file 5 — Transparent Peer Review file [file 41467_2026_73424_MOESM5_ESM.pdf]

nature portfolio

Peer Review File

pH-dependent activation and the newly revealed N-terminus of the Na^+/H^+ antiporter NhaA

Corresponding Author: Professor Hartmut Michel

This file contains all reviewer reports in order by version, followed by all author rebuttals in order by version.

Version 0:

Reviewer comments:

Reviewer #1

(Remarks to the Author)
This manuscript presents a combined cryo-EM and molecular dynamics investigation of NhaA, a prototypical sodium-proton antiporter. I find it exciting that single-particle cryo-EM can now capture conformational changes across multiple pH conditions, providing valuable structural insights into the pH-dependent mechanism of this important transporter. While I cannot comment extensively on the cryo-EM data quality due to limited expertise in this area, the findings appear significant. I recommend publication following major revision to improve the computational work which in the current form is low quality and lacks rigor; I am not sure if it is correct. Specific concerns are given below.
1. Details of the constant pH MD simulations need to be given, e.g., what residues are allowed to titrate.
2. Convergence needs to be established. Root-mean-square deviation of Ca atoms of the protein vs simulation times; cumulative protonation fraction of key titratable residues (e.g., two aspartates, lysine and histidines that are discussed in the paper) vs time (example see Henderson et al, https://livecomsjournal.org/index.php/livecoms/article/view/v4i1e1563).
3. Simply discarding 200 ns and using the rest for analysis is inappropriate. The data before plateauing of key quantities should be discarded.
4. It is very strange that both Asp163 are Lys300 are always protonated in the entire pH range. I suspect this is due to the deficiency of the constant pH MD implementation in GROMACS which to my knowledge has not been validated for Asp/Glu/Lys with large pKa shifts (particularly for lysine). I can be proven wrong, if the author cites a publication. At least, the authors need to acknowledge that the protonation states may not be accurately determined. I suggest the author compare the CpHMD method with that of Harris et al, J Chem Theory Comput 2022 (https://pubs.acs.org/doi/10.1021/acs.jctc.2c00586).
5. Histidines at the entrance are interesting. I suggest the authors compare their titration behavior with that discussed in Huang et al, Nat Commun 2016.

Reviewer #2

(Remarks to the Author)
The manuscript by Weng, Fabian and colleagues present the first sodium-proton antiporter (CPA) structure with the substrate Na+ ion experimentally resolved, namely the prokaryotic EcNhaA (probably the most extensively studied CPA family member). The authors generated a large data set of atomic resolution structure over the functionally relevant pH range 4 - 9; NhaA is active above pH 6.5 and so the structure provide direct insight into the effect of pH on activity. Although all structures are in the inward facing (IF) conformation (the outward facing structure of NhaA remains elusive), there are a number of novel aspects of the work. In particular, for the first time the N-terminal tail (NT) of NhaA is resolved and shown to be able to plug the access funnel to the binding site.

The major results of the paper are:

1. The NT may restrict access to the intracellular binding site in a pH-dependent manner whereby only at high alkaline pH (~8.5) the NT rarely plugs the access funnel. Although a mutant lacking the NT is also functional and comparable to WT, this work makes a good case for a role of the NT in pH regulation of NhaA activity.

2. The beautiful structural analysis (in combination with the MD) directly confirms the role of the "pH sensor residues" in

widening of the access funnel with increasing pH. Earlier work (which is fully discussed by the authors) had come to the same conclusion but did not present as conclusive evidence over the full relevant pH range as done in this work. Even at pH 8.5 without Na+ (apo) there appears to exist an additional small conformational transition between a "narrower" conformation (IF-apo[unplugged]) of the funnel and an "open funnel" conformation (IF-apo[open-funnel]) that provides full solvent access to D163/K300.

3. The high resolution structure of NhaA at fully active pH 8.5 in the presence of 300 mM NaCl directly shows the binding site of Na+ near the conserved residues D164, D163, and T132; these residues have been previously implicated in binding based on functional studies and simulations. The apo[open funnel] and Na+-bound structures at pH 8.5 Na+ are structurally very similar [which may indicate an example of "conformational shift" where the apo state contains fluctuations that resemble the holo state]. A distinct difference between these two states and the IF-apo[unplugged] conformation is that the latter contains a saltbridge between D163 and K300 whereas this interaction appears weakened or broken in the former.

Overall, the authors contribute interesting new facets to the mechanisms of pH dependence and Na+ transport in NhaA (and similar transporters). I appreciated that in cases where they do not come to the same conclusions as other studies (e.g., specific roles of D163, D164, and K300) they present their work scholarly and discuss it in the context of the existing literature.

I only have a number of minor comments:

1) Fig 2:

This is a large number of structures at different pH (5.5 - 8.5) with careful analysis of the heterogenous populations and Fig 2f & g are a great example of visual design!

* In caption of Fig 2 "theoretical ratios based on *d*" --- should this be *f*?

* Please explain (e.g., in Methods) how the theoretical ratios are computed.

2) The proposed regulation through the N-terminal tail was observed in structures in K+ and absence of Na+. Does Na+ affect the NT and prevent the plugged state?

3) cpH-MD calculations:

* What are the estimated pKa's for all residues listed in Suppl Fig 4?

* Are these calculations converged? For instance, calculate pKa's as a function of cumulative simulated time.

4) Fig 3e: What is the evidence that the "elongated density blob" between D164 and T132 in the IF-apo[open funnel] (middle) structure is not a K+ ion whereas the "spherical density" in the IF-Na+ structure is indeed a Na+ ion?

Admittedly, the structural data and the MD at pH 8.5 seem to be agreeing pretty well. Is there additional evidence from the MD simulations, e.g. do Na+ spontaneously bind to IF-apo[open funnel] and generate the IF-Na+ binding mode?

5) Fig 3g: Add protons for the most likely protonation states of D164, D163, D133, K300. (Otherwise it looks as if they are all deprotonated which does not seem to reflect the data in Suppl Fig 4.)

6) Suppl Fig 4: cpH-MD:

* Asp164 is protonated even above pH 9 in the absence of Na+ near the binding site, even though the residue is water accessible. Given that the pKa of Asp in water is ~4, retaining a proton up to pH 9.5 is a bit surprising. Which interactions stabilize the protonated Asp? Similarly, which interactions maintain the protonation of Asp163 over the whole pH range (without Na+)?

* It looks as if K300 is always protonated. Which interactions stabilize K300 in its charged state, especially given that D163 is mostly protonated and thus neutral and can only form a hydrogen bond? It appears physically inconsistent that a negative charge on D163 is unfavorable while the positive charge on K300 (even deeper inside the protein) is favored in the cpH-MD simulation.

7) Please clarify what "Na+ not blocked from cytoplasmic funnel" means for the presence of Na+ near the binding site. What fraction of the simulation time is Na+ bound to D164 and/or D163?

Reviewer #3

(Remarks to the Author)
This comprehensive study by Weng, Fabian and colleagues reports the pH dependent structural rearrangements of Escherichia coli NhaA, a prototypical sodium-proton exchanger. Using single particle cryo-EM of NhaA dimers in complex with a conformation-specific monoclonal antibody fragment, the authors identified several previously unrevealed structural features such as the asymmetric binding of two different lipids at the symmetric dimer interface and the N-terminal tail, which can act as a plug to seal the transport pathway. Population analysis of particles imaged at pH conditions ranging from 5.5 to 8.5 reveal that the occupancy of the N-terminal plug is pH-dependent, with open state being dominant at pH 8.5. Consistent with the cryo-EM analysis, the N-terminal tail of NhaA can stably occupy the pathway in molecular dynamics simulations. The structures and the molecular dynamics simulations show that the funnel of NhaA gradually opens by breaking a bond between E78 and H256 as the pH increases, revealing how pH regulates transports activity. This paper is well-written and represents a significant advance in the understanding of the activation of NhaA. I have several comments listed below that should be addressed prior to publication that would strengthen the report.

Comments
Major points
1. One of the major strengths of this report is the diversity of functional states presented. However, on line 25 the authors note that the structures were determined in complex with a conformation-specific antibody fragment. What are the functional effects of the Fab? If the Fab is indeed conformation-specific, how was the ensemble of states presented here captured? The authors should evaluate the functional role of the Fab and clarify its effects on transport to ensure that the ensemble of states presented here correspond to physiologically relevant states.
2. The use of symmetry expansion to resolve an asymmetric arrangement of the lipids at the dimer interface is remarkable. Were there any alternative populations present among the particles or did every particle have the same combination of lipids at the interface? Additionally, were there any changes in the conformation of the protein in response to the asymmetric positioning of the lipids?
3. How were densities assigned as ions or water molecules? At the reported overall resolutions of 2.7 to 3.7 Å, it can be different to unambiguously assign non-protein densities. For example, there are densities in middle panel of Fig 3e that are very similar to those present in the right panel, but a Na+ and a water are assigned in only the right panel. It would be helpful to clarify these details and perhaps include local resolution maps to demonstrate that the features are sufficiently well resolved for modelling.

Minor points
1. Line 172. Is Fig 2d correct? Not Fig 2 b,c?
2. Line 174. Fig 2d need to be here?
3. It would be helpful to readers to include an overall description of the important features of the structure when you describe overall structure. For example, the “ -hairpin” is first presented on line 199 when it is being evaluated in detail.

Reviewer #4

(Remarks to the Author)
The manuscript by Weng, Michel and colleagues reports the cryo-EM structures of the Escherichia coli Na^+/H^+ antiporter NhaA, capturing its conformational ensemble across a physiological pH range. The structural determination is executed with high quality, and the authors delineate distinct conformational states corresponding to different pH conditions, thereby revealing the molecular transitions underlying pH-dependent activation. Notably, the visualization of the previously unresolved N-terminal tail represents a significant advancement, providing new insight into the gating and activation process of NhaA.
Major:
1. It is proposed that the N-terminal tail (NT) dissociates as pH increases, a notion further supported by molecular dynamics simulations. However, functional assays indicate that the NT mutant exhibits activity comparable to the wild type. This apparent discrepancy warrants further investigation.
2. Molecular dynamics simulations were employed to identify the sodium-binding site. To definitively confirm its functional role, it would be compelling to complement these computational findings with experimental mutagenesis of the key residues.
3. The author mentioned that obtaining the structure of IF-apo open-funnel state may be the optimization of purification conditions. Given that all resolved structures correspond to inward-facing conformations, exploring alternative approaches to stabilize or visualize the outward-facing state would further enhance the understanding of the complete transport cycle.
4. The authors note that the interfacial lipids between dimers could be assigned to different classes depending on the classification scheme and symmetry applied. However, the basis for these assignments requires further clarification: The reasoning behind the suggestion of dimerization interface asymmetry, based solely on "neighboring densities resembling conventional diacyl phospholipids," is unclear. A more detailed explanation is needed. Please clarify the specific criteria used to distinguish cardiolipin (CL) density from that of diacyl phospholipids.
Minor:
1. The middle panel of Figure e in Fig3 should be the structure of IF-apo-funnel. There are also two blocks between D164 and T132 whose density is similar to the density of the sodium ion binding position in the right figure. How the position of the

Na+ binding sodium ion is determined ?
2. In the supplementary tables, the listed “Map sharpening B factor” is the Gaussian temperature factor applied to the final reconstruction to restore high-resolution contrast. By convention it is reported as a positive number (units $Å^2$); a negative sign simply means the map was over-sharpened (i.e. an artificial negative B was added). Please double-check the actual B-factor values and report them accurately.
3. In addition, the authors imposed C2 symmetry at an early stage of the processing workflow. For the pH 5.5–K+ dataset, Fv6F9 density is absent from one protomer before C2 symmetry is applied, suggesting that the two NhaA monomers may adopt distinct conformations. Imposing C2 symmetry at the outset might force these different states into a single, artefactual average. It is recommended re-processing this dataset in C1, examining class distributions carefully, and providing a clear explanation for why Fv6F9 is missing on one side before any symmetry is enforced.
4. What are the other bands corresponding to Peak A, Peak B, and Peak C on the SDS-PAGE gel in Extended Data Fig. 1?
5. Conventionally, the periplasm is oriented upward and the cytoplasm downward in structural figures, as this aligns with standard visual conventions in membrane biology. If the authors opt not to adjust the orientation, they should provide comparative example figures to explicitly justify their chosen layout (e.g., to better illustrate specific structural details or functional processes).
6. The scale bar for each cryo-EM micrograph in the processing workflow (Supplementary Fig. 1 a-e) should be added.
7. The "Supplementary Fig. 14" mentioned on lines 478 and 493 may not be included in the available materials; please provide the correct citation.
8. CαBLAM number is missing from the Cryo-EM table.
9. The angular distribution of particles used in the final 3D reconstruction should be shown.

Reviewer #5

(Remarks to the Author)
I co-reviewed this manuscript with one of the reviewers who provided the listed reports. This is part of the Nature Communications initiative to facilitate training in peer review and to provide appropriate recognition for Early Career Researchers who co-review manuscripts.

Version 1:

Reviewer comments:

Reviewer #1

(Remarks to the Author)
The authors have adequately addressed my comments.

Reviewer #2

(Remarks to the Author)
The authors addressed my previous comments. Thank you.

Minor correction:

Suppl Fig 10: Caption says "displaying standard deviations of Na+ occupancy and deportonation". This seems ambiguous: It looks to me as if the occupancy and deprotonation is shown and error bars correspond to one SD.

Comments:

Glu78: Winkelmann et al, Nature Comm 2022 include it in the sensor residues and show that the E78A mutant has a different pH activation profile WT, supporting the authors interpretation of Glu78 as a pH sensor residue.

Reviewer #3

(Remarks to the Author)
The authors have addressed all concerns and the manuscript is suitable for publication.

Reviewer #4

(Remarks to the Author)

The authors have addressed the previous comments by providing additional clarifications and supporting references. These revisions improve the clarity and presentation of the manuscript.
minor:
1.The authors stated that scale bars have been added to Supplementary Figure 1; however, these are still absent from the revised figure.
2. For clarity and reproducibility, please confirm that the exact map sharpening B-factor values applied to each reconstruction are explicitly reported in the Methods section.

Reviewer #5

(Remarks to the Author)
I co-reviewed this manuscript with one of the reviewers who provided the listed reports. This is part of the Nature Communications initiative to facilitate training in peer review and to provide appropriate recognition for Early Career Researchers who co-review manuscripts.

Open Access This Peer Review File is licensed under a Creative Commons Attribution 4.0 International License, which permits use, sharing, adaptation, distribution and reproduction in any medium or format, as long as you give appropriate credit to the original author(s) and the source, provide a link to the Creative Commons license, and indicate if changes were made.
In cases where reviewers are anonymous, credit should be given to 'Anonymous Referee' and the source.
The images or other third party material in this Peer Review File are included in the article’s Creative Commons license, unless indicated otherwise in a credit line to the material. If material is not included in the article’s Creative Commons license and your intended use is not permitted by statutory regulation or exceeds the permitted use, you will need to obtain permission directly from the copyright holder.
To view a copy of this license, visit https://creativecommons.org/licenses/by/4.0/

REVIEWER COMMENTS

Reviewer #1 (Remarks to the Author):

This manuscript presents a combined cryo-EM and molecular dynamics investigation of NhaA, a prototypical sodium-proton antiporter. I find it exciting that single-particle cryo-EM can now capture conformational changes across multiple pH conditions, providing valuable structural insights into the pH-dependent mechanism of this important transporter. While I cannot comment extensively on the cryo-EM data quality due to limited expertise in this area, the findings appear significant. I recommend publication following major revision to improve the computational work which in the current form is low quality and lacks rigor; I am not sure if it is correct. Specific concerns are given below.

1. Details of the constant pH MD simulations need to be given, e.g., what residues are allowed to titrate.

- We thank the reviewer for this question. Indeed, all Glu, Asp, His, Lys and Arg residues were allowed to titrate during the simulations. This is now explicitly stated in the methods section:

  "During the simulations, all Asp, Glu, His, Lys, and Arg residues were allowed to titrate. All cpH-MD-related simulation parameters were adopted from Aho et al.[39]. In particular, the mass of the λ-particle was 5 a.u. and the coupling time was set to 2 ps."

2. Convergence needs to be established. Root-mean-square deviation of Ca atoms of the protein vs simulation times; cumulative protonation fraction of key titratable residues (e.g., two aspartates, lysine and histidines that are discussed in the paper) vs time (example see Henderson et al, https://livecomsjournal.org/index.php/livecoms/article/view/v4i1e1563).

- Following the reviewer's recommendation, we now show the C_α RSMD curves and cumulative protonation fraction of the histidine residues from our cpH-MD simulations in Supplementary Figs. 5 and 7, respectively. If Na^+ is excluded from the ion binding site, Asp163 and Asp164 do not titrate over the studied pH range, and always stay protonated (Fig. 3j). When Na^+ is allowed to enter the binding site, its presence strongly correlates with the deprotonation of Asp163 and Asp164 (Fig. 3j). Therefore, Asp163 and Asp164 do not exhibit regular titration behavior, but are simply drawn towards the lambda values of 0 (protonated) or 1 (deprotonated) depending on Na^+ proximity. In our simulations, Lys300 remains protonated across the entire pH range, with or without Na+ in the binding site (Supplementary Fig. 8). Now the methods section reads:

  "Convergence was assessed based on the RMSD of the C_α atoms involved in the TM helices and on the protonation state of the titratable residues, shown in Supplementary Figs. 5 and 7."

  The standard deviation of Na^+ occupancy, and the titration profiles for Asp163 and Asp164 across the N=6 replicas is now shown in Supplementary Fig. 10.

3. Simply discarding 200 ns and using the rest for analysis is inappropriate. The data before plateauing of key quantities should be discarded.

- We agree with the overall assessment of the reviewer. While long by simulations standards, the 200 ns cutoff is somewhat arbitrary. To justify this cutoff, we repeated the analysis of pK_a values by discarding the first 500 ns of the simulations. The longer cutoff had no appreciable effect on the pK_a values. However, at the highest pH values, the C_α RSMD curves (Supplementary Fig. 5) showed conformational changes also beyond 200 ns. These changes and the need for longer simulations is now highlighted in the methods section as:

  "Discarding the first 500 ns instead of 200 ns did not affect the extracted pK_a values, which are collected in Supplementary Table 3. While the behavior of titratable residues appears to be converged, the C_α RSMD show conformational dynamics at the highest pH beyond 200 ns. Although clarifying these motions would be highly desirable, we were limited by the current performance of our cpH-MD implementation."

4. It is very strange that both Asp163 are Lys300 are always protonated in the entire pH range. I suspect this is due to the deficiency of the constant pH MD implementation in GROMACS which to my knowledge has not been validated for Asp/Glu/Lys with large pKa shifts (particularly for lysine). I can be proven wrong, if the author cites a publication. At least, the authors need to acknowledge that the protonation states may not be accurately determined. I suggest the author compare the CpHMD method with that of Harris et al, J Chem Theory Comput 2022 (https://pubs.acs.org/doi/10.1021/acs.jctc.2c00586).

- As the reviewer correctly points out, capturing the coupling between conformation (hydration state, strong coulombic interactions, ion access) is a challenging problem, even more so in the case of large pK_a shifts. While our implementation indeed has not been validated for residues subject to large pK_a shifts, it is highly similar to the one developed by Harris et al 2022. Both of them are explicit, all-atom cpH-MD codes using charge interpolation with PME. Some differences are:
  - The treatment of the net charge of the system: Harris et al.[3] include the corresponding electrostatic contribution (V_{plasma}) while we rely on buffer particles to absorb the excess charge.
  - The inclusion of the Lennard-Jones energy between the various states.

  While arguably beneficial for the entire simulation community, an explicit comparison of simulating the same protein would require a common force field between them, and is out of the scope of this work. This is highlighted in the manuscript as:

  "While our cpH-MD implementation is not formally validated for very large pK_a shifts, the algorithm closely resembles that of Harris et al.[72]: both of them are explicit, all-atom cpH-MD implementations using charge interpolation with Particle-Mesh-Ewald (PME), with the latter being able to capture the large pK_a shift of Asp26 in thioredoxin."

5. Histidines at the entrance are interesting. I suggest the authors compare their titration behavior with that discussed in Huang et al, Nat Commun 2016.

- We thank the reviewer for their recommendation. Indeed, the order of histidine pK_a values (His243: 6.0; His253: 5.7; His256: 7.1) matches that of Huang et al.[1]

(His243: 6.8; His253: 6.3; His256: 6.9), but overall shifted to somewhat lower values. Previously, Huang et al. determined the pK_a of Glu78 to be 3.1, and ruled it out from the list of pH-sensing residues. In our simulations, the pK_a of Glu78 is 6.9 (Supplementary Fig. 7a), indicating that it can contribute to pH sensing. The extracted pK_a values are robust: their change is negligible even when the first 500 ns of the simulations are discarded. These results have been added to the manuscript as:

"The cpH-MD simulations report pK_a values for Glu78 (6.9), His243 (6.0), His253 (5.7), and His256 (7.1) in the physiological range (Supplementary Fig. 6, Supplementary Table 3). While the values for histidines are in good agreement with those obtained by Huang et al. (Glu78: 3.1; His243: 6.8; His253: 6.3; His256: 6.9)[57], our calculations support a more active role for Glu78 in pH sensing. The comparably large scatter in the deprotonation values of Glu78 across the different replicates (Supplementary Fig. 7a) mostly reflects differences in the initial structures, being about 0.5 pK_a units lower when starting from the low pH crystal structure (PDB 4AU5) compared to the new IF-Na^+ structure."

Reviewer #2 (Remarks to the Author):

The manuscript by Weng, Fabian and colleagues present the first sodium-proton antiporter (CPA) structure with the substrate Na+ ion experimentally resolved, namely the prokaryotic EcNhaA (probably the most extensively studied CPA family member). The authors generated a large data set of atomic resolution structure over the functionally relevant pH range 4 - 9; NhaA is active above pH 6.5 and so the structure provide direct insight into the effect of pH on activity. Although all structures are in the inward facing (IF) conformation (the outward facing structure of NhaA remains elusive), there are a number of novel aspects of the work. In particular, for the first time the N-terminal tail (NT) of NhaA is resolved and shown to be able to plug the access funnel to the binding site.

The major results of the paper are:

1. The NT may restrict access to the intracellular binding site in a pH-dependent manner whereby only at high alkaline pH (~8.5) the NT rarely plugs the access funnel. Although a mutant lacking the NT is also functional and comparable to WT, this work makes a good case for a role of the NT in pH regulation of NhaA activity.

2. The beautiful structural analysis (in combination with the MD) directly confirms the role of the "pH sensor residues" in widening of the access funnel with increasing pH. Earlier work (which is fully discussed by the authors) had come to the same conclusion but did not present as conclusive evidence over the full relevant pH range as done in this work. Even at pH 8.5 without Na+ (apo) there appears to exist an additional small conformational transition between a "narrower" conformation (IF-apo[unplugged]) of the funnel and an "open funnel" conformation (IF-apo[open-funnel]) that provides full solvent access to D163/K300.

3. The high resolution structure of NhaA at fully active pH 8.5 in the presence of 300 mM NaCl directly shows the binding site of Na+ near the conserved residues D164, D163, and T132; these residues have been previously implicated in binding based on functional studies and simulations. The apo[open funnel] and Na+-bound structures at pH 8.5 Na+ are structurally very similar [which may indicate an example of

"conformational shift" where the apo state contains fluctuations that resemble the holo state]. A distinct difference between these two states and the IF-apo[unplugged] conformation is that the latter contains a saltbridge between D163 and K300 whereas this interaction appears weakened or broken in the former.

Overall, the authors contribute interesting new facets to the mechanisms of pH dependence and Na+ transport in NhaA (and similar transporters). I appreciated that in cases where they do not come to the same conclusions as other studies (e.g., specific roles of D163, D164, and K300) they present their work scholarly and discuss it in the context of the existing literature.

I only have a number of minor comments:

1) Fig 2:

This is a large number of structures at different pH (5.5 - 8.5) with careful analysis of the heterogenous populations and Fig 2f & g are a great example of visual design!

* In caption of Fig 2 "theoretical ratios based on ***d***" --- should this be ***f***?

- We thank the reviewer for their positive opinion on the figure and pointing out the typo. The caption of Fig. 2g is now corrected.

* Please explain (e.g., in Methods) how the theoretical ratios are computed.

- We have added a paragraph in Methods under *Cryo-EM image processing* section to explain the calculation of the theoretical ratios, as follows:

  "Assuming no allosteric coupling between protomers, the theoretical (non-allosteric) ratios of NhaA dimer states (Fig. 2g) were calculated from the fractional populations of individual protomer states (P_{state}; Fig. 2f) as all pairwise products of the four possible states, corresponding to the expansion of

  $$\left(P_{plugged} + P_{flexNT} + P_{unplugged} + P_{open-funnel}\right)^2$$

  This yields both homodimeric (e.g., ${P_{plugged}}^2$) and heterodimeric (e.g., $2P_{plugged} \times P_{unplugged}$) combinations expected for independent protomers."

2) The proposed regulation through the N-terminal tail was observed in structures in K+ and absence of Na+. Does Na+ affect the NT and prevent the plugged state?

- It is highly likely that Na^+ affects the NT, since in our pH 8.5-Na^+ cryo-EM dataset only the IF-Na^+ state, in which the NT is unplugged, was found, whereas three states (IF-apo^{plugged}, IF-apo^{unplugged} and IF-apo$^{open\text{-}funnel}$) were found in the pH 8.5-K^+ dataset. This indicates that at pH 8.5, binding of Na^+ drives NhaA towards the open conformation where the NT is unplugged.

3) cpH-MD calculations:

* What are the estimated pKa's for all residues listed in Suppl Fig 4?

- We thank the reviewer for their comment. The estimated pK_a values are now reported in Supplementary Table 3 and referenced in the main text.

* Are these calculations converged? For instance, calculate pKa's as a function of cumulative simulated time.

- The convergence of pK_a values for selected residues as well as that of the C_α RMSD is now shown in Supplementary Figs. 5 and 7. Additionally, the extracted pK_a values are robust, and change negligibly when the first 500 ns of each simulation are discarded instead of the first 200 ns.

4) Fig 3e: What is the evidence that the "elongated density blob" between D164 and T132 in the IF-apo[open funnel] (middle) structure is not a K+ ion whereas the "spherical density" in the IF-Na+ structure is indeed a Na+ ion?

- The typical distances of Na^+-Asp carboxyl group is ~2.4 Å, Na^+-Thr backbone carbonyl ~2.4 Å, and K^+-Asp carboxyl group ~2.8 Å[2]. The distances between the center of the "elongated density" in the IF-apo$^{open\text{-}funnel}$ and the carboxyl groups of Asp163 and Asp164 exceed 4 Å. In addition, previous studies have demonstrated that NhaA neither transports nor binds K^+[3,4]. Therefore, the density found at the ion-binding site of the IF-apo$^{open\text{-}funnel}$ is not K^+. On the other hand, the distances between the "spherical density" observed in the IF-Na^+ state and the ion-binding residues match typical Na^+ coordination geometry (see updated Fig. 3e), suggesting that the density is likely a Na^+ ion. We have included the statements above into the Results section, which now reads:

  "This density cannot be attributed to K^+, as the distances of the density to the carboxyl groups of Asp163 and Asp164 exceed 4 Å, which is longer than a typical K^+–Asp coordination distance (~2.8 Å)[51]. Furthermore, NhaA neither binds nor transports K^+[42,52]. … Consistently, the observed Na^+–ion-binding residue distances match typical Na^+ coordination geometry (~2.4 Å to Asp and ~2.4 Å to the Thr backbone carbonyl)[51] (Fig. 3e)."

Admittedly, the structural data and the MD at pH 8.5 seem to be agreeing pretty well. Is there additional evidence from the MD simulations, e.g. do Na+ spontaneously bind to IF-apo[open funnel] and generate the IF-Na+ binding mode?

- We started the cpH-MD simulations from both the low pH structure (PDB 4AU5) and the IF-Na^+ structure in this study. Since the overall structure of the IF-Na^+ state is highly similar to the IF-apo$^{open\text{-}funnel}$ structure (C_α RMSD = 0.3), also at the ion-binding site (Fig. 3d), we assume that the simulations started from the IF-Na^+ structure, where the bound Na^+ was removed, resembled simulations starting from the IF-apo$^{open\text{-}funnel}$ state. We have made a video showing an instance of a Na^+ ion spontaneously binding to the ion-binding site (Supplementary Video 1) and referenced it in the manuscript.

5) Fig 3g: Add protons for the most likely protonation states of D164, D163, D133, K300. (Otherwise it looks as if they are all deprotonated which does not seem to reflect the data in Suppl Fig 4.)

- We thank the reviewer for pointing this out. We have added protons to the amino acid residues shown in Fig. 3g to reflect the most likely protonation states as shown in Fig. 3j and Supplementary Fig. 7.

6) Suppl Fig 4: cpH-MD:

* Asp164 is protonated even above pH 9 in the absence of Na+ near the binding site, even though the residue is water accessible. Given that the pKa of Asp in water is ~4, retaining a proton up to pH 9.5 is a bit surprising. Which interactions stabilize the

protonated Asp? Similarly, which interactions maintain the protonation of Asp163 over the whole pH range (without Na+)?

- We are grateful for the reviewer's question, which prompted us to think about these remarkable protonation states in more detail. While the identity of the exact proton carrier residues is not known, Asp164 is widely regarded as one of them, with Asp163 or Lys300 being the other. Consequently, to elicit electrogenic proton transport, these residues have to keep their activity over the entire pH range. Therefore, from a holistic viewpoint, the protonation state of Asp163 and Asp164 is a requirement of the "two-aspartate" mechanism.

- To investigate the molecular determinants that stabilize the protonation state of Asp163 and Asp164, we examined the surrounding environments of both residues in our simulation data, and found that Asp164 is mainly stabilized by the hydrophobic environment and Asp163 by hydrogen bonds (Revision Fig. 1). We have added the following text in the Results section:

  "When Na^+ entry is blocked, Asp164 remains protonated due its predominantly hydrophobic environment and limited hydration (< 2.0 waters) (Supplementary Fig. 6c). Asp163 also remains protonated without Na^+ in the ion-binding site, forming hydrogen bonds with the hydroxyl group of Thr132 and the backbone of Ala130, although it occasionally reorients toward a more hydrophobic region (Supplementary Fig. 6d)."

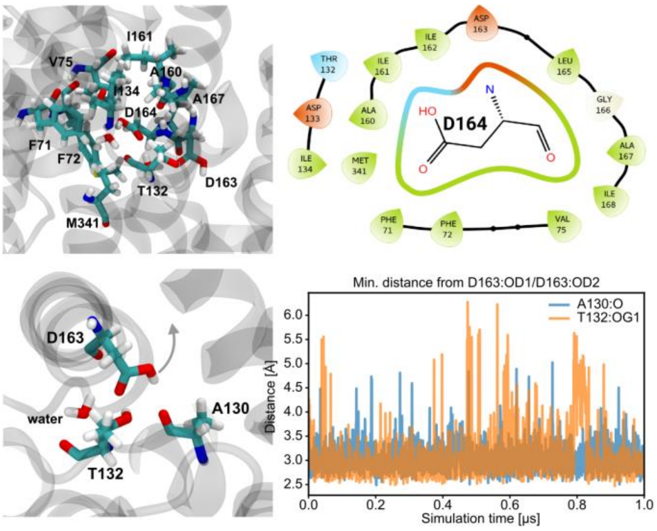

Revision Fig. 1. Stabilization of the protonation states of Asp164 and Asp163 in MD simulations. (See also Supplementary Fig. 6.)

* It looks as if K300 is always protonated. Which interactions stabilize K300 in its charged state, especially given that D163 is mostly protonated and thus neutral and can only form a hydrogen bond? It appears physically inconsistent that a negative charge on D163 is unfavorable while the positive charge on K300 (even deeper inside the protein) is favored in the cpH-MD simulation.

- We thank the reviewer for the question. The positive charge on Lys300 is stabilized by the negatively charged C-termini of TMs IVp and XIc. Likewise, the negative charge on Asp133 is stabilized by the positively charged N-termini

of TMs IVc and XIp (Revision Fig. 2). We have added the following text in the Results section:

"The simulations indicate a strong preference for an ion-binding site in which Asp163, Asp164, and Lys300 are all protonated, and Asp133 is deprotonated in the absence of Na^+ (Fig. 3j). The arrival of Na^+ triggers deprotonation of the aspartates, first of Asp164 and then of Asp163 (Fig. 3j). The protonation states of Asp133 and Lys300 are stabilized by their electrostatic interactions with the positively charged N-termini of TMs IVc and XIp, and the negatively charged C-termini of TMs IVp and XIc, respectively (Supplementary Fig. 6a,b)."

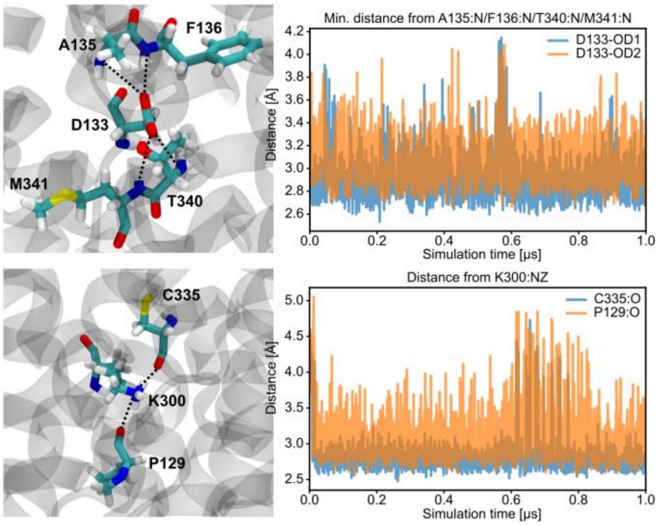

Revision Fig. 2. Stabilization of the protonation states of Asp133 and Lys300 in MD simulations. (See also Supplementary Fig. 6.)

7) Please clarify what "Na+ not blocked from cytoplasmic funnel" means for the presence of Na+ near the binding site. What fraction of the simulation time is Na+ bound to D164 and/or D163?

- We are grateful for the reviewer for pointing out our omission. In the "Na^+-blocked" case, the Na^+ ion was explicitly prevented to interact with the ion-binding site residues Asp133, Asp163, Asp164, and Lys300 through the use of a flat-bottomed potential. This is now clarified in the text as:

  "All simulations were repeated with a layer-like flat-bottomed position restraint (k = 200 $kJ/mol/nm^{-2}$) applied to all Na^+ ions to block them from entering the active site, and simulated for 1 μs."

  Additionally, we added the Na^+ occupancy of the ion-binding site to Fig. 3j, which shows strong correlation with the protonation state of the ion-binding residues Asp163 and Asp164.

Reviewer #3 (Remarks to the Author):

This comprehensive study by Weng, Fabian and colleagues reports the pH dependent structural rearrangements of Escherichia coli NhaA, a prototypical sodium-proton exchanger. Using single particle cryo-EM of NhaA dimers in complex with a conformation-specific monoclonal antibody fragment, the authors identified several previously unrevealed structural features such as the asymmetric binding of two different lipids at the symmetric dimer interface and the N-terminal tail, which can act

as a plug to seal the transport pathway. Population analysis of particles imaged at pH conditions ranging from 5.5 to 8.5 reveal that the occupancy of the N-terminal plug is pH-dependent, with open state being dominant at pH 8.5. Consistent with the cryo-EM analysis, the N-terminal tail of NhaA can stably occupy the pathway in molecular dynamics simulations. The structures and the molecular dynamics simulations show that the funnel of NhaA gradually opens by breaking a bond between E78 and H256 as the pH increases, revealing how pH regulates transports activity. This paper is well-written and represents a significant advance in the understanding of the activation of NhaA. I have several comments listed below that should be addressed prior to publication that would strengthen the report.

Comments

Major points

1. One of the major strengths of this report is the diversity of functional states presented. However, on line 25 the authors note that the structures were determined in complex with a conformation-specific antibody fragment. What are the functional effects of the Fab?

- As described in the first paragraph of the Results, the variable fragment (Fv) 6F9 was derived from the monoclonal antibody (mAb) 6F9, which binds to the detergent solubilized, native NhaA but not the denatured protein on Western blots[5]. Epitope mapping shows that mAb6F9 recognizes the dispensable C-terminus of NhaA[6]. In addition, mAb6F9 had no effect on the antiport activity of affinity purified NhaA reconstituted in proteoliposomes[5]. Neither the initial rate of $^{22}Na^{+}$ uptake nor the steady state level of $^{22}Na^{+}$ accumulation were affected.

If the Fab is indeed conformation-specific, how was the ensemble of states presented here captured? The authors should evaluate the functional role of the Fab and clarify its effects on transport to ensure that the ensemble of states presented here correspond to physiologically relevant states.

- To avoid confusion, we have modified the text, and it now reads:

  "The variable fragment of a non-inhibitory monoclonal antibody 6F9 (Fv6F9), which binds to the dispensable C-terminal tail of native NhaA[40,41], was used to form a complex with NhaA (Extended Data Fig. 1)."

2. The use of symmetry expansion to resolve an asymmetric arrangement of the lipids at the dimer interface is remarkable. Were there any alternative populations present among the particles or did every particle have the same combination of lipids at the interface?

- We did not observe any alternative populations that have different combination of lipids at the dimer interface.

Additionally, were there any changes in the conformation of the protein in response to the asymmetric positioning of the lipids?

- As the cryo-EM structure of the consensus IF-apo NhaA at pH 7.5 is highly symmetric, we first built the model for protomer A, refined in PHENIX, and fitted the same model in the density of protomer B to build the complete dimer model. Model-to-map fitting was carefully checked and adjusted if needed, and we did

not find major differences between the two protomers. However, we agree with the reviewer that we should examine cautiously any changes caused by the asymmetric positioning of the lipids. To address this, we have refined the dimer model in PHENIX, examined the model-to-map fitting and adjusted the model if necessary. The resulted model shows improved model-to-map correlation coefficient (0.88) compared to the previous model (0.80). Superposition of the two protomer structures shows nearly the same conformation with a C_α RMSD of only 0.08 Å. We did not find any conformational differences at the ion-binding site and the pH sensor, except for a shift of Arg250 sidechain at the dimer interface, which is likely due to the asymmetric positioning of the phosphate groups of the cardiolipin (Revision Fig. 3). We have included this figure in Supplementary Fig. 3, and added the corresponding sentences in Results:

"Both protomers adopt nearly identical conformations with a C_α root mean square deviation (C_α RMSD) of 0.08 Å (Supplementary Fig. 3a). The only detectable difference is a slight shift of the Arg250 sidechain at the dimer interface, likely caused by asymmetric positioning of the CL phosphate headgroups (Supplementary Fig. 3b)."

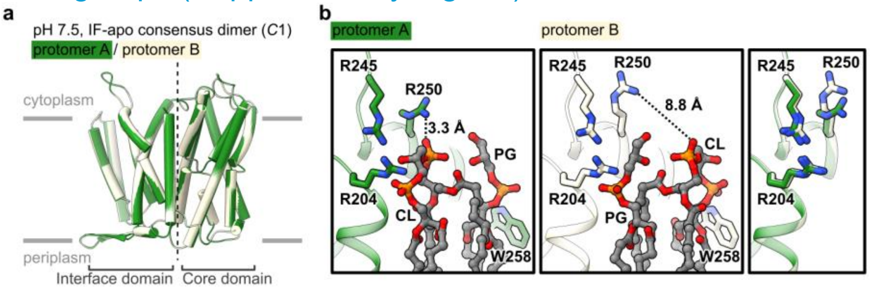

Revision Fig. 3. Structural comparison of the two NhaA protomers and the dimer interface. (See also Supplementary Fig. 3.)

3. How were densities assigned as ions or water molecules? At the reported overall resolutions of 2.7 to 3.7 Å, it can be different to unambiguously assign non-protein densities. For example, there are densities in middle panel of Fig 3e that are very similar to those present in the right panel, but a Na+ and a water are assigned in only the right panel. It would be helpful to clarify these details and perhaps include local resolution maps to demonstrate that the features are sufficiently well resolved for modelling.

- We thank the reviewer for the suggestion. After carefully examining the local-resolution-filtered maps of the IF-apo$^{\text{open-funnel}}$ and IF-Na^+ states (Revision Fig. 4), we found that at the ion-binding site of the IF-Na^+ state, the two densities are at ~2.9 Å resolution and show clear spherical shape and at the positions coincide with that of the Na^+ and water bound to the ion-binding site in our MD simulations. On the other hand, although the local resolution of the elongated densities at the ion-binding site of the IF-apo$^{\text{open-funnel}}$ state is at slightly higher resolution (~2.85 Å), the densities are not as distinguishable as that in the IF-Na^+ state. This indicates that the molecules occupying the space at the ion-binding site of the IF-apo$^{\text{open-funnel}}$ state do not have distinct coordination with the ion-binding residues and thus flexible in position, implying that they are highly likely to be water molecules. We have included this figure in the Supplementary Information file and modified the corresponding text, which now reads:

"The ion-binding site of the Na^+-supplemented structure, resolved locally at ~2.9 Å resolution, shows a clear spherical extra density between the putative ion-binding residues Asp163 and Asp164 on TM V as well as Thr132 on loop IVp-IVc (Fig. 3e, Supplementary Fig. 4a). In contrast, the ion-binding site of the IF-apo$^{open\text{-}funnel}$ structure shows an elongated density, resolved locally at ~2.85 Å resolution, likely emerging from water molecules entering the binding site (Fig. 3e, Supplementary Fig. 4b)."

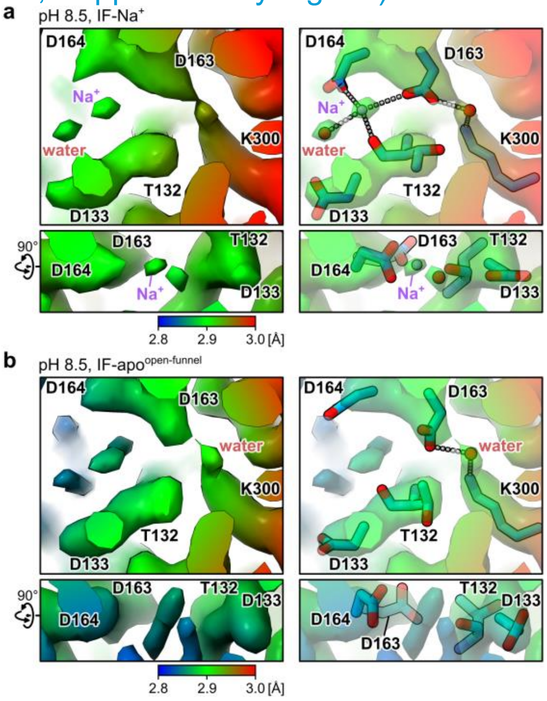

Revision Fig. 4. Local-resolution map of the ion-binding site in the IF-Na^+ (**a**) and IF-apo$^{open\text{-}funnel}$ (**b**) states (See also Supplementary Fig. 4).

Minor points

1. Line 172. Is Fig 2d correct? Not Fig 2 b,c?

- We have corrected the figure reference to Fig. 2b,c.

2. Line 174. Fig 2d need to be here?

- We have added the figure reference Fig. 2d here.

3. It would be helpful to readers to include an overall description of the important features of the structure when you describe overall structure. For example, the "β-hairpin" is first presented on line 199 when it is being evaluated in detail.

- We thank the reviewer for the suggestion. In the Introduction where the overall architecture of NhaA is described, we added a sentence about the β-hairpin, which reads:

  "At the dimer interface, a β-hairpin between TMs I and II from each protomer forms an interfacial β-sheet at the periplasmic side, which has been suggested to stabilize the dimeric assembly[21,22] (Fig. 1a,b)."

Reviewer #4 (Remarks to the Author):

The manuscript by Weng, Michel and colleagues reports the cryo-EM structures of the Escherichia coli Na^+/H^+ antiporter NhaA, capturing its conformational ensemble across a physiological pH range. The structural determination is executed with high quality, and the authors delineate distinct conformational states corresponding to different pH conditions, thereby revealing the molecular transitions underlying pH-dependent activation. Notably, the visualization of the previously unresolved N-terminal tail represents a significant advancement, providing new insight into the gating and activation process of NhaA.

Major:

1. It is proposed that the N-terminal tail (NT) dissociates as pH increases, a notion further supported by molecular dynamics simulations. However, functional assays indicate that the NT mutant exhibits activity comparable to the wild type. This apparent discrepancy warrants further investigation.

- We agree with the reviewer. It is not clear why there is no difference in the functional activity of the WT and the ΔNT. The function of the NT may be involved in conditions other than what we presented in our manuscript. We therefore have decided to focus on solving the long-sought structure of outward-facing NhaA computationally and experimentally. This may give us a new research direction. Since this work is not yet completed, we did not show the results in the present manuscript.

2. Molecular dynamics simulations were employed to identify the sodium-binding site. To definitively confirm its functional role, it would be compelling to complement these computational findings with experimental mutagenesis of the key residues.

- We agree with the reviewer. In fact, previous biochemical and mutational studies have identified evolutionary conserved Asp163, Asp164 and Thr132, which are the key residues involved in Na^+ binding and transport. Asp163 and Asp164 are essential for NhaA activity, as mutations Asp163Cys and Asp164Cys completely abolish transport[7]. Likewise, substitution of Thr132 to cysteine increases the apparent K_m by tenfold and slows growth in selective media[7], consistent with coordination of Na^+ through the backbone carbonyl rather than the sidechain. These findings are further supported by ion-binding measurements using isothermal titration calorimetry with Li^+[8] and direct $^{22}Na^+$ binding assays[4]. To clarify this, we have added sentences in the Results section, which now read:

  "Sequence conservation and mutagenesis studies indicate that NhaA binds Na^+ through the conserved residues Asp163, Asp164 and Thr132[42,47–50]. …… Previous mutagenesis analyses showed that substitutions at Asp163 and Asp164 abolish Na^+ or Li^+ binding and transport, whereas mutations at Thr132 only reduce activity, consistent with our structural observations that Thr132 coordinates Na^+ via its backbone carbonyl rather than the sidechain[42,47–49]."

3. The author mentioned that obtaining the structure of IF-apo open-funnel state may be the optimization of purification conditions. Given that all resolved structures correspond to inward-facing conformations, exploring alternative approaches to stabilize or visualize the outward-facing state would further enhance the understanding of the complete transport cycle.

- We thank the reviewer for the suggestion. Other than the cryo-EM samples shown in this study, we have tried many other methods, e.g. using detergents instead of nanodiscs, and trying different buffer conditions. However, we could still only obtain inward-facing conformations. We are currently working on getting the long-sought outward-facing conformation computationally and experimentally and are planning to put these results into our next manuscript.

4. The authors note that the interfacial lipids between dimers could be assigned to different classes depending on the classification scheme and symmetry applied. However, the basis for these assignments requires further clarification: The reasoning behind the suggestion of dimerization interface asymmetry, based solely on "neighboring densities resembling conventional diacyl phospholipids," is unclear. A more detailed explanation is needed. Please clarify the specific criteria used to distinguish cardiolipin (CL) density from that of diacyl phospholipids.

- A cardiolipin density could be distinguished from typical phospholipid according to their number of acyl chains: a cardiolipin molecule has four acyl chains, whereas a typical phospholipid molecule has two. To improve clarity, we have modified the sentences and they now read:

  "Cardiolipin (CL), a tetra-acyl lipid, has been shown to be essential for NhaA dimerization[43,44]. …… The density could not be fitted with either one or two CL molecules, suggesting an asymmetric lipid arrangement at the dimer interface."

Minor:

1. The middle panel of Figure e in Fig3 should be the structure of IF-apo-funnel. There are also two blocks between D164 and T132 whose density is similar to the density of the sodium ion binding position in the right figure. How the position of the Na+ binding sodium ion is determined？

- The middle panel of Fig. 3e is the structure of IF-apo$^{open\text{-}funnel}$. We have added text in the figure to clearly label the structures. The position of bound Na^+ ion is determined based on our MD simulation data (Fig. 3g), and previous biochemistry and mutagenesis studies that suggested the putative ion-binding site consists of the sidechains of Asp163, Asp164 and the backbone carbonyl of Thr132, as stated in the manuscript. To distinguish the densities in the IF-apo$^{open\text{-}funnel}$ and IF-Na^+ states, we carefully examine the local-resolution-filtered maps of both states. As shown in Revision Fig. 3, and the response to Reviewer #3, the two densities of the IF-Na^+ state show clear spherical shape and at the positions coincide with that of the Na^+ and water bound to the ion-binding site in our MD simulations. On the contrary, the densities of the IF-apo$^{open\text{-}funnel}$ state are not as distinguishable as that in the IF-Na^+ state. This indicates that the molecules occupying the space at the ion-binding site of the IF-apo$^{open\text{-}funnel}$ state do not have distinct coordination with the ion-binding residues and thus flexible in position, implying that they are highly likely to be water molecules.

2. In the supplementary tables, the listed "Map sharpening B factor" is the Gaussian temperature factor applied to the final reconstruction to restore high-resolution contrast. By convention it is reported as a positive number (units Å²); a negative sign simply means the map was over-sharpened (i.e. an artificial negative B was added). Please double-check the actual B-factor values and report them accurately.

- As the reviewer mentioned, cryo-EM maps without any post-processing usually look blurrier than an ideal map with the same resolution due to signal decay at high frequencies contributed by several factors. The B factor used in cryo-EM to describe the attenuation of signal is similar to the one in X-ray crystallography and is thus positive. In practice, a negative B factor is applied to restore high frequency signal in a cryo-EM map, hence it is called "map sharpening B factor". This is discussed in detail in *Single-Particle Cryo-EM of Biological Macromolecules*[9]. Furthermore, negative map sharpening B factors were reported in many most recent publications[10–12].

3. In addition, the authors imposed C2 symmetry at an early stage of the processing workflow. For the pH 5.5–K+ dataset, Fv6F9 density is absent from one protomer before C2 symmetry is applied, suggesting that the two NhaA monomers may adopt distinct conformations. Imposing C2 symmetry at the outset might force these different states into a single, artefactual average. It is recommended re-processing this dataset in C1, examining class distributions carefully, and providing a clear explanation for why Fv6F9 is missing on one side before any symmetry is enforced.

- Although the datasets were processed with *C*2 symmetry in the middle stage, which indeed could force potential heterogeneity into one single average, we applied symmetry expansion at a later stage to ensure that each protomer particle was treated individually and thus recover the different states within NhaA dimer. In addition, Fv6F9 does not affect NhaA activity[5]. Our cryo-EM structures show that Fv6F9 recognizes the C-terminus (Arg385) and cytoplasmic side of TM XIc (Gln327) of NhaA and does not interact with the NT. Thus, Fv6F9 should not affect the conformations of NhaA reported in this study.

4. What are the other bands corresponding to Peak A, Peak B, and Peak C on the SDS-PAGE gel in Extended Data Fig. 1?

- The high molecular weight bands of Peak A, B and C came from the NhaA dimer or aggregates likely due to heating of the sample before the SDS-PAGE, as described by Taglicht et al.[13]. We have added labels in Extended Data Fig. 1d and added a sentence in the figure caption to clarify this.

5. Conventionally, the periplasm is oriented upward and the cytoplasm downward in structural figures, as this aligns with standard visual conventions in membrane biology. If the authors opt not to adjust the orientation, they should provide comparative example figures to explicitly justify their chosen layout (e.g., to better illustrate specific structural details or functional processes).

- We understand that it is a visual convention to show the cytoplasm downward when displaying structural figures of membrane proteins. However, the layout of cytoplasm oriented upward for NhaA has been used since the publication of its first crystal structure[14] and in following structural studies[1,15,16]. To follow these examples, we would like to keep the current membrane orientation in our figures.

6. The scale bar for each cryo-EM micrograph in the processing workflow (Supplementary Fig. 1 a-e) should be added.

- We thank the reviewer for pointing this out. We have added the scale bars for the cryo-EM micrographs in Supplementary Fig. 1.

7. The "Supplementary Fig. 14" mentioned on lines 478 and 493 may not be included in the available materials; please provide the correct citation.

- We have corrected the figure reference to Extended Data Fig. 10a.

8. CαBLAM number is missing from the Cryo-EM table.

- We have added the CαBLAM number for each reported cryo-EM structure in Supplementary Table 2.

9. The angular distribution of particles used in the final 3D reconstruction should be shown.

- We have added the angular distribution of particles for each reported cryo-EM structure in Supplementary Fig. 2.

Reviewer #5 (Remarks to the Author):

I co-reviewed this manuscript with one of the reviewers who provided the listed reports. This is part of the Nature Communications initiative to facilitate training in peer review and to provide appropriate recognition for Early Career Researchers who co-review manuscripts.

References:

1. Huang, Y., Chen, W., Dotson, D. L., Beckstein, O. & Shen, J. Mechanism of pH-dependent activation of the sodium-proton antiporter NhaA. *Nat Commun* **7**, 12940 (2016).
2. Bazayeva, M., Andreini, C. & Rosato, A. A database overview of metal-coordination distances in metalloproteins. *Acta Crystallogr D Struct Biol* **80**, 362–376 (2024).
3. Eisinger, M. L., Dörrbaum, A. R., Michel, H., Padan, E. & Langer, J. D. Ligand-induced conformational dynamics of the Escherichia coli Na+/H+ antiporter NhaA revealed by hydrogen/deuterium exchange mass spectrometry. *Proc Natl Acad Sci U S A* **114**, 11691–11696 (2017).
4. Quick, M., Dwivedi, M. & Padan, E. Insight into the direct interaction of Na+ with NhaA and mechanistic implications. *Sci Rep* **11**, 7045 (2021).
5. Padan, E., Venturi, M., Michel, H. & Hunte, C. Production and characterization of monoclonal antibodies directed against native epitopes of NhaA, the Na^+/H^+ antiporter of *Escherichia coli*. *FEBS Lett* **441**, 53–58 (1998).
6. Rimon, A., Hunte, C., Michel, H. & Padan, E. Epitope mapping of conformational monoclonal antibodies specific to NhaA Na^+/H^+ antiporter: structural and functional implications. *J Mol Biol* **379**, 471–481 (2008).
7. Galili, L., Rothman, A., Kozachkov, L., Rimon, A. & Padan, E. Trans membrane domain IV is involved in ion transport activity and pH regulation of the NhaA-Na(+)/H(+) antiporter of Escherichia coli. *Biochemistry* **41**, 609–617 (2002).
8. Maes, M., Rimon, A., Kozachkov-Magrisso, L., Friedler, A. & Padan, E. Revealing the Ligand Binding Site of NhaA Na+/H+ Antiporter and Its pH Dependence. *J Biol Chem* **287**, 38150–38157 (2012).
9. Glaeser, R. M., Nogales, E. & Chiu, W. *Single-Particle Cryo-EM of Biological Macromolecules*. (IOP Publishing, 2021).
10. Kikuchi, A. *et al.* Cryo-EM reveals evolutionarily conserved and distinct structural features of plant CG maintenance methyltransferase MET1. *Nat Commun* **16**, 8524 (2025).
11. Zhang, F., Li, S., Wu, H. & Chen, S. Cryo-EM structure and oligomerization of the human planar cell polarity core protein Vangl1. *Nat Commun* **16**, 135 (2025).
12. Fan, C., Cowgill, J., Howard, R. J. & Lindahl, E. Cryo-EM structures of ρ1 GABAA receptors with antagonist and agonist drugs. *Nat Commun* **16**, 7077 (2025).
13. Taglicht, D., Padan, E. & Schuldiner, S. Overproduction and purification of a functional Na^+/H^+ antiporter coded by *nhaA* (*ant*) from *Escherichia coli*. *J Biol Chem* **266**, 11289–11294 (1991).
14. Hunte, C. *et al.* Structure of a Na^+/H^+ antiporter and insights into mechanism of action and regulation by pH. *Nature* **435**, 1197–1202 (2005).
15. Arkin, I. T. *et al.* Mechanism of Na+/H+ antiporting. *Science* **317**, 799–803 (2007).

16. Appel, M., Hizlan, D., Vinothkumar, K. R., Ziegler, C. & Kühlbrandt, W. Conformations of NhaA, the Na+/H+ exchanger from Escherichia coli, in the pH-activated and ion-translocating states. *J Mol Biol* **388**, 659–672 (2009).

REVIEWER COMMENTS

Reviewer #1 (Remarks to the Author):

The authors have adequately addressed my comments.

- We thank the reviewer for their constructive advice on our MD simulations. We are glad that the reviewer's concerns have been addressed.

Reviewer #2 (Remarks to the Author):

The authors addressed my previous comments. Thank you.

- We are pleased to hear that we have successfully addressed the reviewer's comments.

Minor correction:

Suppl Fig 10: Caption says "displaying standard deviations of Na+ occupancy and deportonation". This seems ambiguous: It looks to me as if the occupancy and deprotonation is shown and error bars correspond to one SD.

- We have made the caption for Supplementary Fig. 10 clear and explicit, and it now reads:
  "Separate plots displaying the Na^+ occupancy and the deprotonation of Asp164 and Asp163 in cpH-MD simulations with freely moving Na^+ (corresponding to Fig. 3j), with error bars representing one standard deviation."

Comments:

Glu78: Winkelmann et al, Nature Comm 2022 include it in the sensor residues and show that the E78A mutant has a different pH activation profile WT, supporting the authors interpretation of Glu78 as a pH sensor residue.

- We thank the reviewer for pointing this out. We have added one sentence in the following paragraph in the Results section:
  "Glu78 starts titrating at acidic pH and becomes deprotonated at alkaline pH, while His256 remains positively charged at lower pH and becomes fully deprotonated above pH 8.0, suggesting a transient Glu78–His256 salt bridge at lower pH, which breaks at higher pH values (Supplementary Fig. 15). Supporting this, the previous study shows that Glu78Ala mutant has a different pH activation profile than the WT[35]."

Reviewer #3 (Remarks to the Author):

The authors have addressed all concerns and the manuscript is suitable for publication.

- We are glad that all concerns from the reviewer have been successfully addressed.

Reviewer #4 (Remarks to the Author):

The authors have addressed the previous comments by providing additional clarifications and supporting references. These revisions improve the clarity and presentation of the manuscript.

- We are pleased that the reviewer's comments have been satisfactorily addressed.

minor:

1.The authors stated that scale bars have been added to Supplementary Figure 1; however, these are still absent from the revised figure.

- We thank the reviewer for pointing out this mistake. We have confirmed that the figure is now updated accordingly.

2. For clarity and reproducibility, please confirm that the exact map sharpening B-factor values applied to each reconstruction are explicitly reported in the Methods section.

- We have added this in the Methods section for clarity:
  "Two-dimensional classification, initial model generation, three-dimensional (3D) classification, 3D refinement, Bayesian polishing, CTF refinement, and final map reconstruction with map sharpening B-factors shown in Supplementary Table 2 were done using RELION 4[83]."

Reviewer #5 (Remarks to the Author):

I co-reviewed this manuscript with one of the reviewers who provided the listed reports. This is part of the Nature Communications initiative to facilitate training in peer review and to provide appropriate recognition for Early Career Researchers who co-review manuscripts.
